# Empirical landscape genetic comparison of single nucleotide polymorphisms and microsatellites in three arid‐zone mammals with high dispersal capacity

**DOI:** 10.1002/ece3.10037

**Published:** 2023-05-02

**Authors:** Ebony D. Skey, Kym M. Ottewell, Peter B. Spencer, Robyn E. Shaw

**Affiliations:** ^1^ Environmental & Conservation Sciences Murdoch University Perth Western Australia Australia; ^2^ Biodiversity and Conservation Science Department of Biodiversity, Conservation and Attractions Perth Western Australia Australia; ^3^ Present address: Division of Ecology and Evolution, Research School of Biology The Australian National University Canberra Australian Capital Territory Australia

**Keywords:** arid zone, isolation‐by‐resistance, landscape genetics, microsatellites, single nucleotide polymorphisms, small mammals

## Abstract

Landscape genetics is increasingly transitioning away from microsatellites, with single nucleotide polymorphisms (SNPs) providing increased resolution for detecting patterns of spatial‐genetic structure. This is particularly pertinent for research in arid‐zone mammals due to challenges associated with unique life history traits, such as boom‐bust population dynamics and long‐distance dispersal capacities. Here, we provide a case study comparing SNPs versus microsatellites for testing three explicit landscape genetic hypotheses (isolation‐by‐distance, isolation‐by‐barrier, and isolation‐by‐resistance) in a suite of small, arid‐zone mammals in the Pilbara region of Western Australia. Using clustering algorithms, Mantel tests, and linear mixed effects models, we compare functional connectivity between genetic marker types and across species, including one marsupial, *Ningaui timealeyi*, and two native rodents, *Pseudomys chapmani* and *P. hermannsburgensis*. SNPs resolved subtle genetic structuring not detected by microsatellites, particularly for *N. timealeyi* where two genetic clusters were identified. Furthermore, stronger signatures of isolation‐by‐distance and isolation‐by‐resistance were detected when using SNPs, and model selection based on SNPs tended to identify more complex resistance surfaces (i.e., composite surfaces of multiple environmental layers) in the best‐performing models. While we found limited evidence for physical barriers to dispersal across the Pilbara for all species, we found that topography, substrate, and soil moisture were the main environmental drivers shaping functional connectivity. Our study demonstrates that new analytical and genetic tools can provide novel ecological insights into arid landscapes, with potential application to conservation management through identifying dispersal corridors to mediate the impacts of ongoing habitat fragmentation in the region.

## Graphical abstract

We evaluate landscape connectivity using two different genetic marker types across arid‐zone mammals, which are underrepresented in the landscape genetics literature. We find that topography, substrate, and soil moisture are the main environmental drivers shaping connectivity and that single nucleotide polymorphism (SNP) markers provide finer‐detailed ecological insight. To our knowledge, our study is the first to compare SNPs and microsatellites to empirically test isolation‐by‐resistance hypotheses.

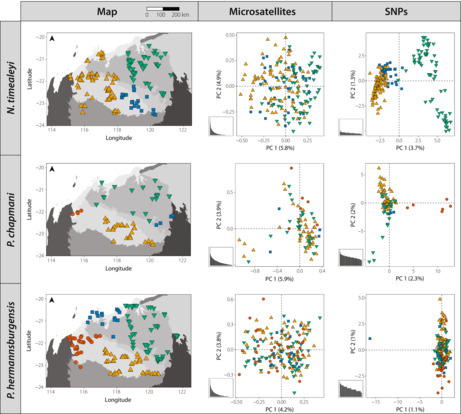

## 1 INTRODUCTION

To conserve biodiversity, it is essential to preserve the evolutionary processes that support it, such as dispersal, mating, gene flow and selection (Latta, 2008). Incorporating dispersal knowledge into conservation planning is fundamental as this represents where and when species move in the landscape (Driscoll et al., 2014). However, deriving dispersal estimates through methods such as capture‐recapture or telemetry is expensive and difficult in non‐abundant species (Waits et al., 2015). Furthermore, the distinction between long‐distance dispersal events that lead to gene exchange (realized dispersal), crucial for maintaining functional connectivity between populations, versus home‐range movements is challenging to ascertain (Jordano, 2017). A valuable and important proxy for measuring functional connectivity is the empirical estimation of gene flow using genetic data since this is equivalent to measuring realized dispersal (Manel et al., 2003; Whitlock & McCauley, 1999).

Landscape genetics aims to determine how realized dispersal is influenced by the surrounding environment. It operates by examining genetic variation within heterogenous landscapes to explicitly quantify the effect of landscape composition and/or matrix quality (the space separating habitat patches) on an organism's dispersal, gene flow and/or spatial‐genetic structure (Manel et al., 2003). Microsatellite markers have previously been the major tool for such research (Storfer et al., 2010), capitalizing on short nucleotide motifs that are repeated in tandem a variable number of times. Due to high mutation rates, they possess a high information content per locus (Morin et al., 2004), although panels are often constrained to just tens of markers and represent a small fraction of the genome (O'Leary et al., 2018). Alternatively, single nucleotide polymorphisms (SNPs) are abundant and widespread throughout the genome (Morin et al., 2004). Though SNP markers are bi‐allelic and give lower information content per locus, this can be offset by the large number generated (often thousands to tens of thousands) (Andrews et al., 2016).

With appropriate sampling, microsatellites have revealed patterns of functional connectivity and landscape barriers to dispersal across a variety of species and ecosystems (Emaresi et al., 2011; Munshi‐South, 2012; Trénel et al., 2008). However, evidence suggests that SNP markers have higher accuracy and power to detect individual, population and species level patterns of genetic structure (Kim & Roe, 2021; Sunde et al., 2020). SNPs consistently outperform microsatellite markers in comparative studies analyzing population structure and assignment methods, specifically for finer‐scale population genetic structure or species with high levels of gene flow (Jeffries et al., 2016; Puckett & Eggert, 2016; Viengkone et al., 2016). However, to our knowledge, no studies have yet compared findings between marker types in relation to landscape genetic isolation‐by‐resistance hypotheses (IBR; where dispersal is influenced by the degree of landscape resistance) (McRae, 2006).

Advances in genetic markers, technology and landscape genetic methods provide opportunities to resolve patterns in cryptic landscapes, such as the topographically complex Pilbara region, situated in the Australian arid biome. The Pilbara is a biodiversity hotspot that supports rich faunal diversity including both endemic and widespread mammals (McKenzie et al., 2009), yet the functional connectivity in Pilbara mammals is poorly resolved. This is due to several factors such as the boom‐bust population dynamics that many arid‐zone mammals possess, as well as long‐distance dispersal capacities to overcome sharp ecological gradients (Dickman et al., 1995; Kelly et al., 2013). The few genetic studies in the region are based on microsatellite and mitochondrial markers and reveal low genetic structure (Hohnen et al., 2016; Levy et al., 2019; Umbrello et al., 2020). Threats including resource extraction, grazing pressure, and inappropriate fire regimes all impact habitat connectivity in the Pilbara (Cramer et al., 2016), highlighting the need to understand functional connectivity in the region.

Here, we assess spatial‐genetic structure for three small ground‐dwelling mammals (body weight <15 g) adapted to arid environments, including a carnivorous dasyurid marsupial: *Ningaui timealeyi*, and two native rodents: the western pebble‐mound mouse, *Pseudomys chapmani* and the sandy inland mouse, *Pseudomys hermannsburgensis*. While *P. hermannsburgensis* is widespread across most of arid Australia, both *P. chapmani* and *N. timealeyi* are Pilbara endemics. Both *P. hermannsburgensis* and *N. timealeyi* are habitat generalists, although *P. hermannsburgensis* shows a slight preference for sandy substrates (Gibson & McKenzie, 2009). Conversely, *P. chapmani* is a habitat specialist associated with rocky substrates, requiring small, uniform pebbles to construct mounds (Start et al., 2000). Both *Pseudomys* sp. exhibit boom‐bust population dynamics, while *N. timealeyi* displays more seasonal breeding (Dickman et al., 1995; Dunlop & Sawle, 1982; Start et al., 2000).

A previous study found limited evidence that the Pilbara landscape influenced patterns of genetic structure in these species (Levy et al., 2019). With new, high‐resolution genetic and spatial data, we investigate patterns of functional connectivity in the Pilbara using microsatellites and SNPs and qualitatively compare the ability of each data set to resolve (1) population genetic structure and potential physical barriers to dispersal (isolation‐by‐barrier; IBB); and (2) the role of dispersal capacity (isolation‐by‐distance; IBD) and specific landscape attributes (isolation‐by‐resistance; IBR: aridity, soil moisture, substrate, topography, distance to water, vegetation, and/or fire) in facilitating or restricting realized dispersal. We explore how vast and dynamic arid landscapes shape the spatial‐genetic structure of arid‐zone species with high capacity for dispersal, and how new analytical and genetic tools can provide novel ecological insights for conservation.

## 2 MATERIALS AND METHODS

### 2.1 Study area

**FIGURE 1:**
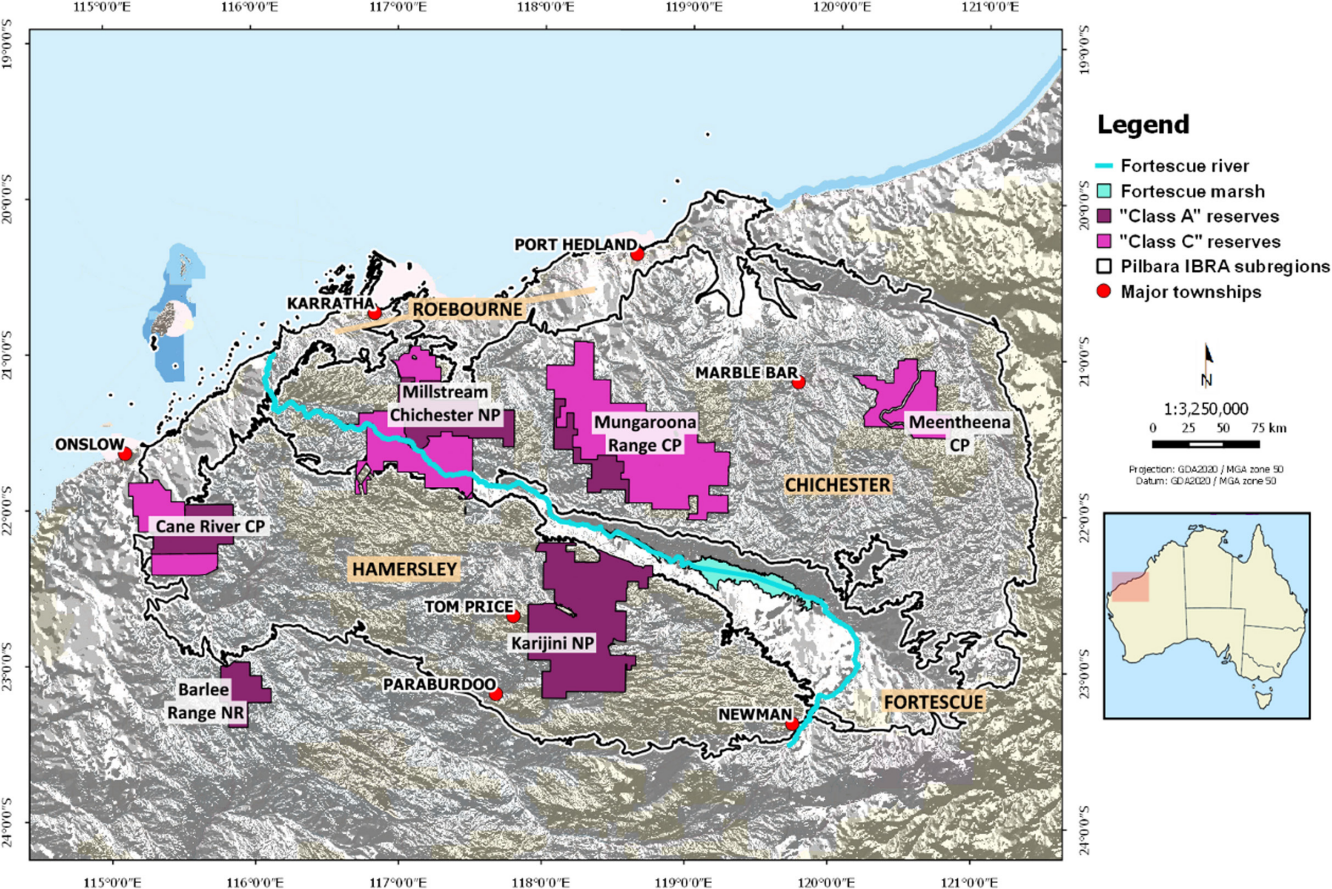
Map of the focal study site located in the Pilbara region of north‐west Western Australia. The greyscale Digital Elevation Model overlaid outlines regional topography. Nature reserves are indicated in purple; where NP = National Park and CP = Conservation Park, and the major Interim Biographic Regionalisation of Australia (IBRA) subregions of the Pilbara are highlighted in orange.

The Pilbara bioregion covers an extensive 179,000 km^2^ and is divided into four distinctive subregions: Chichester, Hamersley, Fortescue, and Roebourne (Figure 1). The Hamersley and Chichester subregions are characterized by rugged ranges (elevation ≤1250 m). Mulga woodland and sedimentary ranges and gorges are found in the former, while the latter is dominated largely by *Acacia* shrub steppe with granite and basalt plains (McKenzie et al., 2009). The alluvial plains of the Fortescue River Valley dissect the two ranges; consisting of extensive marsh and flood‐out zones, and Roebourne is comprised predominantly of sandy coastal plains (McKenzie et al., 2009). The two main bioclimatic zones overlapping the Pilbara include semi‐tropical and arid climates (Sudmeyer, 2016).

### 2.2 Genetic data sets

Microsatellite data for *N. timealeyi* (*Nt*), *P. chapmani* (*Pc*) and *P. hermannsburgensis* (*Ph*) were obtained from Levy et al. (2019) and consisted of 12 loci for *N. timealeyi*, and 14 loci for both *Pseudomys* species. See Levy et al. (2019) for further detail. A subset of these samples were re‐genotyped for SNP loci with Diversity Arrays Technology Pty Ltd (DArT). We focussed on samples collected between 1988 and 2006, resulting in sequencing of 183 individuals for *N. timealeyi* (*Nt*), 94 individuals for *P. chapmani* (*Pc*) and 179 individuals for *P. hermannsburgensis* (*Ph*). A total of 100–500 ng of genomic DNA was sent to DArT for library preparation and sequencing. Library preparation by DArTseq™ follows a reduced representation method with enzyme digestion (here PstI and SphI) followed by sequencing on an Illumina Hiseq 2500 (*Nt*: medium density sequencing at 1.2 million reads; *Pc* and *Ph*: high density sequencing at 2.5 million reads) (Cruz et al., 2013; Kilian et al., 2012). Read assembly, quality control and SNP calling was carried out through DArT's proprietary software (Melville et al., 2017).

The raw SNP data sets (*Nt* = 36,899, *Pc* = 45,733 and *Ph* = 139,916 SNP loci) were filtered in *R* version 4.1.2 (R Core Team, 2021) using a custom R script (Shaw, 2022; Shaw et al., 2022) with functions from *dartr* (Gruber et al., 2018) and *SNPRelate* (Zheng et al., 2012) packages. Filtering thresholds were determined by visualizing the raw data (see Appendix S1) and filtering was completed sequentially in order of appearance in text. First, individual call rate filtering resulted in the removal of one *N. timealeyi* individual (call rate <0.5), and two *P. hermannsburgensis* individuals (call rate <0.7). Locus quality filters were then applied to each data set based on thresholds for missing data (*Nt* & *Ph*: 5%, Pc: 4%), average total read depth (*Nt*: between 20 and 100, *Pc*: between 15 and 25, *Ph*: between 20 and 40), repeatability average (95%), and minimum minor allele frequency (using thresholds that removed singletons: *Nt* & *Ph*: 0.025, *Pc* = 0.05). Multiple SNPs per sequence were removed followed by linkage disequilibrium pruning. We calculated pairwise relatedness (Wang, 2002) in the R package *related* (Pew et al., 2015), using a relatedness threshold of 0.24 to remove additional samples (*Nt* = 2, *Pc* = 14 and *Ph* = 4) to avoid biasing the genetic analyses with closely related individuals (i.e., half‐siblings and above) (Wang, 2018). We included an additional filter to remove loci not in Hardy–Weinberg Equilibrium (HWE) for IBD and population genetics summary statistics using *dartr* to remove SNPs that significantly deviated from HWE assumptions, with a Bonferroni correction. HWE filtering was carried out within genetic clusters (i.e., populations) identified through IBB analysis, described below.

### 2.3 Isolation‐by‐barrier: genetic clustering

The presence of barriers to dispersal in the three focal species was investigated using multiple analyses. Firstly, we ran a Principal Coordinate Analysis (PCoA) (Legendre & Legendre, 2012) in *dartr* to identify natural genetic clusters in the data (based on Euclidean genetic distances). As a preliminary step, we visualized temporal patterns to ensure genetic structure was not related to sample collection year (Appendix S4). Next, we visualized spatial patterns in the data, and these results helped guide the maximum value for *K* (the number of ancestral populations) when using the TESS3 algorithm in the R package *tess3r* (Caye et al., 2016). As opposed to Bayesian clustering programs like *STRUCTURE* that utilize Markov chain Monte Carlo (MCMC) methods (Pritchard et al., 2000), *tess3r* estimates individual ancestry coefficients based on sparse non‐negative matrix factorization algorithms (sNMF) taking geographic information into account (Caye et al., 2016). This algorithm produces similar results to Bayesian clustering methods while being substantially faster (Frichot et al., 2014). Unlike *STRUCTURE* and related models, this approach does not rely on assumptions such as linkage equilibrium and HWE in ancestral populations (Caye et al., 2016). Given our large geographic and temporal spread of samples, we deemed this the most appropriate model for our study system.

We tested *K* values one through seven, with 50 repetitions for each value and the maximum number of optimisation iterations set to 200. We used the default settings for the remaining parameters and masked 10% of the data to use for the cross‐validation. The best performing run with the lowest root mean squared error (RMSE) was presented, with the best value for *K* decided based on the presence of a plateau or change in slope in the cross‐entropy criterion.

### 2.4 Isolation‐by‐distance and summary statistics

We performed tests for IBD and calculated population genetic summary statistics for each genetic cluster identified in the IBB analysis for both microsatellite and SNP data sets. Individuals were assigned to a genetic cluster if the corresponding admixture coefficient proportion was ≥0.7, while individuals were excluded from IBD and summary statistics if they fell below this threshold. We chose this somewhat arbitrary threshold to enable the representation of discrete populations for comparison and these individuals were not excluded from any other analyses. We also removed three *P. hermannsburgensis* individuals located on islands from IBD analysis and summary statistics, given the low likelihood that mainland and island individuals are randomly mating. IBD was investigated with Mantel tests in GenAlEx 6.5 (Peakall & Smouse, 2006, 2012; Smouse et al., 1986) using 999 permutations to determine the significance of the Mantel correlation coefficient (*Rxy*). Summary statistics were then calculated in GenAlEx 6.5 within each genetic cluster, including the number of alleles (*N*_a_), observed heterozygosity (*H*_o_), expected heterozygosity (*H*_e_) and the fixation index (*F*). When more than one genetic cluster was detected, we also calculated *F*_IS_ and *F*_ST_.

### 2.5 Isolation‐by‐resistance

We collated and derived high resolution spatial layers to test IBR landscape genetic hypotheses (Table 1; Appendix S2). We sought to represent aspects of aridity (aridity indices—ADI, ADX, and ADM), landscape productivity (relative soil moisture indices—SOMO29 to 30), substrate (clay, sand, silt, and coarse fragments—CF), topographic features (weathering intensity index—WII, vector ruggedness measure—VRM, digital elevation model—DEM), watercourses (Euclidean distance to water—WAT), vegetation (spinifex density index—SPIN, persistent forest cover—FOR), and fire (fire frequency—FF). The justification for selecting these variables along with definitions, data sources, and relevant citations can be found in Table 1. All layers were aggregated to a 5 km^2^ resolution due to the long‐range dispersal capacities of the focal species. For example, both *Pseudomys* species have displayed long‐range movements of several kilometers, with *P. chapmani* individuals found up to 2 km from neighboring mounds and *P. hermannsburgensis* recorded moving up to 14 km in a 2‐week period (Dickman et al., 1995; Start et al., 2000). Evidence from dasyurid species suggests that long‐range dispersal of several hundreds of meters to several kilometers is also likely for *N. timealeyi* (Dickman et al., 1995).

**TABLE 1:** Spatial layers used to test isolation‐by‐resistance, with proposed mechanisms and hypotheses detailed in the justification column (see Appendix S2 for layer visualization and further information).

Variable	Description	Justification for hypotheses	Included in final variable set	Source
*Aridity indices*: ADI, ADX, ADM	ADI: min. monthly aridity; ADX: max. monthly aridity; ADM: mean annual aridity.	Areas of higher aridity inhibit primary vegetation growth and invertebrate communities (Walsberg, 2000). We predict this will limit gene flow through reduced protection from predators and limiting food resources while dispersing.	None	Harwood et al. (2016)
*Relative soil moisture indices*: SOMO29, SOMO30, SOMO31, SOMO32, SOMO33	SOMO29: max. for all weeks of the year; SOMO30: min. for all weeks of the year; SOMO31: seasonality (weekly standard deviation), SOMO32: mean of quarter with highest average; SOMO33: mean of quarter with lowest average.	Areas of higher soil moisture stimulate primary vegetation productivity and resource availability (Berndtsson et al., 1996). We predict this will facilitate gene flow by providing protection from predators and food resources while dispersing.	MSAT: *Nt*: SOMO29 *Ph*: SOMO31 SNP: *Nt*: SOMO29 *Pc*: SOMO29 *Ph*: SOMO31	Harwood (2019)
*Substrate*: Clay, Sand, Silt, CF	Mean estimated value (%) of each soil type (where CF = Coarse Fragments) between 0 and 60 cm.	Clay is an important predictor of *Nt* occurrence; *Ph* prefers increasingly sandy substrates; *Pc* prefers rocky substrates (Ford & Johnson, 2007; Gibson & McKenzie, 2009). We predict that species' preferred substrate will be easier to move through, thus facilitating gene flow.	MSAT: *Nt*: CF *Pc*: CF *Ph*: Clay SNP: *Nt*: Clay *Ph*: Silt	Holmes et al. (2014)
*Topographic features*: WII, VRM, DEM	WII: Weathering Intensity Index – describes regolith properties (low = unweathered outcrops, high = areas with clays and sands); VRM: Vector Ruggedness Measure – measure for terrain complexity (independent of slope); DEM: digital elevation model.	*Nt* associated with rugged topography; *Ph* commonly occupies gentle topography; *Pc* displays preference for eroded, hilly areas of unweathered bedrock (Ford & Johnson, 2007; Gibson, 2011). We predict that species' preferred topography will be easier to move through, thus facilitating gene flow.	MSAT: *Ph*: VRM SNP: *Nt*: DEM, VRM *Pc*: DEM, VRM, WII *Ph*: DEM, VRM, WII	WII: Wilford (2012) and Wilford and Roberts (2019) VRM: Derived from DEM DEM: Gallant et al. (2011)
*Water courses*: WAT	Euclidean distance (m) to natural perennial water (excludes artificial water points, and inland flats subject to inundation or flooding).	Other mammals in the Pilbara have shown positive association with permanent water, likely due to higher quality habitat (Moore et al., 2019). We predict proximity to water will facilitate gene flow by providing food resources and protection from predators.	SNP: *Nt*: WAT *Pc*: WAT *Ph*: WAT	Derived from: Landgate (2012, 2017, 2019)
*Vegetation*: SPIN, FOR	SPIN: Spinifex density Index – decision rules applied to determine the most likely locations of spinifex dominated grasslands; FOR: Persistent Forest Cover – frequency of occurrence for forest and sparse woody vegetation between 1988 and 2018.	Higher vegetation density (e.g., *Triodia*) provides protection from predators for small mammals (Moseby et al., 2016). We predict this will facilitate gene flow by providing increased protection from predators while dispersing.	SNP: *Nt*: FOR, SPIN *Pc*: SPIN *Ph*: SPIN	SPIN: Derived from Li et al. (2012) and Rampant et al. (2019) FOR: Derived from Furby (2018) and Furby et al. (2007)
*Fire*: FF	FF: Fire frequency – Proportion of years burnt (annual fire scar mapping) between 2000 and 2008	Burnt areas provide less protection from predators (Moseby et al., 2016). We predict areas that are frequently burnt will limit gene flow through increased predation while dispersing.	SNP: *Ph*: FF	Derived using equivalent methods to North Australia and Rangelands Fire Information (2019)

*Note*: Climate layers represent 30‐year averages centred on 1990.

Abbreviations: MSAT, Microsatellites; *Nt*, *Ningaui timealeyi*; *Pc*, *Pseudomys chapmani*; *Ph*, *Pseudomys hermannsburgensis*; SNP, single nucleotide polymorphisms.

The parameterisation of resistance surfaces within landscape genetic analyses has traditionally relied on subjective “expert opinion” which can sometimes lead to inaccuracy (Liu et al., 2018). Furthermore, researchers generally assume a linear relationship between continuous variables and genetic distance despite this often not being the case (Spear et al., 2010). For these reasons, we used a genetic algorithm to parameterise resistance surfaces (i.e., relationship between pairwise genetic and effective distances) and maximum value (i.e., cost ratio) through optimizing for the best transformation with no a priori assumptions (Peterman et al., 2014), and fit this relationship using linear mixed effects models (described below). This was implemented in the R package *ResistanceGA* (Peterman, 2018). Given these analyses are sensitive to contemporary patterns of gene flow, we removed four samples (*Nt* = 1, *Ph* = 3) with missing date information from this analysis. We also removed individuals located on islands (*Ph* = 3) from the analysis, as our aim was to explore how terrestrial landscape attributes influence gene flow (rather than determining whether the ocean is/is not a barrier to dispersal). Shirk et al. (2017), found that Euclidean distance performed well under high dispersal scenarios, with small sample sizes and low genetic structure. Thus, for each species and marker type (microsatellites vs. SNPs) we calculated mean pairwise Euclidean genetic distance for individuals within each 5 km^2^ raster pixel using the R package *ecodist* (Goslee & Urban, 2007). Effective distances were calculated within the *ResistanceGA* package based on random‐walk commute times, equivalent to *CIRCUITSCAPE* “resistance distance” (Klein & Randić, 1993; McRae et al., 2008).

The creation of optimized composite resistance surfaces through *ResistanceGA* can be applied to both categorical or continuous rasters and can be performed either independently or simultaneously across all raster layers. This is achieved by fitting linear mixed effects models with a maximum likelihood population effects parameterisation (MLPE) (Clarke et al., 2002) to the pairwise genetic data (response) and effective distances (predictor). During optimisation, models are compared based on an objective function (we used the default option, log‐likelihood) across different transformations and parameters over “generations” until there is no improvement, thus indicating the best optimized surface.

Before calculating the multi‐surface optimisations, we reduced collinearity in the raster data by removing correlated variables (Spearman's |*r*_s_| > .7; Appendix S3), by running single surface optimisations and selecting the top ranked surface in correlated sets (according to Akaike's Information Criterion corrected for small sample size; AICc) (Akaike, 1974). We included IBD (where each pixel in the resistance surface is given a value of 1) and a null model (intercept only) in the model selection, which is built into the *ResistanceGA* package. Surfaces that ranked lower than, or within 2 AICc of the IBD or null models in the single surface model selection described above were excluded from multi‐surface optimisation, as they performed no better at modeling functional connectivity than the alternate or null hypotheses (Burnham & Anderson, 2002). Next, we performed multi‐surface optimisation using the “all_comb” function in *ResistanceGA* on a maximum of four combined surfaces. We conducted 1000 bootstrap iterations across random subsets of 75% of the total data to calculate the percentage of iterations where surfaces were ranked as the top model (similar to model weight) (Burnham & Anderson, 2002). This provides an indication of the level of support for each surface and whether particular sample subsets are disproportionately influencing model results (e.g., samples collected at different time points). Model performance was assessed by visualizing residuals based on a simulation approach using the *DHARMa* R package (Hartig, 2022).

## 3 RESULTS

### 3.1 Genetic data sets

After filtering, the final SNP data sets included 180 *N. timealeyi* samples genotyped at 4272 loci, 80 *P. chapmani* samples at 5049 loci, and 173 *P. hermannsburgensis* samples at 3844 loci. Filtering for HWE further reduced the number of loci to 3907 (*Nt*), 4925 (*Pc*) and 3638 (*Ph*). Microsatellite data sets included the same individuals to exactly match the individuals from the SNP data sets.

### 3.2 Isolation‐by‐barrier: genetic clustering

**FIGURE 2:**
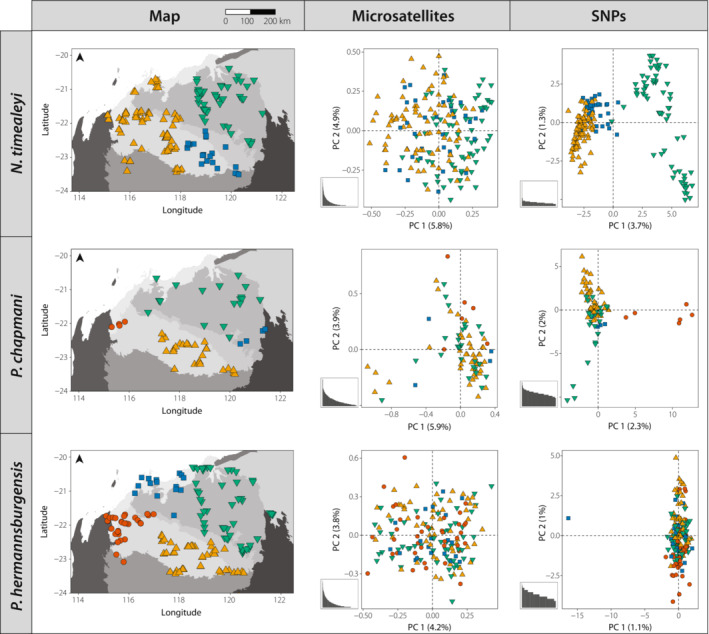
Maps indicating sample locations across the Pilbara for each of the three species (grey colours delineate IBRA subregions), and PCoA results comparing the first two axes for microsatellite versus SNP data sets (colours/shapes correspond to sample locations). Bar charts represent eigenvalues for all PCoA axes.

**FIGURE 3:**
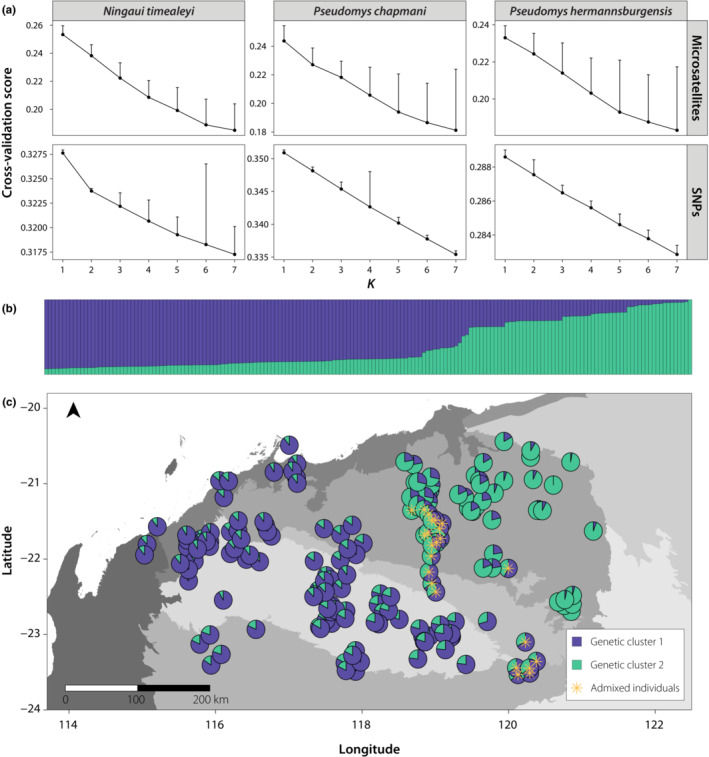
Cross‐entropy plots for *tess3r* analysis across marker types and species. Plots display root mean‐squared error predictions for a fraction of masked genotypes compared with true masked values (smaller values indicate better runs). The best value for *K* was chosen based on a change in slope/plateaux in the cross‐validation curve (a); *tess3r* results for *N. timealeyi* SNP data set for *K* = 2, where each bar represents an individual's ancestry proportions (b), and (c) individual ancestry proportions are displayed in pie charts. Yellow asterisks represent admixed samples that did not assign to a genetic cluster, based on an ancestry proportion threshold of ≥ 0.7.

We found no indication of temporal genetic structuring in our data sets (Appendix S4). There was no evidence for IBB within the rodent species using both marker types, while for *N. timealeyi*, SNPs resolved two genetic clusters that were not detected when using microsatellite markers (Figures 2 and 3). Although the first two principal coordinate axes of the PCoA explained more variation when using microsatellites compared to SNPs for all species (Microsatellites: *Nt* = 10.7%, *Pc* = 9.8%, *Ph* = 8%; SNPs: *Nt* = 5%, *Pc* = 4.3%, *Ph* = 2.1%; Figure 2), SNPs consistently outperformed microsatellites at revealing patterns of population genetic structure. When using SNPs, the PCoA delineated two main groups for *N. timealeyi*; one in the north‐east Chichester and the other including individuals in the western and south‐eastern Pilbara (Figure 2). These two population groupings were supported by the *tess3r* analysis of the SNP data set, with two ancestral populations identified (*K* = 2), although with admixture between the two clusters (*K* = 2; Figure 3b,c). In contrast, both the PCoA and *tess3r* analysis for *N. timealeyi* based on the microsatellites lacked support for population genetic structure (*K* = 1), showing no obvious plateau or change in slope of the cross‐entropy criterion for increasing values of *K* (Figure 3a).

Patterns of population genetic structure at SNP loci were more subtle for both *Pseudomys* species. The PCoA showed some evidence for potential western and north–south groupings for *P. chapmani*, while the PCoA for *P. hermannsburgensis* separated one individual on Enderby Island from Pilbara mainland individuals (Figure 2). Although this sample was also collected at the earliest timepoint (1988), we took this to represent genetic differentiation between island and mainland individuals given the lack of temporal clustering in the rest of the data set (Appendix S4). As with *N. timealeyi*, these same patterns were not resolved when using microsatellites (Figure 2). Furthermore, *tess3r* analysis did not detect population genetic structure at either SNPs or microsatellite markers, suggesting the Pilbara represents one genetic cluster (*K* = 1) for both *Pseudomys* species (Figure 3a).

### 3.3 Isolation‐by‐distance and summary statistics

Mantel tests and summary statistics were calculated across the total data set, as well as within the two genetic clusters (or “populations”; *K* = 2) for the *N. timealeyi* SNP data set identified in IBB analyses, excluding the 23 admixed individuals that did not assign to either population. For the *Pseudomys* SNP data sets, and the microsatellite data sets for all three species, analyses included all individuals as a single genetic population (*K* = 1).

**FIGURE 4:**
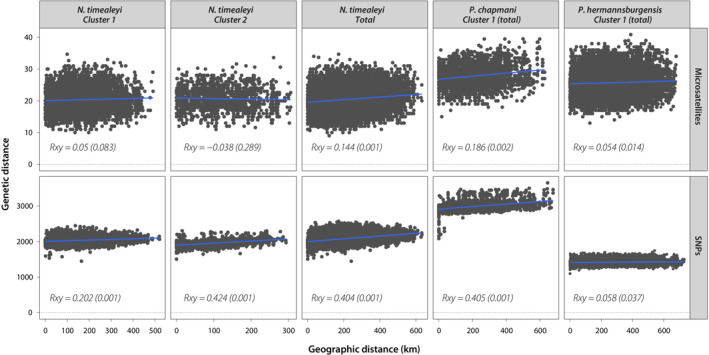
Mantel plots for each genetic cluster (or “population”) across marker types and species, where *Rxy* is the Mantel correlation coefficient and *p* indicates the significance using 999 permutations. Note that although only one genetic cluster was detected for *N. timealeyi* using the microsatellite data set, here we present the results for two clusters to match those identified using SNPs (for comparison), as well as for the total data set (i.e., where admixed individuals have not been removed).

We detected significant IBD (*p* < .05) within almost all populations identified in IBB analysis for each marker type for all three species (except for *N. timealeyi* cluster 1; Figure 4). However, while the weak positive relationship between genetic and geographic distance was consistent between marker types for *P. hermannsburgensis* (microsatellites: *Rxy* = 0.054, *p* = .014; SNPs: *Rxy* = 0.058, *p* = .037), the magnitude differed by a factor of approximately 1.5–3 between marker types for *N. timealeyi* (microsatellites: *Rxy* = 0.144, *p* = .001; SNPs: *cluster 1* – *Rxy* = 0.202, *p* = .001, *cluster 2* – *Rxy* = 0.424, *p* = .001) and *P. chapmani* (microsatellites: *Rxy* = 0.186, *p* = .002; SNPs: *Rxy* = 0.405; *p* = .001), suggesting SNPs were better able to resolve patterns of IBD (Figure 4). Furthermore, for the *N. timealeyi* SNP data set, IBD was stronger in cluster 2 than when IBD was performed across the total data set (Figure 4; *cluster 2* – *Rxy* = 0.424, *p* = .001, *total* – *Rxy* = 0.404, *p* = .001), potentially providing additional evidence for IBB.

Across both marker types, *P. chapmani* showed the highest heterozygosity (microsatellites: *H*_o_ = 0.775 ± 0.038, *H*_e_ = 0.878 ± 0.032; SNPs: *H*_o_ = 0.260 ± 0.002, *H*_e_ = 0.280 ± 0.002), followed by the other two species (*Nt* – microsatellites: *H*_o_ = 0.741 ± 0.042, *H*_e_ = 0.794 ± 0.034; SNPs: *H*_o_ = 0.215 ± 0.002 [cluster1], 0.208 ± 0.003 [cluster 2], *H*_e_ = 0.236 ± 0.002 [cluster 1], 0.227 ± 0.003 [cluster 2]; *Ph* – microsatellites: *H*_o_ = 0.756 ± 0.032, *H*_e_ = 0.834 ± 0.035; SNPs: *H*_o_ = 0.160 ± 0.002, *H*_e_ = 0.177 ± 0.002). Where population genetic structure was detected, genetic differentiation was low (*Nt* SNP data set: *F*_ST_ = 0.022 ± 0.001; Table 2).

**TABLE 2:** Mean population genetic summary statistics (±SE) for each genetic cluster (or “population”) across marker types and species.

	*Cluster*	*N*	*N_a_*	*H*_o_	*H_e_*	*F*
Microsatellites						
*N. timealeyi*	1	180 (12)	16.250 ± 2.180	0.741 ± 0.042	0.794 ± 0.034	0.073 ± 0.015
*P. chapmani*	1	80 (14)	20.000 ± 2.290	0.775 ± 0.038	0.878 ± 0.032	0.118 ± 0.026
*P. hermannsburgensis*	1	170 (14)	19.000 ± 1.980	0.756 ± 0.032	0.834 ± 0.035	0.090 ± 0.022
SNPs						
*N. timealeyi*	1	106 (3907)	1.990 ± 0.002	0.215 ± 0.002	0.236 ± 0.002	0.082 ± 0.002
	2	51 (3907)	1.940 ± 0.004	0.208 ± 0.003	0.227 ± 0.003	0.071 ± 0.003
*F*_IS_	1 vs. 2	0.080 ± 0.002				
*F*_ST_	1 vs. 2	0.022 ± 0.001				
*P. chapmani*	1	80 (4925)	2.000 ± 0.000	0.260 ± 0.002	0.280 ± 0.002	0.066 ± 0.002
*P. hermannsburgensis*	1	170 (3638)	2.000 ± 0.000	0.160 ± 0.002	0.177 ± 0.002	0.093 ± 0.002

*Note*: *N* = sample size (number of loci in parentheses), note that 23 *N. timealeyi* admixed individuals (SNPs only) and 3 *P. hermannsburgensis* individuals located on islands (microsatellites and SNPs) were removed from these analyses.

Abbreviations: *F*, fixation index; *H*_e_, expected heterozygosity; *H*_o_, observed heterozygosity; *N*_a_, number of alleles.

### 3.4 Isolation‐by‐resistance

**FIGURE 5:**
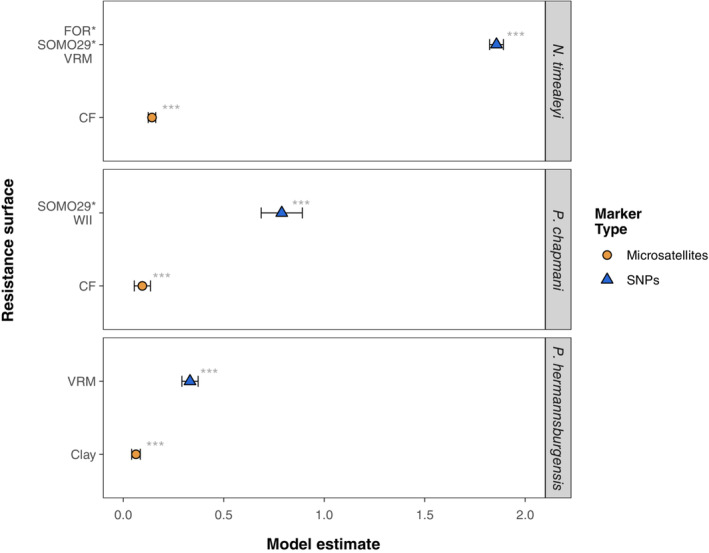
Forest plot displaying MLPE model estimates, 95% confidence intervals and significance (asterisks) for the three different species. Models were fit with optimised single/composite resistance surfaces as predictor variables and Euclidean genetic distance as the response variable and built using microsatellite (orange circles) versus SNP datasets (blue triangles).

The number of variables retained following single‐surface optimisation varied from one to eight (Table 1), with microsatellite‐based model selection consistently resulting in fewer variables being retained than for SNPs (i.e., variables performed worse than or equivalent to IBD or the null model). For the remaining variables, multi‐surface optimisation generated single and composite resistance surfaces that performed better in the model selection than the null model and IBD, for all three species across both marker types. However, SNP data sets better differentiated between the best models (models ≤2 ΔAICc from the top‐ranked model) and both IBD and null models, with ΔAICc for SNP IBD and null models 8 to 58 times greater (i.e., further from the top model) than for those using microsatellites (Table 3). The strength of the relationship between landscape resistance and genetic distance increased for all species when using SNP markers compared to microsatellites, although was most notable for *N. timealeyi* (model estimates for microsatellites vs. SNPs: *Nt* = 0.14 vs. 1.86; *Pc* = 0.09 vs. 0.79; *Ph* = 0.06 vs. 0.33; Figure 5). Furthermore, SNPs appeared to provide additional power to detect more subtle environmental associations between landscape elements and connectivity. Model selection based on SNPs for both *N. timealeyi* and *P. chapmani* ranked composite surfaces as the best performing models, while single surfaces consistently ranked best across microsatellite models.

**TABLE 3:** MLPE model selection across all species and marker types, for models performing within 2 ΔAICc of the top‐ranked model, as well as the isolation‐by‐distance (IBD) and null models for comparison (full model summaries and diagnostic plots can be found in Appendices S5–S7).

Species (marker)	Surface	ΔAICc	AICc weight	$R_{m}^{2}$	$R_{c}^{2}$	LL	Avg. rank	% Top
*Nt* (SNP)	FOR*SOMO29*VRM	0	0.66	.63	.93	−7806.88	2.80	45.4
	SOMO29*SPIN*VRM	1.35	0.34	.63	.93	−7807.56	2.80	35.5
	IBD	1569.69	0	.42	.70	−8600.62	9.36	0
	Null	6816.77	0	0	.28	−11,225.19	NA	NA
*Nt* (MSAT)	CF	0	0.97	.11	.39	−3015.11	1.04	97.2
	IBD	27.16	0	.02	.33	−3030.80	3.46	1.8
	Null	213.12	0	0	.32	−3124.82	NA	NA
*Pc* (SNP)	SOMO29*WII	0	0.74	.61	.79	−928.52	5.30	44.4
	IBD	49.88	0	.07	.63	−959.63	29.89	3
	Null	184.47	0	0	.60	−1028.02	NA	NA
*Pc* (MSAT)	CF	0	0.93	.06	.50	−297.37	1	100
	IBD	5.32	0.07	.01	.47	−302.35	2	0
	Null	14.45	0	0	.47	−307.99	NA	NA
*Ph* (SNP)	VRM	0	0.79	.21	.76	−3537.42	1.44	92.6
	IBD	85.61	0	.01	.66	−3582.34	83.14	0
	Null	246.99	0	0	.65	−3664.06	NA	NA
*Ph* (MSAT)	Clay	0	0.80	.03	.41	−2345.27	1.35	79.3
	IBD	9.61	0.01	0	.40	−2352.19	3.34	6.8
	Null	27.29	0	0	.40	−2362.06	NA	NA

*Note*: AICc weight, Akaike weight indicating the relative likelihood of each model; Avg. Rank, average model ranking over 1000 bootstrap iterations; LL, log‐likelihood; MSAT, Microsatellites; *Nt*, *Ningaui timealeyi*; *Pc*, *Pseudomys chapmani*; *Ph*, *Pseudomys hermannsburgensis*; $R_{c}^{2}$, conditional *R* ^2^; $R_{m}^{2}$, marginal *R* ^2^; SNP, single nucleotide polymorphisms; ΔAICc, ranking of Akaike Information Criterion corrected for small sample size in relation to best performing model; % Top, percentage of 1000 bootstrap iterations.

While model selection for each marker type did not identify the same specific surfaces in the best performing models, landscape elements were somewhat similar. For the *N. timealeyi* SNP model selection, there was support for two models using composite surfaces, including soil moisture (SOMO29), terrain ruggedness (VRM) and a vegetation element (*top model* – persistent forest cover [FOR], weight = 0.663, AICc = 15,635.646, marginal *R* ^2^ = .634; *second model* – spinifex density [SPIN], weight = 0.0.337, AICc = 15,636.998, marginal *R* ^2^ = .633; ΔAICc for the third‐best model = 72.23; Table 3; Appendices S5–S7). Together, these models ranked first in 81% of bootstrap iterations (Table 3; Appendix S8). In both cases, the optimized resistance surface primarily represented ruggedness, which made the greatest contribution to the composite surface (*top model* = 80.68%; *second model* = 79.75%), followed by soil moisture (*top model* = 18.29%; *second model* = 19.85%), and a minimal contribution by the vegetation element (*top model* [FOR] = 1%; *second model* [SPIN] = 0.39%). For both models, landscape resistance decreased with increasing ruggedness and soil moisture, and increased as the specific vegetation cover/density (FOR vs. SPIN) increased (Appendix S9). In comparison, the top‐ranked model for the microsatellite data set (weight = 0.965, AICc = 6038.543, marginal *R* ^2^ = .108, ΔAICc for the second‐best model = 6.63; Table 3; Appendices S5–S7) included a single surface of coarse fragments (CF) which was highly correlated with VRM (Appendix S3). This model ranked first in 97.2% of bootstrap iterations and revealed a negative relationship between landscape resistance and coarse fragments (Table 3; Appendices S8 and S9). Thus, regardless of whether SNPs or microsatellites were used to generate genetic response data, models revealed that increasing ruggedness and rockiness (coarse fragments) facilitate landscape connectivity, although the magnitude of this effect was approximately 13 times greater when using SNPs (Figure 5; Appendix S6).

The *P. chapmani* SNP model selection provided moderate support for a top‐ranked model including a composite surface of soil moisture (SOMO29) and weathering intensity (WII; weight = 0.748, AICc = 1873.642, marginal *R* ^2^ = .612; ΔAICc for the second‐best model = 2.172; Table 3; Appendices S5–S7), which ranked first in 44.4% of bootstrap iterations (Table 3; Appendix S8). Soil moisture contributed most to the composite surface (71.26%), with landscape resistance decreasing as soil moisture increased (Appendix S9). Weathering intensity made a 28.74% contribution to the composite surface, with landscape resistance increasing with increased weathering (Appendix S9). In contrast, the microsatellite model selection provided moderate support for a top‐ranked model including coarse fragments (CF) only (weight = 0.581, AICc = 602.832, marginal *R* ^2^ = .055, ΔAICc for the second‐best model = 5.241; Table 3; Appendices S5–S7). This model ranked first in 100% of bootstrap iterations, with landscape resistance decreasing as the percentage of coarse fragments increased (Table 3; Appendices S8 and S9). While analyses based on SNP versus microsatellite genetic response data selected different surfaces, both showed that either increased rocky outcrops (lower weathering) or increased rocky substrate (coarse fragments) facilitate landscape connectivity, although SNPs also found increased soil moisture was important. The magnitude of this effect was approximately nine times greater when using SNPs compared to microsatellite markers.

Both SNP and microsatellite model selection for *P. hermannsburgensis* supported a top model including a single surface of terrain ruggedness (VRM; weight = 0.785, AICc = 7091.639, marginal *R* ^2^ = .209; ΔAICc for the second‐best model = 6.611; Table 3; Appendices S5–S7), or clay (weight = 0.798, AICc = 4698.857, marginal *R* ^2^ = .025; ΔAICc for the second‐best model = 4.582; Table 3; Appendices S5–S7), respectively. The best supported SNP model ranked first in 92.6% of bootstrap iterations, compared to 79.3% for the microsatellite model (Table 3; Appendix S8). In both cases, landscape resistance increased as either ruggedness or clay content increased (Appendix S9). Although the SNP and microsatellite genetic response data selected different surfaces, both found that non‐sandy substrates (rocky/rugged terrain and clay) increased landscape resistance, with the magnitude of this effect approximately 5.5 times greater when using the SNP dataset (Figure 5).

## 4 DISCUSSION

Increasingly the field of conservation genetics is transitioning to the use of genomic data to understand patterns of connectivity in wild populations. While SNP markers appear to provide increased resolution for detecting population genetic structure, so far there has been a lack of studies to investigate the performance of SNPs relative to microsatellites for empirical landscape genetic analyses. Here we provide a case study using these two marker types to evaluate three explicit landscape genetic hypotheses (IBD, IBB and IBR) in a suite of small arid‐zone mammals possessing high dispersal capacities. In general, SNP markers provided additional resolution in detecting subtle genetic structuring in IBB analyses, particularly for the dasyurid, and resulted in stronger patterns of IBD and IBR in both rodent and dasyurid species. However, the large dispersal capacity and the dynamic nature of arid landscapes present specific challenges for IBR analyses, which we discuss below.

### 4.1 Isolation‐by‐barrier: SNPs versus microsatellites

Here, using SNP data, we identified genetic structure in the dasyurid species, *N. timealeyi*, that was not detected with microsatellite data using the same individuals (in this study and in Levy et al., 2019). Our results add to a growing body of work suggesting SNPs provide higher resolution for population genetic analyses compared to microsatellites, particularly in species with weak population structure (Jeffries et al., 2016; Puckett & Eggert, 2016; Viengkone et al., 2016). Similar to our results, Camacho‐Sanchez et al. (2020) compared both marker types in two amphibians, concluding that SNP data sets with large numbers of loci are more reliable at identifying population genetic structure at large spatial scales (~500,000 square kilometers). Furthermore, a synthesis of studies using both marker types showed that SNPs were either equivalent or outperformed microsatellites at detecting population genetic structure, suggesting this pattern is broadly representative, rather than study or context specific (Sunde et al., 2020).

Single nucleotide polymorphism markers indicated two genetically distinct groups in *N. timealeyi*, although given the large degree of admixture, these clusters may be better described as representing a geographic cline. These clusters are somewhat aligned with the Abydos Plain and Oakover Valley which are known to have distinct vegetation assemblages (McKenzie et al., 2009). Phylogenetic studies on several reptile genera (geckos) revealed similar genetic division (Pepper et al., 2013), suggesting this split may represent an ecological transition zone and could be present across multiple taxa. Alternatively, given that SNPs have a slower mutation rate than microsatellites (Morin et al., 2004), this pattern may also reflect the ancestral genetic signature present prior to aridification in the mid‐late Pleistocene (approximately 15–25 kya). This period saw rivers transition from perennial to ephemeral flows and the expansion of drought resistant flora (Byrne et al., 2008). Thus, a third scenario of reconnection of previously separated refugial populations may also explain the substantial admixture and low differentiation between these genetic clusters. In fact, Umbrello et al. (2020) found evidence of population expansion in six small dasyurids across the Pilbara since the mid‐late Pleistocene and the Last Glacial Maximum (LGM) and proposed that this followed the increased availability of arid habitat. Refugial separation prior to population expansion after the LGM has also been detected in sea spiders (Soler‐Membrives et al., 2017), mussels (Cunha et al., 2011) and ants (Xun et al., 2016), with weak differentiation reflecting the loss of refugial genetic structure over time due to high dispersal capacities.

In contrast, we were not able to detect evidence for population genetic structuring within the Pilbara landscape for the two native *Pseudomys* species across both SNP and microsatellite data sets, suggesting a lack of landscape barriers to dispersal. However, SNPs were still able to resolve some subtle patterns not detected with microsatellites (e.g., PCoA groupings of the *P. hermannsburgensis* island individual and the subtle western and north–south groupings for *P. chapmani*). The weak clusters detected in the SNP *P. chapmani* data set may reflect the accumulation of positive spatial‐genetic structure driven by the sociality of the species (i.e., family groups within pebble mounds) (Firman et al., 2019; Ford & Johnson, 2007). However, this structure was too weak to be detected with our sampling strategy (spatially dispersed individuals) and the clustering analysis. Several other genetic studies in rodents also find low population structure even in the presence of major landscape barriers or considerable landscape heterogeneity (Gauffre et al., 2008; Vega et al., 2007). This is likely because irruptive boom‐bust population dynamics obscure any signals of population structure.

### 4.2 Isolation‐by‐distance and isolation‐by‐resistance: SNPs versus microsatellites

Few studies have evaluated the ability of microsatellites versus SNPs to detect IBD and we are not aware of any that have compared results between marker types for identifying IBR. In a comparative study using RADseq SNPs and microsatellites, Jeffries et al. (2016) identified a stronger signature of IBD from the SNP data than from microsatellite data sets, suggesting this may be due to the mutational processes of the markers. Similarly, we detected significant IBD for all species and marker types, and this signature was stronger when using SNPs in some cases. For example, while both marker types showed weak IBD for *P. hermannsburgensis*, the magnitude of the correlation increased when using SNPs for *N. timealeyi* and *P. chapmani*. Perhaps the increased power provided by more loci, coupled with the slower mutation rate of SNPs was able to resolve this subtle pattern, suggesting that these species have more constrained dispersal capacities than *P. hermannsburgensis*. For IBR, we found that SNPs tended to resolve more complex resistance surfaces (i.e., composite surfaces of multiple environmental layers) than microsatellites, potentially reflecting the increased power of large SNP panels to detect subtle and complex patterns of functional connectivity. SNP models also revealed a stronger effect of landscape resistance on genetic distance and tended to better differentiate between the top models and the alternate IBD hypothesis, adding to the body of evidence arguing that SNPs provide better resolution for questions that require individual‐level genetic information, such as relatedness, individual identification and fine‐scale genetic structure (Sunde et al., 2020).

High resolution spatial data in combination with sophisticated landscape resistance modeling revealed additional detail on the landscape characteristics influencing functional connectivity in our target species than detected in Levy et al. (2019), and the identified drivers of connectivity were largely consistent between marker types. When directly compared via model selection, we found that IBR hypotheses outcompeted the alternate hypothesis of IBD in all cases. However, these results should be interpreted carefully since the high power inherent to large pairwise data sets, combined with correlations between competing IBD and IBR models, can result in low model selection accuracy (Shirk et al., 2018). The large dispersal capacity and boom‐bust dynamics of arid‐zone mammals makes this issue particularly pertinent for our study, given that functional connectivity would likely be approaching IBD for many species. Given these issues, we attempted to reduce model selection error by following best practice recommendations, including using linear mixed‐effects models fit with MLPE and by transforming resistance surfaces to satisfy assumptions of linearity, as this approach has been shown to outperform other regression methods (Shirk et al., 2018). We also used individual‐genetic distance, which is sensitive to contemporary genetic structure (Shirk et al., 2017), since population‐level analyses are less representative of species that are continuously distributed. Finally, we used a data‐driven approach to parameterising resistance surfaces based on spatial layers that are biologically plausible, to tease apart competing hypotheses and determine the most likely characteristics contributing to landscape resistance.

Simulations can be used to undertake power analyses and evaluate findings (i.e., to determine whether marker panels have the power to detect genetic patterns in specific systems or scenarios). Simulation tools such as CDPOP (Landguth & Cushman, 2010) and HexSim (Schumaker & Brookes, 2018) have contributed greatly to this goal, however, it can be difficult to parameterise simulations, particularly in relatively understudied systems such as the arid landscape presented here, due to complex and unknown species' demography. In particular, it is not yet feasible to simulate landscapes where genetic structure plays out over such a vast scale and there is still much work to be done to develop landscape genetic tools to help us understand the interplay between boom‐bust dynamics, temporally and spatially dynamic refuges (common features of arid landscapes), and dispersal. Further research on arid systems can provide greater mechanistic understanding of these patterns and processes. Although we cannot make a quantitative and comparative power analysis without further simulation testing in our study, we can interpret our results alongside the literature to determine biological plausibility and provide useful ecological insights for conservation management.

Using this approach across both marker types, we found that increasing ruggedness or rockiness facilitated landscape connectivity for *N. timealeyi*. This species is a habitat generalist, weakly associated with clay substrates and also found on rugged terrain (Gibson & McKenzie, 2009), highlighting the fact that the habitat type required for dispersal can be a subset of, or decoupled from, the type of habitat required for establishing territories. For example, Keeley et al. (2017) found that Kinkajou (*Potos flavus*), an arboreal mammal, will readily cross non‐forested habitat during dispersal and mating movements despite having home ranges tightly linked to forested areas. In the case of *N. timealeyi*, the increased complexity of rocky, rugged habitat and dense vegetation (also identified as having a positive effect on connectivity) may provide protection from predators during dispersal (Table 1; Moore et al., 2019). Additionally, SNP IBR models indicated that higher soil moisture increased connectivity, likely reflecting mesic conditions more conducive to dispersal in this arid landscape. In contrast, both *Pseudomys* species showed a comparatively weaker effect of landscape resistance on functional connectivity, again likely indicative of the irruptive population dynamics present in rodents compared to dasyurids. Results for *P. hermannsburgensis* were consistent with previous research showing an association with sandy soils (Gibson & McKenzie, 2009), as our top models included a negative effect of non‐sandy substrates on landscape connectivity. Interestingly, while *N. timealeyi* and *P. hermannsburgensis* both showed top models that were orders of magnitude higher than the IBD (and null) models, this difference was slightly less pronounced for *P. chapmani* (particularly using the microsatellite data set), suggesting that functional connectivity is approaching IBD for this species. Still, our results indicated that increased rocky outcrops (lower weathering—SNPs) or increased rocky substrate (coarse fragments—microsatellites), and increased soil moisture (SNPs) facilitated landscape connectivity. This is biologically plausible since this species is a rocky habitat specialist, although our results suggest that due to the fragmented nature of rocky habitat in the Pilbara (Ford & Johnson, 2007), dispersal must often occur between these patches, a pattern also seen in other species found in rocky habitat in the Pilbara (Shaw et al., 2022).

## 5 CONCLUSIONS

There has been a rapid shift from microsatellite markers to SNPs in the fields of conservation and population genetics, although studies suggest that the major benefit of SNPs is not inherently about the marker type, but the number used (Sunde et al., 2020). We found that SNPs resolved subtle genetic structuring not detected by microsatellites, stronger signatures of isolation‐by‐distance and isolation‐by‐resistance, and identified more complex resistance surfaces. While patterns of genetic structure were subtle, our study demonstrates that the use of SNPs, coupled with novel landscape genetics analyses, can provide new ecological insights into arid landscapes, although microsatellites were still able to identify similar (albeit more simplified) results.

Understanding subtle resistance patterns in highly permeable landscapes is not of obvious conservation concern (Shirk et al., 2018). However, the Pilbara is substantially impacted by competing land uses, including mining and pastoralism (and the cumulative impacts of habitat clearance and fragmentation), as well as climatic cycles that drive dynamic drought and fire regimes (Cramer et al., 2016, 2022; McKenzie et al., 2009). Even in large, panmictic populations, maintaining functional connectivity is crucial, as ongoing fragmentation can erode meta‐population health (Umbrello et al., 2022). Thus, knowledge of factors driving connectivity is crucial for supporting resilient populations of both threatened and non‐threatened species in Australia, and globally.

## AUTHOR CONTRIBUTIONS

**Ebony D. Skey:** Formal analysis (lead); investigation (lead); visualization (equal); writing – original draft (lead); writing – review and editing (equal). **Kym M. Ottewell:** Conceptualization (supporting); data curation (supporting); formal analysis (supporting); funding acquisition (lead); investigation (supporting); methodology (supporting); project administration (supporting); resources (equal); supervision (equal); writing – original draft (supporting); writing – review and editing (equal). **Peter B. Spencer:** Conceptualization (supporting); funding acquisition (supporting); investigation (supporting); methodology (supporting); project administration (lead); resources (equal); supervision (equal); writing – original draft (supporting); writing – review and editing (equal). **Robyn E. Shaw:** Conceptualization (lead); data curation (lead); formal analysis (lead); investigation (supporting); methodology (lead); supervision (lead); visualization (equal); writing – original draft (supporting); writing – review and editing (lead).

## BENEFIT‐SHARING STATEMENT

All data and R code are available from Github (https://github.com/RobynSh/LandGen_AridMammals) and have been archived in a Zenodo Digital Repository (Shaw, 2023).

This research is part of a collaborative project across academic, government and industry partners and addresses a priority concern by testing the methods being applied to the conservation of small mammals in the broader project. Benefits from this research accrue from the sharing of our data and results on public databases as described above.

## Supporting information


Appendix S1–S9
