# Supplementary material for: Empirical landscape genetic comparison of single nucleotide polymorphisms and microsatellites in three arid‐zone mammals with high dispersal capacity
Source: Ecol Evol. 2023 May 2;13(5):e10037. doi: 10.1002/ece3.10037 (PMC10154367; doi:10.1002/ece3.10037)
Supplement: Supplementary file 1 — Appendix S1–S9 [file ECE3-13-e10037-s001.docx]

**Supporting Information for manuscript entitled:**

*Empirical landscape genetic comparison of SNPs and microsatellites in three arid-zone mammals with high dispersal capacity*

**Authors:**

Ebony D Skey^1,2^, [ebony.skey@dbca.wa.gov.au](mailto:ebony.skey@dbca.wa.gov.au), ORCID: 0000-0002-9640-1276

Kym M Ottewell^2^, [kym.ottewell@dbca.wa.gov.au](mailto:kym.ottewell@dbca.wa.gov.au), ORCID: 0000-0002-4273-3224

Peter B Spencer^1^, [P.Spencer@murdoch.edu.au](mailto:P.Spencer@murdoch.edu.au), ORCID: 0000-0002-4938-2615

Robyn E Shaw^1,2,3^, [robyn.shaw@anu.edu.au](mailto:robyn.shaw@anu.edu.au), ORCID: 0000-0002-7899-1743

**Affiliations:**

^1^ Environmental & Conservation Sciences, Murdoch University, Perth, WA, Australia, 6150

^2^ Biodiversity and Conservation Science, Department of Biodiversity, Conservation and Attractions, Locked Bag 104, Bentley Delivery Centre, Kensington, Western Australia, 6152, Australia

^3^ Current address: Division of Ecology and Evolution, Research School of Biology, The Australian National University, Canberra, ACT, Australia, 2600

**Corresponding Author:** Robyn E Shaw: [robyn.shaw@anu.edu.au](mailto:robyn.shaw@anu.edu.au)

**Appendix S1. Detailed description of the SNP filtering process.**

We used a custom R script to carry out SNP filtering. This script followed (Shaw, 2022; Shaw et al., 2022), and allowed us to visualise our data and determine the following thresholds:

*Call rate (individual and loci) –* Individual missing data thresholds were chosen based on the number of SNPs retained if all individuals were filtered on a hypothetical 85% locus call rate, balancing the trade-off between number of samples, importance of lower quality samples and potential number of loci. Individual filters were implemented first, with a threshold of 0.5 (one individual removed), 0 (zero individuals removed) and 0.7 (two individuals removed) for *N. timealeyi, P. chapmani*, and *P. hermannsburgensis* respectively. To determine the call rate threshold across loci, we explored the relationship between call rate and population genetic summary statistics (heterozygosity estimates and F-statistics), under the assumption that data quality and biologically meaningful population genetic metrics should not be correlated. We chose thresholds that minimised this correlation, while retaining a large enough data set for adequate statistical power (*Nt* = 0.95, *Pc* = 0.96, *Ph* = 0.95).

*Read count –* We explored the raw read count data provided by DArT, and found that filtering on mean read count across individuals was equivalent to filtering on individual read count (i.e. read count per locus per individual). Therefore, read count filtering was based on mean read count across individuals. To inform our decision, we plotted the relationship between read count and genotype proportions, under the assumption that the called genotype should not be influenced by the number of reads. We chose minimum and maximum cut-offs based on where this relationship was stable/uncorrelated (*Nt =* between 20 and 100, *Pc* = between 15 and 25, *Ph =* between 20 and 40). For example, at lower read counts, heterozygotes tended to be under called, while SNP homozygotes were over called. At very high read counts, the genotypes became fixed for the reference or the SNP allele (indicating that these may represent paralogous regions).

*Repeatability average –* The repeatability filter threshold was chosen to exclude poor quality loci, while not filtering out SNPs with high levels of heterozygosity (as heterozygote genotype calls may vary between replicates due to slight variations in reference/SNP reads). For this reason, we used a threshold of 0.95 for all species.

*Minor allele frequency (MAF) –* We chose a MAF threshold that balanced the trade-off between including SNPs that represented sequencing error, versus removing true low frequency/private alleles. We used a minimum threshold which equated to each allele appearing at least twice in the data set, i.e., we removed singletons from the data set (*Nt* = 0.025, *Pc* = 0.05, *Ph* = 0.025). As the sample size for *P. chapmani* was smaller than for the other two species, this equated to a larger MAF threshold.

*Linkage disequilibrium –* We performed the LD filter last, as many non-independent SNPs are removed by the earlier filters. This means that performing the LD filter last maximises the number of quality loci that are retained. We first removed non-independent SNPs by randomly retaining one locus per sequence (and excluding other loci found in the same fragment). Next, we used *SNPRelate* version 1.20.1 to perform pairwise genotypic correlations within a sliding window of 500,000 base pairs, removing SNPs with a correlation of ≥0.5.

**Appendix S2. Raster layers.**

|  | **Description and Processing** | | **Plot** |
| --- | --- | --- | --- |
| **General** | All rasters were reprojected to UTM50S, cropped to the smallest raster extent (the light grey area), then resampled to the same pixel size and origin. Rasters were aggregated (by the mean value across aggregated pixels) to a 5 km resolution. Raster processing was carried out using the R package *raster* (Hijmans, 2020). | | 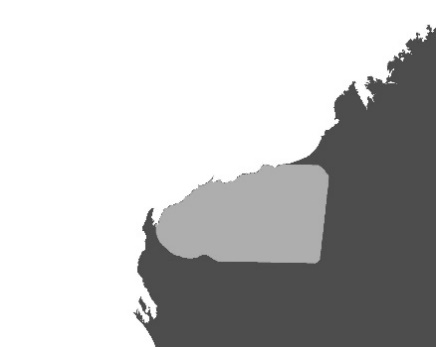  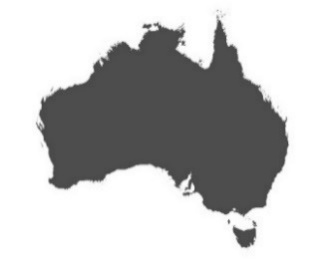 |
|  | **Raster layer** | **Description and Processing** | **Plot** |
| **Aridity Indices** | ADI  (Harwood et al., 2016) | Minimum of monthly aridity index (monthly precipitation/ monthly potential evaporation) – 30 year average centred on 1990 (Xu & Hutchinson, 2011). | 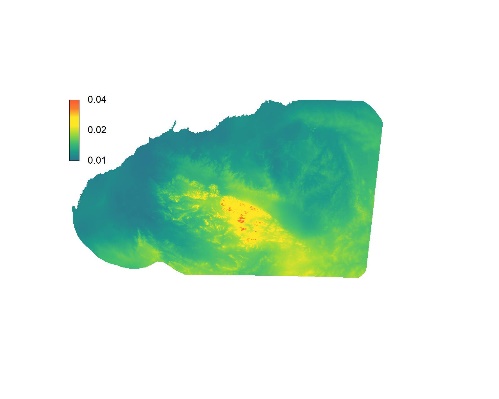 |
|  | ADM  (Harwood et al., 2016) | Mean annual aridity index (annual precipitation/ annual potential evaporation) – 30 year average centred on 1990 (Xu & Hutchinson, 2011). | 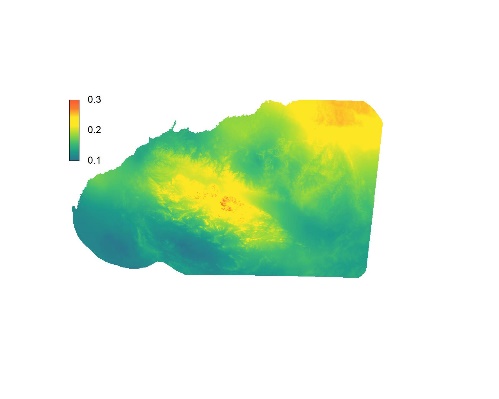 |
|  | ADX  (Harwood et al., 2016) | Maximum of monthly aridity index (monthly precipitation/ monthly potential evaporation) – 30 year average centred on 1990 (Xu & Hutchinson, 2011). | 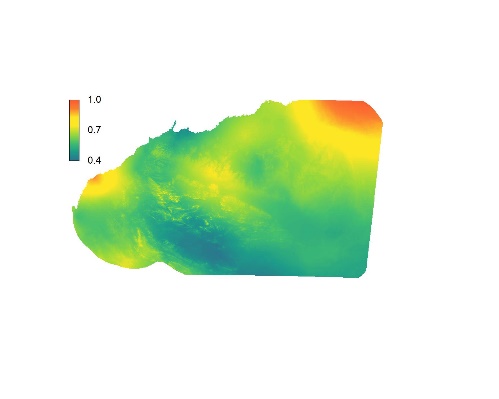 |
| **Relative Soil Moisture Indices** | SOMO29  (Harwood, 2019) | Highest period moisture index (relative soil moisture) – 30 year average centred on 1990. The maximum moisture index value for all weeks of the year (Xu & Hutchinson, 2011). | 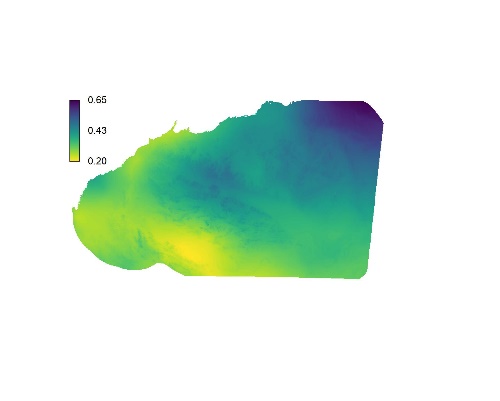 |
|  | SOMO30  (Harwood, 2019) | Minimum moisture index value (relative soil moisture) for all weeks – 30 year average centred on 1990. The minimum moisture index value for all weeks of the year (Xu & Hutchinson, 2011). | 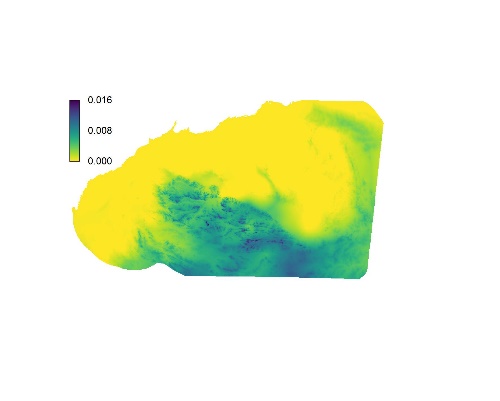 |
|  | SOMO 31  (Harwood, 2019) | Moisture index (relative soil moisture) seasonality (coefficient of variation) – 30 year average centred on 1990. The standard deviation of the weekly moisture index values expressed as a percentage of the mean of those values (Xu & Hutchinson, 2011). | 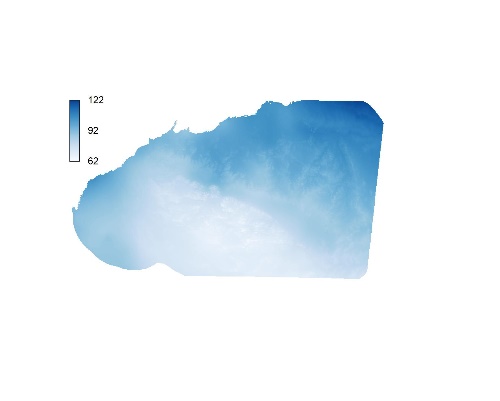 |
|  | SOMO 32  (Harwood, 2019) | Mean moisture index (relative soil moisture) of wettest quarter – 30 year average centred on 1990. The average moisture index over the quarter of the year with the highest average moisture index value (Xu & Hutchinson, 2011). | 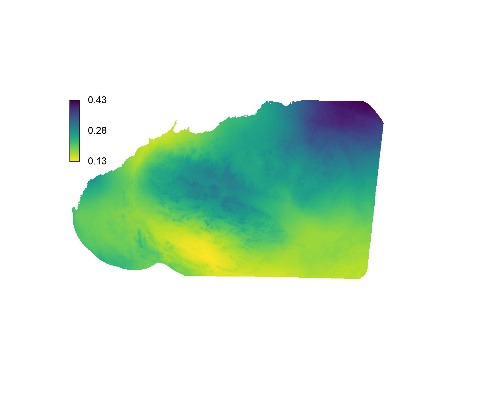 |
|  | SOMO 33  (Harwood, 2019) | Mean moisture index (relative soil moisture) of driest quarter – 30 year average centred on 1990. The average moisture index over the quarter of the year with the lowest average moisture index value (Xu & Hutchinson, 2011). | 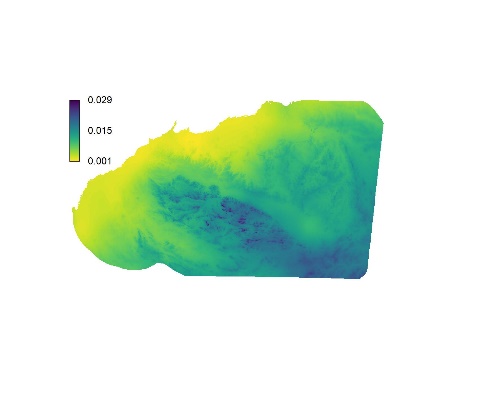 |
| **Substrate** | Clay  (Holmes, Griffen, & Odgers, 2014) | Layers representing the estimated value (%) of each soil type between 0-5 cm, 5-15 cm, 15-30 cm and 30-60 cm were downloaded using the R package *slga* (O’Brien, 2020). The mean percentage was then calculated across raster layers, under the assumption that a depth of 0 – 60 cm would have the greatest influence on animal movement and the ground level vegetation that provides protection from predation and food. | 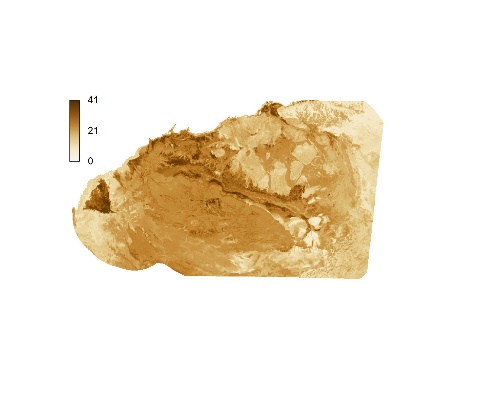 |
|  | Coarse Fragments (CF)  (Holmes et al., 2014) |  | 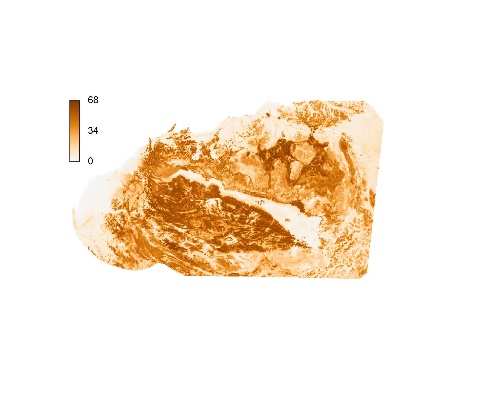 |
|  | Sand  (Holmes et al., 2014) |  | 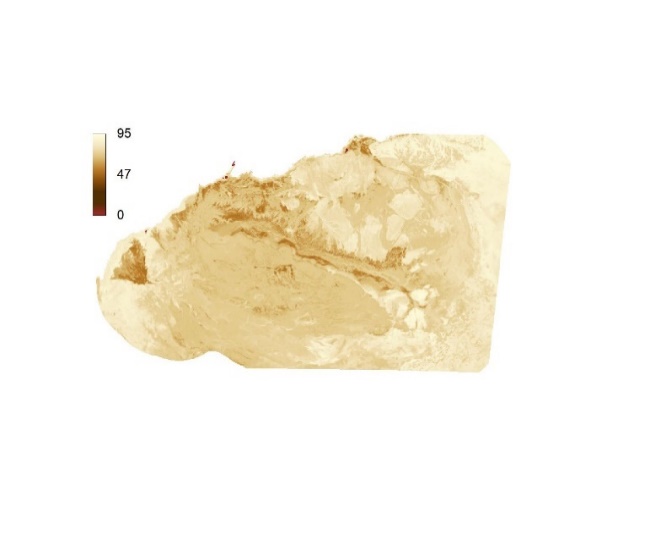 |
|  | Silt  (Holmes et al., 2014) |  | 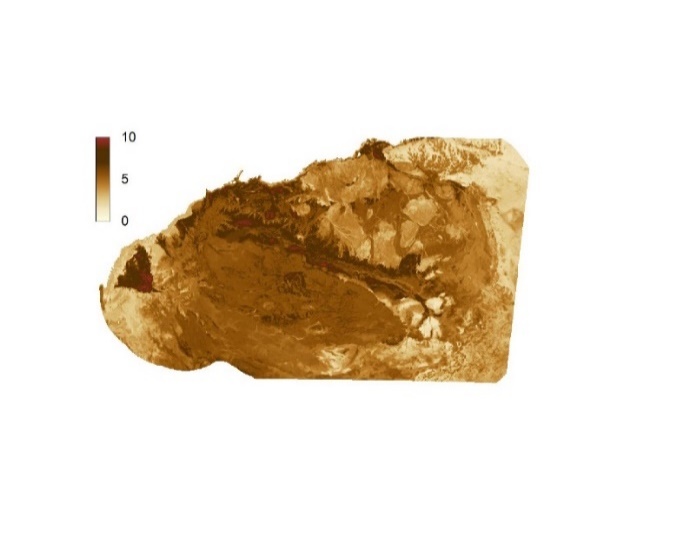 |
| **Topographic Features** | Weathering Intensity Index (WII)  (Wilford, 2012; Wilford & Roberts, 2019) | Weathering Intensity Index (WII). Developed using regression models based on airborne gamma-ray spectrometry imagery and the Shuttle Radar Topography Mission (SRTM) elevation data. This index can provide an indication of regolith properties, with 1 representing unweathered bedrock (where outcrops are common), through to 6 representing intensely weathered areas (where clays and sands are common). | 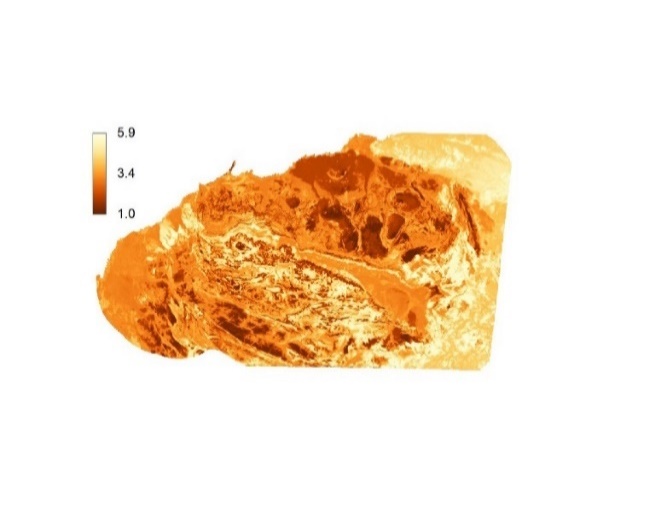 |
|  | Vector Ruggedness Measure (VRM) | A Vector Ruggedness Measure (VRM) was derived from the DEM (Gallant, Wilson, Dowling, Read, & Inskeep, 2011) using the R package *spatialEco* (Evans, 2020). VRM was chosen to represent terrain ruggedness, as it is less correlated with slope than other methods (Sappington, Longshore, & Thompson, 2007), allowing us to test terrain complexity, rather than slope. | 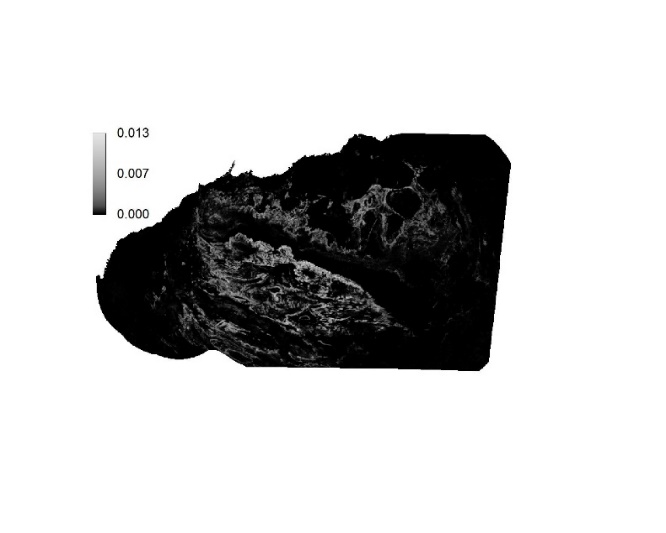 |
|  | Digital Elevation Model (DEM)  (Gallant et al., 2011) | Bare-earth Digital Elevation Model, adaptively smoothed to reduce random noise typically associated with the SRTM data in low relief areas. | 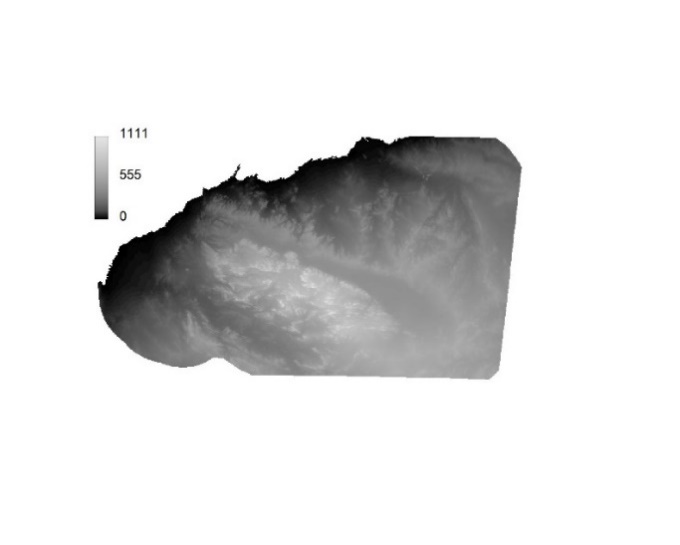 |
| **Distance to Water** | Distance to Water (WAT) | Euclidean distance (m) to natural perennial water (i.e., excluding artificial water points, and inland flats subject to inundation or flooding), derived from Landgate TGDB Hydrology (Landgate, 2012, 2017, 2019) in ArcGIS (ESRI, 2018). | 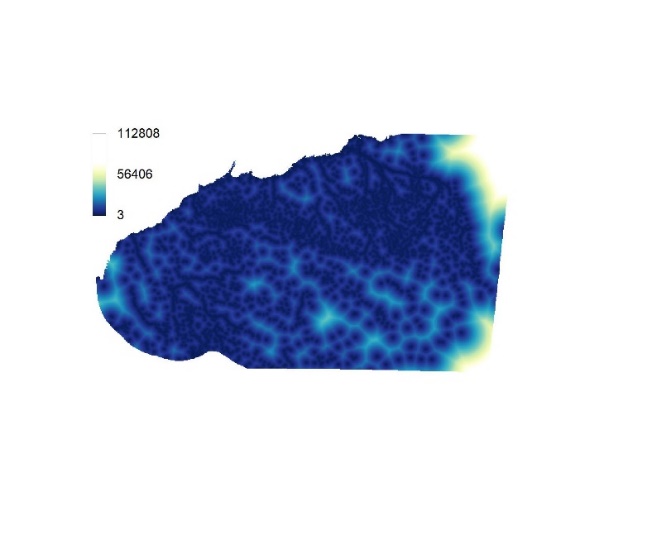 |
| **Vegetation** | Persistent Forest Cover (FOR) | Persistent forest cover. Derived using Land Monitor State-wide products (woody vegetation) (Furby, 2018; Furby, Wallace, & Caccetta, 2007). Frequency data from 23 annual woody layers between 1988 – 2018, including forest cover (>20% canopy cover with expected height at maturity >2m) and sparse woody vegetation cover (5-20% canopy cover). | 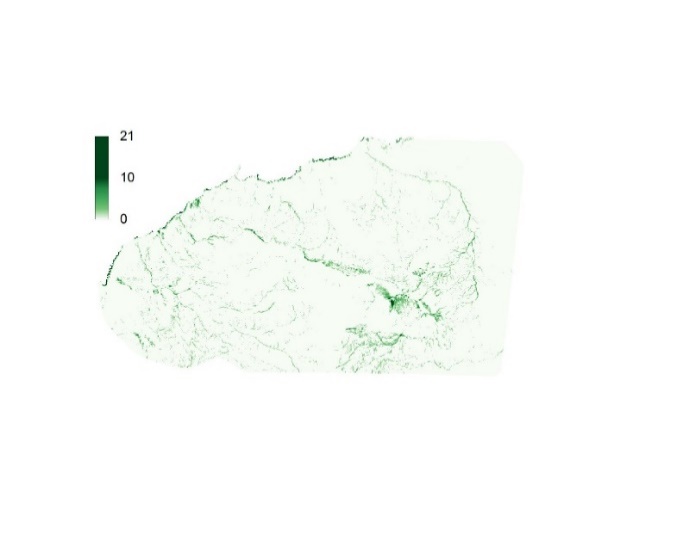 |
|  | Spinifex density Index (SPIN) | Spinifex density index (Rampant, Zdunic, & Burrows, 2019) derived using Landsat NBART (Li et al., 2012). A composite of decision rules were applied to determine the likeliest locations of spinifex dominated grasslands in the Pilbara region. | 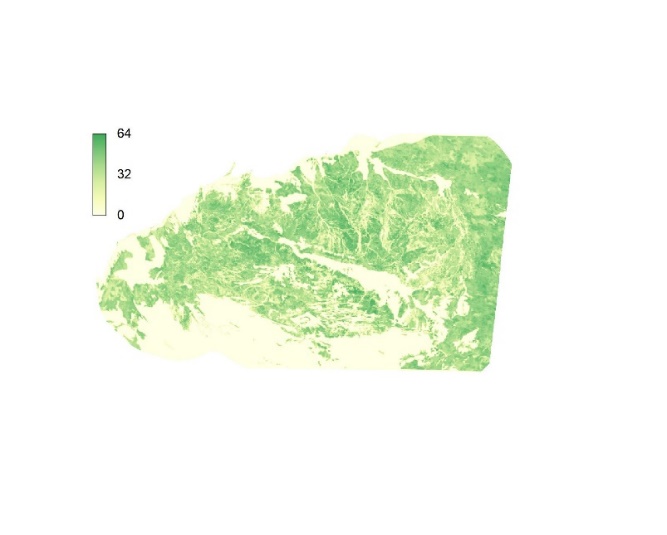 |
| **Fire** | Fire Frequency (FF) | Fire frequency between 2000 – 2008. Annual fire scar mapping was produced using equivalent methods to NAFI (North Australia and Rangelands Fire Information, 2019). | 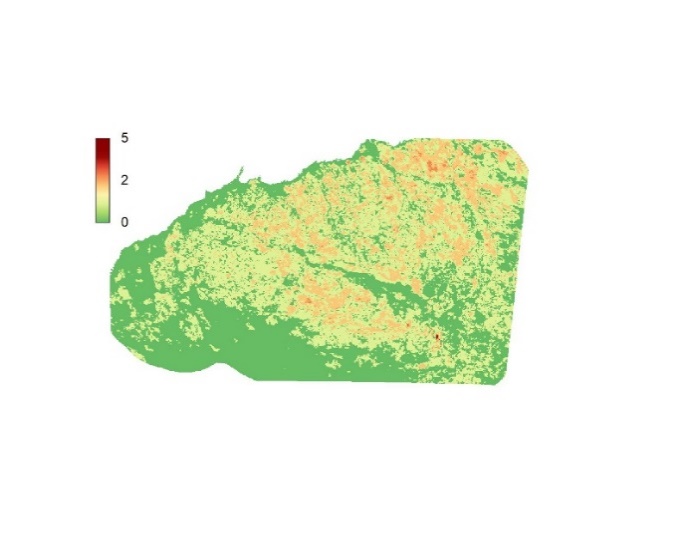 |

**Appendix S3. Raster correlations.**

Correlation matrix (Spearman’s r_s_) between rasters tested in IBR hypotheses, where CF = coarse fragments; FF = fire frequency; FOR = persistent forest cover; SOMO = soil moisture; SPIN = spinifex density index; VRM = Vector Ruggedness Measure; WAT = distance to water; WII = Weathering Intensity Index (WII).


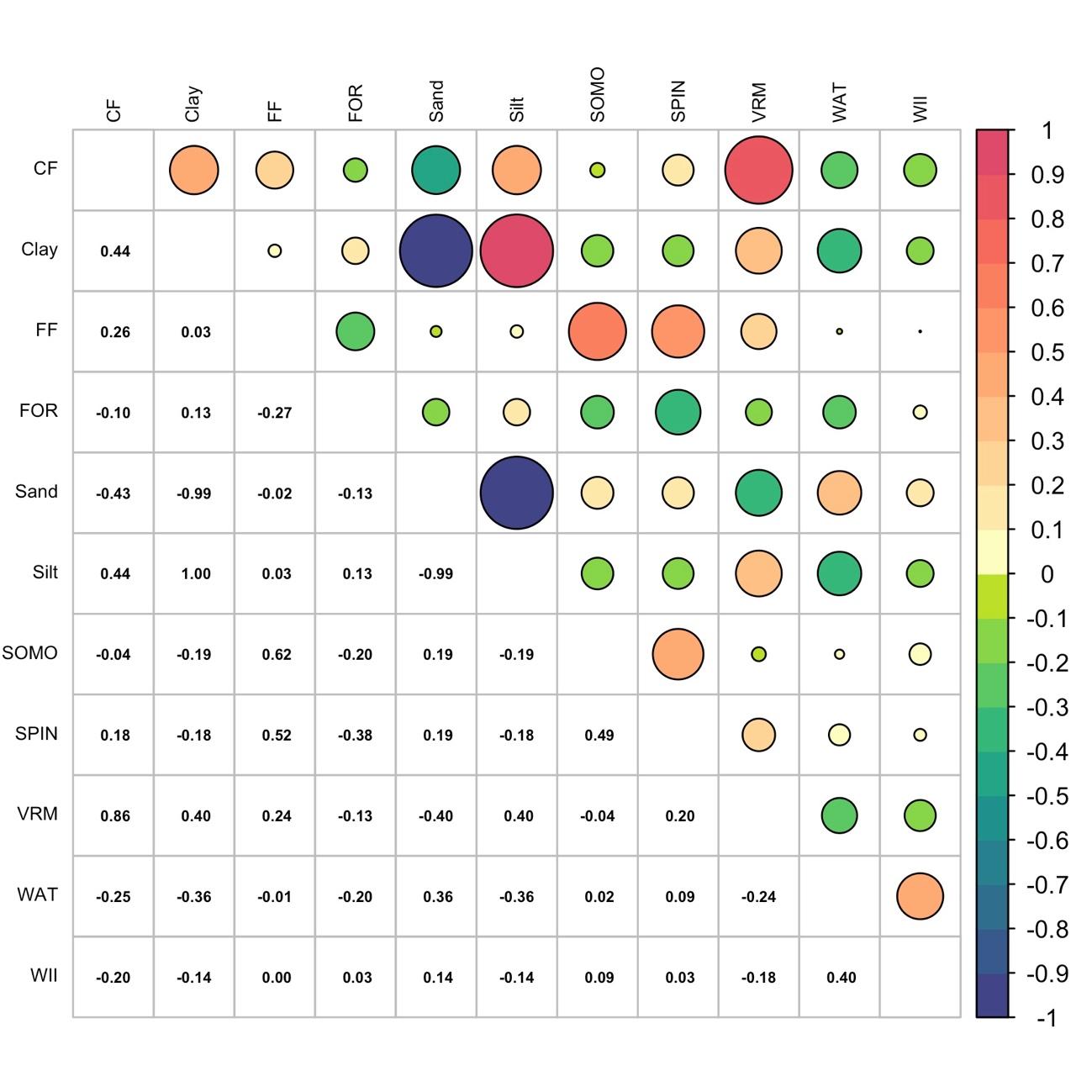


**Appendix S4. Population genetic structure.**

We visualised temporal patterns in our SNP data sets, using a Principal Coordinate Analysis (PCoA). We chose to explore SNP data sets only, given the overall lack of clustering in microsatellite data sets. We compared PCoA results, sample locations and collection years (Figure S4.1) and concluded that samples did not show any temporal clustering. One exception is the PCoA for *Pseudomys hermannsburgensis*, where one sample collected on Enderby Island in 1988 clusters separately to the rest of the data. Given the lack of temporal clustering in the rest of the data set (Figure S4.2), we took this genetic structure to represent genetic differentiation between island and mainland individuals. Additionally, we found no evidence for hierarchical genetic structuring for this species once the island individual was removed (Figure S4.2).


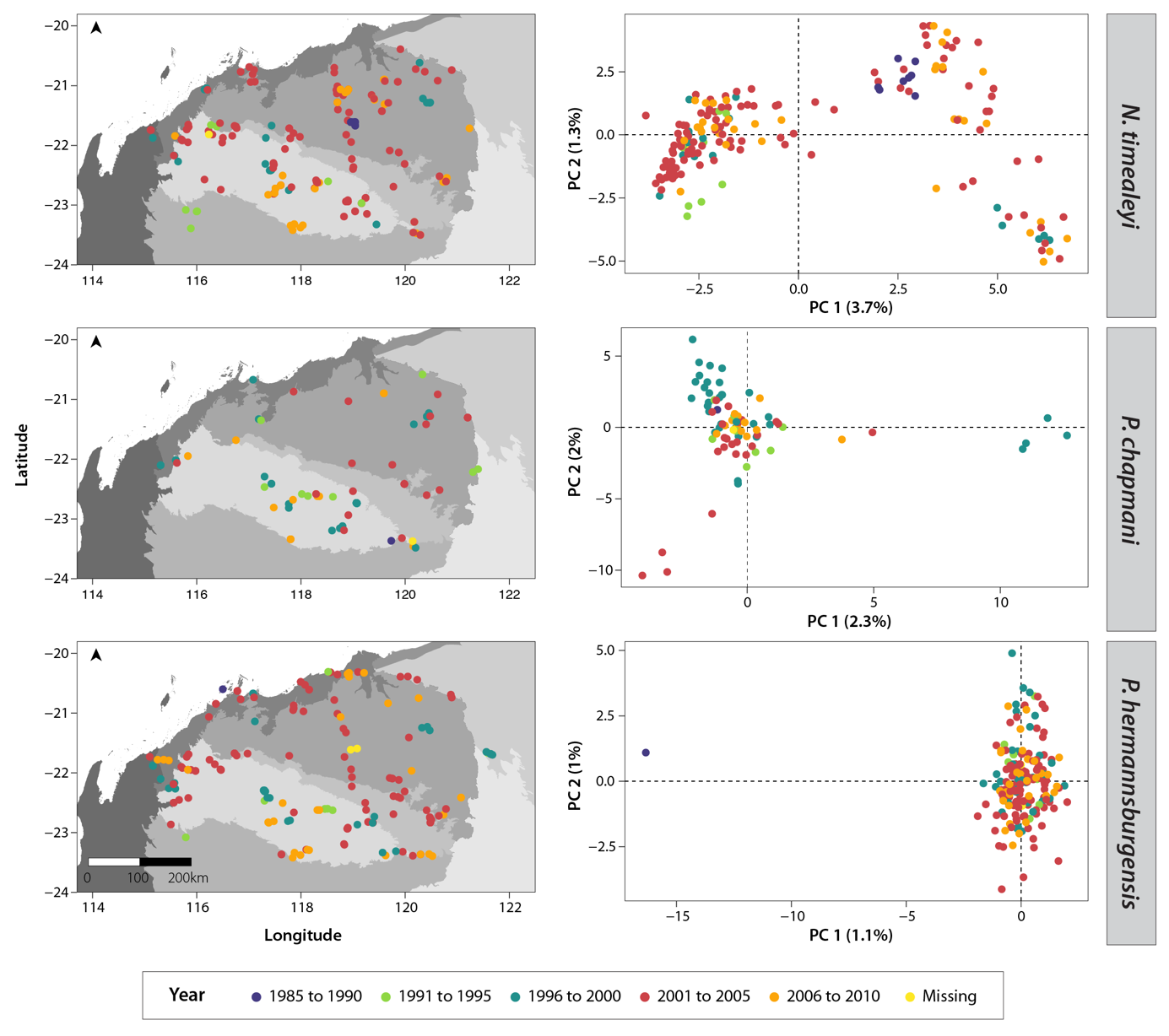


***Figure S4.1*** *Maps of the Pilbara and PCoA results (using the first two axes) for SNP data sets across the three study species, where grey colours delineate IBRA subregions and sample locations are coloured by collection year (grouped into five-year time slices).*


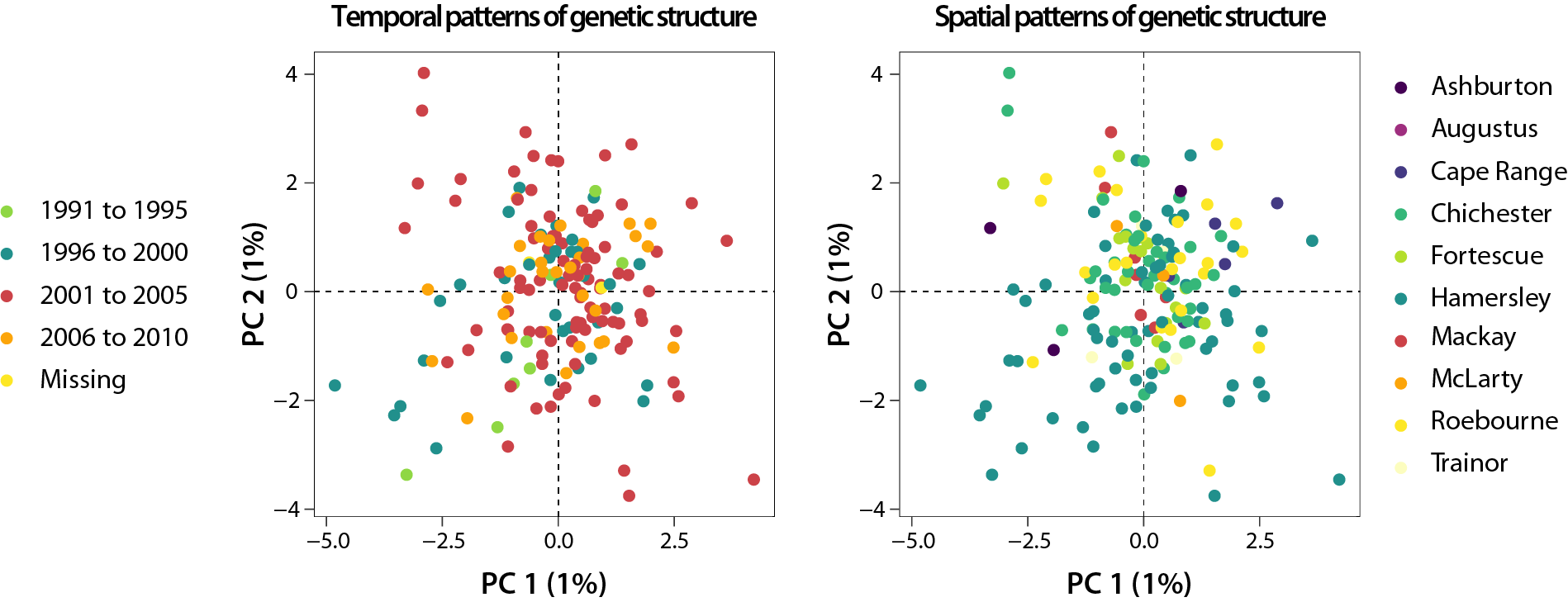


***Figure S4.2*** *PCoA results (using the first two axes) for Pseudomys hermannsburgensis SNP data, with the Enderby Island individual removed. Colours delineate collection year (grouped into five-year time slices, left) and IBRA subregions (right).*

**Appendix S5. *ResistanceGA* model selection.**

Full model selection results for single- and multi-surface optimised layers, for both marker types and all species, where k: number of parameters, AIC: Akaike Information Criterion, AICc: Akaike Information Criterion corrected for small sample size, R^2^m: marginal R^2^, R^2^c: conditional R^2^, LL: log-likelihood, ΔAICc: AICc ranking in relation to best performing model, AICc weight: Akaike weight indicating the relative likelihood of each model.

***N. timealeyi* SNP model selection**

| **Surface** | **k** | **AIC** | **AICc** | **R^2^m** | **R^2^c** | **LL** | **ΔAICc** | **AICc weight** |
| --- | --- | --- | --- | --- | --- | --- | --- | --- |
| SOMO29 * FOR * VRM | 10 | 15633.765 | 15635.646 | 0.634 | 0.933 | -7806.883 | 0 | 0.663 |
| SOMO29 * SPIN * VRM | 10 | 15635.118 | 15636.998 | 0.633 | 0.931 | -7807.559 | 1.352 | 0.337 |
| SOMO29 * WAT * FOR * VRM | 13 | 15704.683 | 15707.876 | 0.627 | 0.925 | -7839.341 | 72.23 | 0 |
| SOMO29 * WAT * SPIN * VRM | 13 | 15716.087 | 15719.28 | 0.634 | 0.935 | -7845.043 | 83.634 | 0 |
| SOMO29 * FOR * SPIN * VRM | 13 | 15766.908 | 15770.101 | 0.62 | 0.915 | -7870.454 | 134.455 | 0 |
| SOMO29 * DEM * VRM | 10 | 15830.301 | 15832.181 | 0.627 | 0.927 | -7905.15 | 196.535 | 0 |
| SOMO29 * Clay * FOR * VRM | 13 | 15829.918 | 15833.111 | 0.62 | 0.917 | -7901.959 | 197.465 | 0 |
| SOMO29 * VRM | 7 | 15833.786 | 15834.719 | 0.626 | 0.926 | -7909.893 | 199.074 | 0 |
| SOMO29 * DEM * FOR * VRM | 13 | 15835.903 | 15839.096 | 0.626 | 0.926 | -7904.951 | 203.45 | 0 |
| Clay * FOR * VRM | 10 | 15854.319 | 15856.199 | 0.635 | 0.941 | -7917.159 | 220.553 | 0 |
| SOMO29 * Clay * FOR | 10 | 15856.805 | 15858.686 | 0.626 | 0.933 | -7918.403 | 223.04 | 0 |
| SOMO29 * Clay * FOR * SPIN | 13 | 15873.547 | 15876.74 | 0.622 | 0.928 | -7923.773 | 241.094 | 0 |
| DEM * FOR * VRM | 10 | 15907.854 | 15909.735 | 0.636 | 0.939 | -7943.927 | 274.089 | 0 |
| DEM * FOR * SPIN * VRM | 13 | 15908.276 | 15911.469 | 0.636 | 0.94 | -7941.138 | 275.823 | 0 |
| SOMO29 * Clay * WAT * FOR | 13 | 15917.986 | 15921.179 | 0.602 | 0.9 | -7945.993 | 285.533 | 0 |
| Clay * VRM | 7 | 15935.455 | 15936.388 | 0.635 | 0.941 | -7960.728 | 300.743 | 0 |
| Clay * WAT * FOR * VRM | 13 | 15943.04 | 15946.233 | 0.634 | 0.938 | -7958.52 | 310.587 | 0 |
| DEM * VRM | 7 | 15948.139 | 15949.072 | 0.636 | 0.941 | -7967.069 | 313.426 | 0 |
| Clay * DEM * VRM | 10 | 15948.737 | 15950.617 | 0.636 | 0.941 | -7964.369 | 314.972 | 0 |
| SOMO29 * FOR | 7 | 15951.01 | 15951.944 | 0.614 | 0.918 | -7968.505 | 316.298 | 0 |
| FOR * VRM | 7 | 15955.772 | 15956.705 | 0.634 | 0.939 | -7970.886 | 321.059 | 0 |
| Clay * SPIN * VRM | 10 | 15955.006 | 15956.886 | 0.635 | 0.94 | -7967.503 | 321.24 | 0 |
| WAT * FOR * VRM | 10 | 15957.609 | 15959.489 | 0.636 | 0.941 | -7968.804 | 323.843 | 0 |
| Clay * FOR * SPIN * VRM | 13 | 15961.068 | 15964.261 | 0.632 | 0.936 | -7967.534 | 328.615 | 0 |
| SOMO29 * DEM * FOR | 10 | 15964.885 | 15966.766 | 0.612 | 0.916 | -7972.443 | 331.12 | 0 |
| SOMO29 * FOR * SPIN | 10 | 15965.022 | 15966.903 | 0.613 | 0.917 | -7972.511 | 331.257 | 0 |
| DEM * WAT * VRM | 10 | 15965.414 | 15967.295 | 0.634 | 0.937 | -7972.707 | 331.649 | 0 |
| SOMO29 * WAT * FOR | 10 | 15965.486 | 15967.366 | 0.613 | 0.917 | -7972.743 | 331.72 | 0 |
| WAT * SPIN * VRM | 10 | 15966.217 | 15968.097 | 0.636 | 0.941 | -7973.108 | 332.451 | 0 |
| DEM * WAT * FOR * VRM | 13 | 15966.335 | 15969.528 | 0.634 | 0.938 | -7970.168 | 333.882 | 0 |
| VRM | 4 | 15970.384 | 15970.71 | 0.631 | 0.934 | -7981.192 | 335.064 | 0 |
| SPIN * VRM | 7 | 15974.915 | 15975.848 | 0.626 | 0.926 | -7980.458 | 340.203 | 0 |
| Clay * WAT * VRM | 10 | 15975.05 | 15976.93 | 0.63 | 0.933 | -7977.525 | 341.285 | 0 |
| SOMO29 * DEM * FOR * SPIN | 13 | 15977.547 | 15980.74 | 0.611 | 0.914 | -7975.774 | 345.094 | 0 |
| FOR * SPIN * VRM | 10 | 15982.818 | 15984.699 | 0.635 | 0.941 | -7981.409 | 349.053 | 0 |
| SOMO29 * DEM * WAT * FOR | 13 | 15986.997 | 15990.19 | 0.612 | 0.914 | -7980.498 | 354.544 | 0 |
| Clay * DEM * WAT * VRM | 13 | 15990.001 | 15993.194 | 0.632 | 0.936 | -7982 | 357.548 | 0 |
| WAT * FOR * SPIN * VRM | 13 | 15990.324 | 15993.517 | 0.631 | 0.934 | -7982.162 | 357.871 | 0 |
| WAT * VRM | 7 | 15995.75 | 15996.683 | 0.628 | 0.93 | -7990.875 | 361.037 | 0 |
| DEM * SPIN * VRM | 10 | 16000.816 | 16002.696 | 0.63 | 0.933 | -7990.408 | 367.05 | 0 |
| SOMO29 * DEM * WAT * VRM | 13 | 16020.853 | 16024.046 | 0.593 | 0.881 | -7997.426 | 388.4 | 0 |
| Clay * DEM * SPIN * VRM | 13 | 16051.557 | 16054.75 | 0.619 | 0.918 | -8012.779 | 419.104 | 0 |
| SOMO29 * Clay * SPIN * VRM | 13 | 16051.739 | 16054.932 | 0.613 | 0.91 | -8012.87 | 419.286 | 0 |
| DEM * WAT * SPIN * VRM | 13 | 16052.599 | 16055.792 | 0.621 | 0.92 | -8013.3 | 420.146 | 0 |
| SOMO29 * Clay * VRM | 10 | 16062.292 | 16064.172 | 0.585 | 0.872 | -8021.146 | 428.526 | 0 |
| SOMO29 * DEM * SPIN * VRM | 13 | 16071.59 | 16074.783 | 0.58 | 0.863 | -8022.795 | 439.137 | 0 |
| SOMO29 * Clay | 7 | 16078.12 | 16079.054 | 0.592 | 0.889 | -8032.06 | 443.408 | 0 |
| SOMO29 * Clay * WAT | 10 | 16077.368 | 16079.248 | 0.597 | 0.896 | -8028.684 | 443.602 | 0 |
| SOMO29 * Clay * DEM | 10 | 16078.79 | 16080.67 | 0.602 | 0.902 | -8029.395 | 445.025 | 0 |
| SOMO29 * Clay * SPIN | 10 | 16079.503 | 16081.384 | 0.599 | 0.897 | -8029.752 | 445.738 | 0 |
| SOMO29 * Clay * DEM * VRM | 13 | 16088.067 | 16091.26 | 0.617 | 0.914 | -8031.033 | 455.614 | 0 |
| SOMO29 * Clay * WAT * VRM | 13 | 16107.03 | 16110.223 | 0.589 | 0.886 | -8040.515 | 474.577 | 0 |
| SOMO29 * Clay * DEM * SPIN | 13 | 16129.372 | 16132.565 | 0.59 | 0.887 | -8051.686 | 496.919 | 0 |
| SOMO29 * Clay * DEM * FOR | 13 | 16134.541 | 16137.734 | 0.582 | 0.876 | -8054.27 | 502.088 | 0 |
| SOMO29 * WAT * VRM | 10 | 16154.722 | 16156.602 | 0.57 | 0.851 | -8067.361 | 520.956 | 0 |
| SOMO29 * Clay * DEM * WAT | 13 | 16169.277 | 16172.47 | 0.585 | 0.881 | -8071.639 | 536.824 | 0 |
| Clay * DEM * FOR * SPIN | 13 | 16195.022 | 16198.215 | 0.649 | 0.952 | -8084.511 | 562.569 | 0 |
| SOMO29 | 4 | 16198.618 | 16198.943 | 0.549 | 0.838 | -8095.309 | 563.297 | 0 |
| SOMO29 * WAT * SPIN | 10 | 16201.053 | 16202.933 | 0.57 | 0.865 | -8090.527 | 567.288 | 0 |
| SOMO29 * WAT | 7 | 16206.535 | 16207.469 | 0.548 | 0.838 | -8096.268 | 571.823 | 0 |
| SOMO29 * SPIN | 7 | 16206.547 | 16207.48 | 0.548 | 0.838 | -8096.273 | 571.834 | 0 |
| SOMO29 * DEM | 7 | 16206.553 | 16207.486 | 0.548 | 0.838 | -8096.276 | 571.84 | 0 |
| SOMO29 * DEM * WAT | 10 | 16214.556 | 16216.436 | 0.548 | 0.837 | -8097.278 | 580.79 | 0 |
| SOMO29 * DEM * SPIN | 10 | 16214.595 | 16216.475 | 0.548 | 0.837 | -8097.297 | 580.829 | 0 |
| Clay * WAT * SPIN * VRM | 13 | 16214.201 | 16217.394 | 0.599 | 0.893 | -8094.1 | 581.748 | 0 |
| SOMO29 * DEM * WAT * SPIN | 13 | 16219.08 | 16222.273 | 0.571 | 0.863 | -8096.54 | 586.627 | 0 |
| SOMO29 * Clay * WAT * SPIN | 13 | 16287.645 | 16290.838 | 0.521 | 0.805 | -8130.822 | 655.192 | 0 |
| Clay * DEM * FOR | 10 | 16298.676 | 16300.557 | 0.636 | 0.955 | -8139.338 | 664.911 | 0 |
| Clay * DEM * FOR * VRM | 13 | 16326.348 | 16329.541 | 0.587 | 0.878 | -8150.174 | 693.895 | 0 |
| Clay * FOR | 7 | 16346.934 | 16347.867 | 0.638 | 0.959 | -8166.467 | 712.221 | 0 |
| SOMO29 * WAT * FOR * SPIN | 13 | 16344.745 | 16347.938 | 0.521 | 0.796 | -8159.373 | 712.292 | 0 |
| Clay * FOR * SPIN | 10 | 16353.018 | 16354.898 | 0.638 | 0.958 | -8166.509 | 719.252 | 0 |
| Clay * DEM * WAT * FOR | 13 | 16361.2 | 16364.393 | 0.627 | 0.942 | -8167.6 | 728.747 | 0 |
| Clay * WAT * FOR | 10 | 16408.053 | 16409.934 | 0.624 | 0.942 | -8194.027 | 774.288 | 0 |
| Clay * DEM * WAT * SPIN | 13 | 16441.611 | 16444.804 | 0.613 | 0.914 | -8207.805 | 809.158 | 0 |
| Clay * DEM * SPIN | 10 | 16444.078 | 16445.958 | 0.63 | 0.936 | -8212.039 | 810.313 | 0 |
| Clay * WAT * FOR * SPIN | 13 | 16512.836 | 16516.029 | 0.594 | 0.903 | -8243.418 | 880.383 | 0 |
| Clay * SPIN | 7 | 16529.857 | 16530.79 | 0.626 | 0.939 | -8257.928 | 895.144 | 0 |
| Clay | 4 | 16538.271 | 16538.596 | 0.613 | 0.927 | -8265.135 | 902.95 | 0 |
| Clay * WAT | 7 | 16540.178 | 16541.112 | 0.613 | 0.929 | -8263.089 | 905.466 | 0 |
| Clay * DEM | 7 | 16544.036 | 16544.969 | 0.614 | 0.929 | -8265.018 | 909.323 | 0 |
| Clay * DEM * WAT | 10 | 16545.844 | 16547.724 | 0.614 | 0.93 | -8262.922 | 912.078 | 0 |
| Clay * WAT * SPIN | 10 | 16548.119 | 16549.999 | 0.614 | 0.931 | -8264.059 | 914.353 | 0 |
| DEM * FOR * SPIN | 10 | 16622.234 | 16624.114 | 0.628 | 0.916 | -8301.117 | 988.468 | 0 |
| WAT * FOR * SPIN | 10 | 16642.016 | 16643.897 | 0.626 | 0.916 | -8311.008 | 1008.251 | 0 |
| FOR * SPIN | 7 | 16656.02 | 16656.953 | 0.638 | 0.936 | -8321.01 | 1021.307 | 0 |
| DEM * WAT * FOR * SPIN | 13 | 16661.554 | 16664.747 | 0.582 | 0.856 | -8317.777 | 1029.101 | 0 |
| DEM * WAT * SPIN | 10 | 16832.98 | 16834.86 | 0.56 | 0.833 | -8406.49 | 1199.215 | 0 |
| DEM * FOR | 7 | 16851.367 | 16852.3 | 0.622 | 0.936 | -8418.683 | 1216.654 | 0 |
| DEM * WAT * FOR | 10 | 16862.277 | 16864.157 | 0.624 | 0.939 | -8421.138 | 1228.511 | 0 |
| WAT * SPIN | 7 | 16868.067 | 16869.001 | 0.585 | 0.87 | -8427.034 | 1233.355 | 0 |
| DEM * SPIN | 7 | 16915.617 | 16916.55 | 0.64 | 0.95 | -8450.808 | 1280.904 | 0 |
| WAT * FOR | 7 | 16975.505 | 16976.438 | 0.604 | 0.918 | -8480.752 | 1340.792 | 0 |
| FOR | 4 | 16990.568 | 16990.893 | 0.6 | 0.914 | -8491.284 | 1355.248 | 0 |
| DEM * WAT | 7 | 17068.178 | 17069.111 | 0.489 | 0.775 | -8527.089 | 1433.465 | 0 |
| SPIN | 4 | 17122.811 | 17123.137 | 0.426 | 0.701 | -8557.406 | 1487.491 | 0 |
| DEM | 4 | 17156.252 | 17156.577 | 0.471 | 0.738 | -8574.126 | 1520.931 | 0 |
| WAT | 4 | 17170.965 | 17171.291 | 0.432 | 0.715 | -8581.483 | 1535.645 | 0 |
| IBD | 2 | 17205.241 | 17205.337 | 0.424 | 0.695 | -8600.621 | 1569.692 | 0 |
| Null | 1 | 22452.38 | 22452.412 | 0 | 0.276 | -11225.19 | 6816.766 | 0 |

***N. timealeyi* microsatellite model selection**

| **Surface** | **k** | **AIC** | **AICc** | **R^2^m** | **R^2^c** | **LL** | **ΔAICc** | **AICc weight** |
| --- | --- | --- | --- | --- | --- | --- | --- | --- |
| CF | 4 | 6038.217 | 6038.543 | 0.108 | 0.391 | -3015.109 | 0 | 0.965 |
| CF * SOMO29 | 7 | 6044.241 | 6045.174 | 0.109 | 0.392 | -3015.12 | 6.631 | 0.035 |
| SOMO29 | 4 | 6058.482 | 6058.808 | 0.028 | 0.34 | -3025.241 | 20.265 | 0 |
| IBD | 2 | 6065.601 | 6065.697 | 0.024 | 0.328 | -3030.801 | 27.155 | 0 |
| Null | 1 | 6251.634 | 6251.666 | 0 | 0.317 | -3124.817 | 213.124 | 0 |

***P. chapmani* SNP model selection**

| **Surface** | **k** | **AIC** | **AICc** | **R^2^m** | **R^2^c** | **LL** | **ΔAICc** | **AICc weight** |
| --- | --- | --- | --- | --- | --- | --- | --- | --- |
| SOMO29 * WII | 7 | 1871.037 | 1873.642 | 0.612 | 0.787 | -928.519 | 0 | 0.742 |
| SOMO29 * SPIN | 7 | 1873.208 | 1875.813 | 0.347 | 0.751 | -929.604 | 2.171 | 0.251 |
| SOMO29 * WAT * SPIN | 10 | 1879.216 | 1884.716 | 0.371 | 0.762 | -929.608 | 11.074 | 0.003 |
| SOMO29 * SPIN * WII | 10 | 1879.432 | 1884.932 | 0.371 | 0.763 | -929.716 | 11.29 | 0.003 |
| SOMO29 * SPIN * VRM | 10 | 1879.842 | 1885.342 | 0.391 | 0.772 | -929.921 | 11.7 | 0.002 |
| DEM * VRM * WII | 10 | 1885.972 | 1891.472 | 0.551 | 0.763 | -932.986 | 17.829 | 0 |
| VRM * WII | 7 | 1892.383 | 1894.988 | 0.555 | 0.756 | -939.192 | 21.346 | 0 |
| SOMO29 * DEM * SPIN * VRM | 13 | 1887.199 | 1897.037 | 0.386 | 0.77 | -930.6 | 23.395 | 0 |
| SOMO29 * DEM | 7 | 1896.481 | 1899.086 | 0.394 | 0.803 | -941.24 | 25.443 | 0 |
| SOMO29 * WAT * SPIN * VRM | 13 | 1889.337 | 1899.175 | 0.341 | 0.748 | -931.669 | 25.533 | 0 |
| SOMO29 * WAT * SPIN * WII | 13 | 1889.368 | 1899.206 | 0.312 | 0.733 | -931.684 | 25.564 | 0 |
| DEM | 4 | 1899.318 | 1900.187 | 0.394 | 0.812 | -945.659 | 26.545 | 0 |
| WAT * WII | 7 | 1898.106 | 1900.71 | 0.451 | 0.709 | -942.053 | 27.068 | 0 |
| SOMO29 * VRM | 7 | 1898.568 | 1901.172 | 0.272 | 0.704 | -942.284 | 27.53 | 0 |
| SOMO29 | 4 | 1902.226 | 1903.095 | 0.356 | 0.784 | -947.113 | 29.453 | 0 |
| SPIN * WII | 7 | 1903.488 | 1906.092 | 0.444 | 0.7 | -944.744 | 32.45 | 0 |
| DEM * VRM | 7 | 1905.338 | 1907.943 | 0.392 | 0.81 | -945.669 | 34.301 | 0 |
| DEM * WII | 7 | 1905.338 | 1907.943 | 0.392 | 0.81 | -945.669 | 34.301 | 0 |
| DEM * SPIN | 7 | 1905.338 | 1907.943 | 0.392 | 0.81 | -945.669 | 34.301 | 0 |
| DEM * WAT | 7 | 1905.338 | 1907.943 | 0.391 | 0.81 | -945.669 | 34.301 | 0 |
| SOMO29 * DEM * WII | 10 | 1902.624 | 1908.124 | 0.392 | 0.802 | -941.312 | 34.482 | 0 |
| SOMO29 * DEM * VRM | 10 | 1902.654 | 1908.154 | 0.392 | 0.802 | -941.327 | 34.512 | 0 |
| VRM | 4 | 1908.602 | 1909.471 | 0.223 | 0.685 | -950.301 | 35.829 | 0 |
| SOMO29 * WAT | 7 | 1907.379 | 1909.984 | 0.345 | 0.778 | -946.69 | 36.342 | 0 |
| SOMO29 * DEM * WAT | 10 | 1905.447 | 1910.947 | 0.309 | 0.751 | -942.723 | 37.305 | 0 |
| SOMO29 * DEM * SPIN * WII | 13 | 1902.941 | 1912.779 | 0.197 | 0.685 | -938.47 | 39.136 | 0 |
| SOMO29 * WAT * WII | 10 | 1908.188 | 1913.688 | 0.358 | 0.763 | -944.094 | 40.046 | 0 |
| SOMO29 * SPIN * VRM * WII | 13 | 1904.588 | 1914.426 | 0.186 | 0.677 | -939.294 | 40.783 | 0 |
| SOMO29 * DEM * WAT * SPIN | 13 | 1905.31 | 1915.148 | 0.214 | 0.694 | -939.655 | 41.506 | 0 |
| WAT * SPIN * WII | 10 | 1910.12 | 1915.62 | 0.463 | 0.702 | -945.06 | 41.978 | 0 |
| WII | 4 | 1914.946 | 1915.815 | 0.225 | 0.64 | -953.473 | 42.173 | 0 |
| SPIN * VRM | 7 | 1913.211 | 1915.816 | 0.285 | 0.703 | -949.606 | 42.174 | 0 |
| DEM * SPIN * WII | 10 | 1910.603 | 1916.103 | 0.371 | 0.792 | -945.302 | 42.461 | 0 |
| SOMO29 * VRM * WII | 10 | 1910.878 | 1916.378 | 0.282 | 0.724 | -945.439 | 42.736 | 0 |
| SPIN * VRM * WII | 10 | 1911.337 | 1916.837 | 0.339 | 0.649 | -945.669 | 43.195 | 0 |
| SOMO29 * DEM * SPIN | 10 | 1911.363 | 1916.863 | 0.391 | 0.809 | -945.681 | 43.22 | 0 |
| DEM * WAT * SPIN | 10 | 1911.369 | 1916.869 | 0.392 | 0.81 | -945.684 | 43.227 | 0 |
| DEM * WAT * VRM | 10 | 1911.369 | 1916.869 | 0.392 | 0.81 | -945.684 | 43.227 | 0 |
| DEM * WAT * WII | 10 | 1911.369 | 1916.869 | 0.392 | 0.81 | -945.685 | 43.227 | 0 |
| WAT | 4 | 1916.041 | 1916.91 | 0.194 | 0.709 | -954.02 | 43.268 | 0 |
| WAT * VRM | 7 | 1914.515 | 1917.12 | 0.241 | 0.692 | -950.258 | 43.478 | 0 |
| DEM * SPIN * VRM | 10 | 1911.738 | 1917.238 | 0.363 | 0.789 | -945.869 | 43.596 | 0 |
| SOMO29 * WAT * VRM | 10 | 1912.593 | 1918.093 | 0.35 | 0.78 | -946.297 | 44.451 | 0 |
| SOMO29 * DEM * WAT * WII | 13 | 1908.766 | 1918.604 | 0.396 | 0.804 | -941.383 | 44.962 | 0 |
| WAT * SPIN | 7 | 1916.992 | 1919.597 | 0.201 | 0.694 | -951.496 | 45.954 | 0 |
| SOMO29 * DEM * WAT * VRM | 13 | 1909.935 | 1919.773 | 0.34 | 0.769 | -941.967 | 46.13 | 0 |
| SPIN | 4 | 1921.638 | 1922.508 | 0.105 | 0.653 | -956.819 | 48.866 | 0 |
| IBD | 2 | 1923.269 | 1923.519 | 0.066 | 0.632 | -959.634 | 49.877 | 0 |
| SOMO29 * DEM * VRM * WII | 13 | 1914.226 | 1924.064 | 0.27 | 0.727 | -944.113 | 50.422 | 0 |
| DEM * SPIN * VRM * WII | 13 | 1917.399 | 1927.237 | 0.391 | 0.81 | -945.7 | 53.595 | 0 |
| DEM * WAT * VRM * WII | 13 | 1917.42 | 1927.258 | 0.397 | 0.813 | -945.71 | 53.616 | 0 |
| SOMO29 * WAT * VRM * WII | 13 | 1917.671 | 1927.509 | 0.311 | 0.752 | -945.835 | 53.867 | 0 |
| DEM * WAT * SPIN * VRM | 13 | 1918.464 | 1928.302 | 0.375 | 0.8 | -946.232 | 54.66 | 0 |
| DEM * WAT * SPIN * WII | 13 | 1918.966 | 1928.804 | 0.331 | 0.773 | -946.483 | 55.162 | 0 |
| WAT * SPIN * VRM | 10 | 1923.529 | 1929.029 | 0.178 | 0.686 | -951.764 | 55.386 | 0 |
| WAT * VRM * WII | 10 | 1928.181 | 1933.681 | 0.128 | 0.662 | -954.09 | 60.039 | 0 |
| WAT * SPIN * VRM * WII | 13 | 1934.177 | 1944.015 | 0.13 | 0.645 | -954.089 | 70.373 | 0 |
| Null | 1 | 2058.034 | 2058.116 | 0 | 0.604 | -1028.017 | 184.474 | 0 |

***P. chapmani* microsatellites model selection**

| **Surface** | **k** | **AIC** | **AICc** | **R^2^m** | **R^2^c** | **LL** | **ΔAICc** | **AICc weight** |
| --- | --- | --- | --- | --- | --- | --- | --- | --- |
| CF | 4 | 602.749 | 603.618 | 0.057 | 0.495 | -297.374 | 0 | 0.934 |
| IBD | 2 | 608.693 | 608.943 | 0.007 | 0.469 | -302.346 | 5.324 | 0.065 |
| Null | 1 | 617.985 | 618.066 | 0 | 0.466 | -307.992 | 14.448 | 0.001 |

***P. hermannsburgensis* SNP model selection**

| **Surface** | **k** | **AIC** | **AICc** | **R^2^m** | **R^2^c** | **LL** | **ΔAICc** | **AICc weight** |
| --- | --- | --- | --- | --- | --- | --- | --- | --- |
| VRM | 4 | 7082.838 | 7083.158 | 0.209 | 0.764 | -3537.419 | 0 | 0.785 |
| FF * VRM | 7 | 7088.851 | 7089.769 | 0.206 | 0.762 | -3537.426 | 6.611 | 0.029 |
| SOMO31 * VRM | 7 | 7088.863 | 7089.781 | 0.206 | 0.762 | -3537.431 | 6.623 | 0.029 |
| Silt * VRM | 7 | 7088.866 | 7089.784 | 0.206 | 0.762 | -3537.433 | 6.626 | 0.029 |
| VRM * WAT | 7 | 7088.866 | 7089.784 | 0.206 | 0.762 | -3537.433 | 6.626 | 0.029 |
| VRM * WII | 7 | 7088.866 | 7089.784 | 0.206 | 0.762 | -3537.433 | 6.626 | 0.029 |
| SPIN * VRM | 7 | 7088.866 | 7089.784 | 0.206 | 0.762 | -3537.433 | 6.626 | 0.029 |
| DEM * VRM | 7 | 7088.866 | 7089.784 | 0.206 | 0.763 | -3537.433 | 6.626 | 0.029 |
| FF * VRM * WII | 10 | 7094.881 | 7096.73 | 0.186 | 0.752 | -3537.441 | 13.572 | 0.001 |
| DEM * FF * VRM | 10 | 7095.075 | 7096.924 | 0.197 | 0.758 | -3537.537 | 13.766 | 0.001 |
| FF * Silt * VRM | 10 | 7095.075 | 7096.924 | 0.197 | 0.758 | -3537.538 | 13.766 | 0.001 |
| FF * SOMO31 * VRM | 10 | 7095.075 | 7096.924 | 0.197 | 0.758 | -3537.538 | 13.766 | 0.001 |
| FF * VRM * WAT | 10 | 7095.076 | 7096.924 | 0.197 | 0.758 | -3537.538 | 13.767 | 0.001 |
| Silt * SOMO31 * VRM | 10 | 7095.087 | 7096.936 | 0.197 | 0.758 | -3537.543 | 13.778 | 0.001 |
| DEM * SOMO31 * VRM | 10 | 7095.087 | 7096.936 | 0.197 | 0.758 | -3537.543 | 13.778 | 0.001 |
| SOMO31 * VRM * WII | 10 | 7095.087 | 7096.936 | 0.197 | 0.758 | -3537.544 | 13.778 | 0.001 |
| SPIN * VRM * WII | 10 | 7095.089 | 7096.938 | 0.197 | 0.758 | -3537.545 | 13.78 | 0.001 |
| Silt * SPIN * VRM | 10 | 7095.089 | 7096.938 | 0.197 | 0.758 | -3537.545 | 13.78 | 0.001 |
| DEM * VRM * WAT | 10 | 7095.089 | 7096.938 | 0.197 | 0.758 | -3537.545 | 13.78 | 0.001 |
| Silt * VRM * WII | 10 | 7095.09 | 7096.938 | 0.197 | 0.758 | -3537.545 | 13.78 | 0.001 |
| DEM * VRM * WII | 10 | 7095.09 | 7096.938 | 0.197 | 0.758 | -3537.545 | 13.78 | 0.001 |
| SPIN * VRM * WAT | 10 | 7095.09 | 7096.939 | 0.197 | 0.758 | -3537.545 | 13.781 | 0.001 |
| DEM * SPIN * VRM | 10 | 7095.09 | 7096.939 | 0.197 | 0.758 | -3537.545 | 13.781 | 0.001 |
| SOMO31 * SPIN * VRM | 10 | 7095.26 | 7097.109 | 0.192 | 0.755 | -3537.63 | 13.951 | 0.001 |
| DEM * Silt * VRM | 10 | 7096.154 | 7098.003 | 0.185 | 0.752 | -3538.077 | 14.845 | 0 |
| VRM * WAT * WII | 10 | 7097.93 | 7099.779 | 0.162 | 0.739 | -3538.965 | 16.621 | 0 |
| SOMO31 * VRM * WAT | 10 | 7098.192 | 7100.041 | 0.16 | 0.738 | -3539.096 | 16.883 | 0 |
| FF * SPIN * VRM | 10 | 7098.24 | 7100.088 | 0.161 | 0.739 | -3539.12 | 16.93 | 0 |
| Silt * VRM * WAT | 10 | 7099.24 | 7101.089 | 0.158 | 0.738 | -3539.62 | 17.931 | 0 |
| FF * SOMO31 * SPIN * VRM | 13 | 7101.477 | 7104.615 | 0.188 | 0.753 | -3537.739 | 21.457 | 0 |
| FF * Silt * VRM * WAT | 13 | 7101.477 | 7104.615 | 0.188 | 0.753 | -3537.739 | 21.457 | 0 |
| FF * Silt * VRM * WII | 13 | 7101.477 | 7104.615 | 0.188 | 0.753 | -3537.739 | 21.457 | 0 |
| FF * SOMO31 * VRM * WII | 13 | 7101.477 | 7104.615 | 0.188 | 0.753 | -3537.739 | 21.458 | 0 |
| FF * SPIN * VRM * WAT | 13 | 7101.478 | 7104.616 | 0.188 | 0.753 | -3537.739 | 21.458 | 0 |
| Silt * SOMO31 * VRM * WII | 13 | 7101.49 | 7104.628 | 0.188 | 0.753 | -3537.745 | 21.47 | 0 |
| Silt * SOMO31 * VRM * WAT | 13 | 7101.49 | 7104.628 | 0.188 | 0.753 | -3537.745 | 21.47 | 0 |
| SOMO31 * VRM * WAT * WII | 13 | 7101.49 | 7104.628 | 0.188 | 0.753 | -3537.745 | 21.47 | 0 |
| DEM * SPIN * VRM * WII | 13 | 7101.703 | 7104.841 | 0.187 | 0.753 | -3537.851 | 21.683 | 0 |
| Silt * VRM * WAT * WII | 13 | 7102.324 | 7105.462 | 0.18 | 0.749 | -3538.162 | 22.304 | 0 |
| DEM * FF * VRM * WII | 13 | 7102.357 | 7105.495 | 0.176 | 0.747 | -3538.179 | 22.337 | 0 |
| DEM * Silt * VRM * WII | 13 | 7102.391 | 7105.529 | 0.17 | 0.743 | -3538.196 | 22.371 | 0 |
| DEM * SOMO31 * VRM * WAT | 13 | 7102.498 | 7105.636 | 0.178 | 0.748 | -3538.249 | 22.478 | 0 |
| DEM * SOMO31 * VRM * WII | 13 | 7102.56 | 7105.698 | 0.173 | 0.746 | -3538.28 | 22.54 | 0 |
| DEM * Silt * VRM * WAT | 13 | 7102.619 | 7105.757 | 0.176 | 0.747 | -3538.31 | 22.599 | 0 |
| SPIN * VRM * WAT * WII | 13 | 7102.786 | 7105.924 | 0.172 | 0.745 | -3538.393 | 22.766 | 0 |
| DEM * FF * VRM * WAT | 13 | 7103.009 | 7106.146 | 0.175 | 0.747 | -3538.504 | 22.989 | 0 |
| SOMO31 * SPIN * VRM * WII | 13 | 7103.084 | 7106.222 | 0.166 | 0.742 | -3538.542 | 23.064 | 0 |
| Silt * SPIN * VRM * WII | 13 | 7103.51 | 7106.648 | 0.168 | 0.743 | -3538.755 | 23.49 | 0 |
| DEM * SPIN * VRM * WAT | 13 | 7103.925 | 7107.063 | 0.166 | 0.742 | -3538.962 | 23.905 | 0 |
| DEM * FF * SOMO31 * VRM | 13 | 7104.102 | 7107.24 | 0.168 | 0.743 | -3539.051 | 24.082 | 0 |
| FF * SPIN * VRM * WII | 13 | 7106.391 | 7109.529 | 0.145 | 0.731 | -3540.196 | 26.371 | 0 |
| FF * Silt * SOMO31 * VRM | 13 | 7107.139 | 7110.277 | 0.139 | 0.728 | -3540.569 | 27.119 | 0 |
| DEM * Silt * SOMO31 * VRM | 13 | 7107.993 | 7111.131 | 0.14 | 0.728 | -3540.996 | 27.973 | 0 |
| DEM * FF * SPIN * VRM | 13 | 7108.182 | 7111.32 | 0.137 | 0.727 | -3541.091 | 28.162 | 0 |
| FF * SOMO31 * VRM * WAT | 13 | 7110.234 | 7113.372 | 0.131 | 0.724 | -3542.117 | 30.214 | 0 |
| DEM * FF * Silt * VRM | 13 | 7115.491 | 7118.629 | 0.119 | 0.719 | -3544.745 | 35.471 | 0 |
| DEM * VRM * WAT * WII | 13 | 7117.018 | 7120.156 | 0.096 | 0.705 | -3545.509 | 36.998 | 0 |
| DEM * Silt * SPIN * VRM | 13 | 7118.091 | 7121.229 | 0.095 | 0.705 | -3546.045 | 38.071 | 0 |
| SOMO31 * SPIN * VRM * WAT | 13 | 7118.322 | 7121.459 | 0.09 | 0.701 | -3546.161 | 38.302 | 0 |
| FF * VRM * WAT * WII | 13 | 7119.848 | 7122.986 | 0.099 | 0.71 | -3546.924 | 39.828 | 0 |
| FF * Silt * SPIN * VRM | 13 | 7127.331 | 7130.469 | 0.07 | 0.692 | -3550.665 | 47.311 | 0 |
| Silt * SOMO31 * SPIN * VRM | 13 | 7130.086 | 7133.224 | 0.061 | 0.685 | -3552.043 | 50.066 | 0 |
| DEM * SOMO31 * SPIN * VRM | 13 | 7137.713 | 7140.851 | 0.055 | 0.684 | -3555.857 | 57.693 | 0 |
| Silt | 4 | 7142.287 | 7142.607 | 0.057 | 0.68 | -3567.143 | 59.449 | 0 |
| FF * Silt | 7 | 7146.819 | 7147.737 | 0.039 | 0.675 | -3566.41 | 64.579 | 0 |
| Silt * SPIN | 7 | 7148.562 | 7149.48 | 0.048 | 0.676 | -3567.281 | 66.322 | 0 |
| DEM * Silt | 7 | 7148.564 | 7149.482 | 0.048 | 0.676 | -3567.282 | 66.324 | 0 |
| Silt * SOMO31 | 7 | 7148.569 | 7149.487 | 0.048 | 0.676 | -3567.285 | 66.33 | 0 |
| Silt * WAT | 7 | 7148.571 | 7149.489 | 0.048 | 0.676 | -3567.285 | 66.331 | 0 |
| Silt * WII | 7 | 7148.591 | 7149.509 | 0.047 | 0.675 | -3567.295 | 66.351 | 0 |
| WAT | 4 | 7151.582 | 7151.902 | 0.037 | 0.658 | -3571.791 | 68.744 | 0 |
| SOMO31 | 4 | 7154.595 | 7154.915 | 0.045 | 0.669 | -3573.297 | 71.757 | 0 |
| Silt * SPIN * WII | 10 | 7153.14 | 7154.989 | 0.031 | 0.668 | -3566.57 | 71.831 | 0 |
| FF * Silt * WII | 10 | 7153.915 | 7155.763 | 0.035 | 0.673 | -3566.957 | 72.606 | 0 |
| FF * Silt * SOMO31 | 10 | 7154.029 | 7155.878 | 0.035 | 0.672 | -3567.015 | 72.72 | 0 |
| DEM * FF * Silt | 10 | 7154.052 | 7155.901 | 0.036 | 0.673 | -3567.026 | 72.743 | 0 |
| DEM * Silt * SOMO31 | 10 | 7155.078 | 7156.927 | 0.043 | 0.673 | -3567.539 | 73.769 | 0 |
| Silt * SOMO31 * WAT | 10 | 7155.08 | 7156.929 | 0.044 | 0.674 | -3567.54 | 73.771 | 0 |
| Silt * SOMO31 * WII | 10 | 7155.082 | 7156.931 | 0.044 | 0.674 | -3567.541 | 73.773 | 0 |
| DEM * Silt * WAT | 10 | 7155.343 | 7157.192 | 0.04 | 0.672 | -3567.672 | 74.034 | 0 |
| Silt * WAT * WII | 10 | 7155.394 | 7157.243 | 0.045 | 0.674 | -3567.697 | 74.085 | 0 |
| FF * Silt * WAT | 10 | 7155.649 | 7157.497 | 0.03 | 0.672 | -3567.824 | 74.339 | 0 |
| DEM * Silt * WII | 10 | 7155.66 | 7157.508 | 0.038 | 0.671 | -3567.83 | 74.351 | 0 |
| FF * WAT | 7 | 7157.312 | 7158.23 | 0.038 | 0.658 | -3571.656 | 75.072 | 0 |
| WAT * WII | 7 | 7157.375 | 7158.294 | 0.038 | 0.658 | -3571.688 | 75.136 | 0 |
| SPIN * WAT | 7 | 7158.127 | 7159.045 | 0.036 | 0.657 | -3572.063 | 75.887 | 0 |
| DEM * WAT | 7 | 7158.286 | 7159.204 | 0.035 | 0.657 | -3572.143 | 76.046 | 0 |
| Silt * SPIN * VRM * WAT | 13 | 7156.901 | 7160.039 | 0.025 | 0.67 | -3565.451 | 76.881 | 0 |
| DEM * Silt * SPIN | 10 | 7158.993 | 7160.842 | 0.026 | 0.665 | -3569.497 | 77.684 | 0 |
| FF * SOMO31 | 7 | 7160.425 | 7161.343 | 0.048 | 0.67 | -3573.213 | 78.185 | 0 |
| SOMO31 * SPIN | 7 | 7160.574 | 7161.492 | 0.048 | 0.671 | -3573.287 | 78.334 | 0 |
| SOMO31 * WAT | 7 | 7160.574 | 7161.492 | 0.048 | 0.671 | -3573.287 | 78.334 | 0 |
| SOMO31 * WII | 7 | 7160.574 | 7161.492 | 0.048 | 0.671 | -3573.287 | 78.334 | 0 |
| DEM * SOMO31 | 7 | 7160.574 | 7161.492 | 0.048 | 0.671 | -3573.287 | 78.334 | 0 |
| Silt * SPIN * WAT | 10 | 7159.981 | 7161.83 | 0.022 | 0.663 | -3569.99 | 78.672 | 0 |
| DEM * Silt * SPIN * WII | 13 | 7159.527 | 7162.665 | 0.03 | 0.668 | -3566.764 | 79.507 | 0 |
| Silt * SPIN * WAT * WII | 13 | 7159.837 | 7162.975 | 0.029 | 0.667 | -3566.918 | 79.817 | 0 |
| FF * Silt * SPIN | 10 | 7161.195 | 7163.044 | 0.021 | 0.664 | -3570.597 | 79.886 | 0 |
| FF * Silt * SPIN * WII | 13 | 7160.27 | 7163.408 | 0.032 | 0.668 | -3567.135 | 80.25 | 0 |
| DEM * FF * Silt * SPIN | 13 | 7160.366 | 7163.504 | 0.027 | 0.667 | -3567.183 | 80.346 | 0 |
| FF * Silt * WAT * WII | 13 | 7161.098 | 7164.236 | 0.034 | 0.671 | -3567.549 | 81.078 | 0 |
| DEM * FF * Silt * SOMO31 | 13 | 7161.483 | 7164.621 | 0.041 | 0.672 | -3567.741 | 81.463 | 0 |
| FF * Silt * SOMO31 * SPIN | 13 | 7161.489 | 7164.627 | 0.041 | 0.672 | -3567.745 | 81.469 | 0 |
| Silt * SOMO31 * WAT * WII | 13 | 7161.531 | 7164.669 | 0.041 | 0.672 | -3567.765 | 81.511 | 0 |
| FF * Silt * SOMO31 * WAT | 13 | 7161.729 | 7164.867 | 0.034 | 0.67 | -3567.864 | 81.709 | 0 |
| FF * Silt * SOMO31 * WII | 13 | 7162.103 | 7165.241 | 0.033 | 0.669 | -3568.052 | 82.084 | 0 |
| SPIN * WAT * WII | 10 | 7163.45 | 7165.299 | 0.038 | 0.658 | -3571.725 | 82.141 | 0 |
| DEM * WAT * WII | 10 | 7163.452 | 7165.301 | 0.038 | 0.658 | -3571.726 | 82.143 | 0 |
| FF * WAT * WII | 10 | 7164.014 | 7165.863 | 0.036 | 0.657 | -3572.007 | 82.705 | 0 |
| FF * SPIN * WAT | 10 | 7164.019 | 7165.868 | 0.036 | 0.657 | -3572.01 | 82.71 | 0 |
| DEM * SPIN * WAT | 10 | 7164.26 | 7166.109 | 0.035 | 0.657 | -3572.13 | 82.951 | 0 |
| FF * Silt * SPIN * WAT | 13 | 7163.981 | 7167.119 | 0.021 | 0.664 | -3568.991 | 83.961 | 0 |
| FF | 4 | 7167.009 | 7167.329 | 0.042 | 0.652 | -3579.504 | 84.171 | 0 |
| DEM * Silt * SPIN * WAT | 13 | 7164.242 | 7167.379 | 0.026 | 0.665 | -3569.121 | 84.222 | 0 |
| Silt * SOMO31 * SPIN | 10 | 7166.43 | 7168.279 | 0.055 | 0.675 | -3573.215 | 85.121 | 0 |
| FF * SOMO31 * WII | 10 | 7166.568 | 7168.417 | 0.048 | 0.671 | -3573.284 | 85.259 | 0 |
| FF * SOMO31 * SPIN | 10 | 7166.568 | 7168.417 | 0.048 | 0.671 | -3573.284 | 85.259 | 0 |
| DEM * SOMO31 * WII | 10 | 7166.599 | 7168.447 | 0.048 | 0.671 | -3573.299 | 85.29 | 0 |
| SOMO31 * SPIN * WII | 10 | 7166.599 | 7168.447 | 0.048 | 0.671 | -3573.299 | 85.29 | 0 |
| DEM * SOMO31 * SPIN | 10 | 7166.599 | 7168.448 | 0.048 | 0.671 | -3573.3 | 85.29 | 0 |
| DEM * Silt * WAT * WII | 13 | 7165.325 | 7168.463 | 0.024 | 0.664 | -3569.663 | 85.305 | 0 |
| SOMO31 * WAT * WII | 10 | 7166.703 | 7168.552 | 0.047 | 0.671 | -3573.352 | 85.394 | 0 |
| DEM * FF * Silt * WII | 13 | 7165.456 | 7168.594 | 0.023 | 0.664 | -3569.728 | 85.436 | 0 |
| SOMO31 * SPIN * WAT | 10 | 7166.766 | 7168.615 | 0.041 | 0.668 | -3573.383 | 85.457 | 0 |
| IBD | 2 | 7168.675 | 7168.77 | 0.011 | 0.656 | -3582.338 | 85.612 | 0 |
| FF * SOMO31 * WAT | 10 | 7167.191 | 7169.04 | 0.036 | 0.665 | -3573.596 | 85.882 | 0 |
| DEM * SOMO31 * WAT | 10 | 7167.244 | 7169.093 | 0.039 | 0.667 | -3573.622 | 85.935 | 0 |
| WII | 4 | 7169.493 | 7169.813 | 0.013 | 0.655 | -3580.746 | 86.655 | 0 |
| DEM * FF * Silt * WAT | 13 | 7167.051 | 7170.189 | 0.02 | 0.664 | -3570.526 | 87.031 | 0 |
| DEM * FF | 7 | 7169.888 | 7170.806 | 0.052 | 0.658 | -3577.944 | 87.648 | 0 |
| DEM * FF * SOMO31 | 10 | 7169.234 | 7171.083 | 0.025 | 0.66 | -3574.617 | 87.925 | 0 |
| SPIN | 4 | 7171.197 | 7171.517 | 0.013 | 0.661 | -3581.599 | 88.359 | 0 |
| FF * SPIN | 7 | 7170.737 | 7171.655 | 0.041 | 0.653 | -3578.368 | 88.497 | 0 |
| DEM * Silt * SOMO31 * WII | 13 | 7168.522 | 7171.66 | 0.018 | 0.662 | -3571.261 | 88.502 | 0 |
| DEM * FF * WAT | 10 | 7170.169 | 7172.018 | 0.036 | 0.651 | -3575.084 | 88.86 | 0 |
| DEM * FF * SPIN * WAT | 13 | 7169.795 | 7172.933 | 0.037 | 0.657 | -3571.898 | 89.776 | 0 |
| DEM | 4 | 7172.666 | 7172.986 | 0.011 | 0.656 | -3582.333 | 89.828 | 0 |
| DEM * SPIN * WAT * WII | 13 | 7169.855 | 7172.993 | 0.037 | 0.658 | -3571.927 | 89.835 | 0 |
| DEM * FF * WAT * WII | 13 | 7170.237 | 7173.375 | 0.036 | 0.657 | -3572.119 | 90.217 | 0 |
| FF * WII | 7 | 7173.011 | 7173.929 | 0.042 | 0.652 | -3579.506 | 90.771 | 0 |
| SPIN * WII | 7 | 7174.113 | 7175.031 | 0.012 | 0.661 | -3580.057 | 91.874 | 0 |
| FF * SOMO31 * SPIN * WII | 13 | 7172.593 | 7175.731 | 0.047 | 0.671 | -3573.296 | 92.573 | 0 |
| FF * SOMO31 * WAT * WII | 13 | 7172.623 | 7175.761 | 0.045 | 0.67 | -3573.311 | 92.603 | 0 |
| FF * SOMO31 * SPIN * WAT | 13 | 7172.642 | 7175.779 | 0.05 | 0.672 | -3573.321 | 92.622 | 0 |
| DEM * SOMO31 * SPIN * WII | 13 | 7172.689 | 7175.827 | 0.05 | 0.672 | -3573.345 | 92.669 | 0 |
| Silt * SOMO31 * SPIN * WII | 13 | 7172.721 | 7175.859 | 0.044 | 0.669 | -3573.361 | 92.702 | 0 |
| DEM * FF * SOMO31 * WII | 13 | 7172.722 | 7175.86 | 0.045 | 0.667 | -3573.361 | 92.703 | 0 |
| DEM * SPIN | 7 | 7174.983 | 7175.901 | 0.018 | 0.662 | -3580.491 | 92.743 | 0 |
| DEM * SOMO31 * SPIN * WAT | 13 | 7172.832 | 7175.97 | 0.046 | 0.67 | -3573.416 | 92.812 | 0 |
| Silt * SOMO31 * SPIN * WAT | 13 | 7172.839 | 7175.977 | 0.017 | 0.658 | -3573.42 | 92.82 | 0 |
| DEM * FF * SOMO31 * WAT | 13 | 7173.232 | 7176.37 | 0.053 | 0.674 | -3573.616 | 93.212 | 0 |
| DEM * FF * SOMO31 * SPIN | 13 | 7173.271 | 7176.409 | 0.044 | 0.67 | -3573.635 | 93.251 | 0 |
| DEM * WII | 7 | 7175.497 | 7176.415 | 0.013 | 0.655 | -3580.748 | 93.257 | 0 |
| DEM * SOMO31 * WAT * WII | 13 | 7173.376 | 7176.514 | 0.038 | 0.666 | -3573.688 | 93.356 | 0 |
| FF * SPIN * WAT * WII | 13 | 7173.648 | 7176.786 | 0.028 | 0.655 | -3573.824 | 93.628 | 0 |
| DEM * FF * WII | 10 | 7175.908 | 7177.756 | 0.053 | 0.656 | -3577.954 | 94.599 | 0 |
| SOMO31 * SPIN * WAT * WII | 13 | 7174.756 | 7177.894 | 0.045 | 0.67 | -3574.378 | 94.736 | 0 |
| DEM * FF * SPIN | 10 | 7176.05 | 7177.898 | 0.053 | 0.653 | -3578.025 | 94.74 | 0 |
| FF * SPIN * WII | 10 | 7177.433 | 7179.282 | 0.045 | 0.654 | -3578.717 | 96.124 | 0 |
| DEM * Silt * SOMO31 * SPIN | 13 | 7176.875 | 7180.013 | 0.022 | 0.66 | -3575.438 | 96.855 | 0 |
| DEM * SPIN * WII | 10 | 7179.328 | 7181.177 | 0.017 | 0.66 | -3579.664 | 98.019 | 0 |
| DEM * Silt * SOMO31 * WAT | 13 | 7182.415 | 7185.553 | 0.011 | 0.655 | -3578.208 | 102.395 | 0 |
| DEM * FF * SPIN * WII | 13 | 7182.906 | 7186.044 | 0.042 | 0.653 | -3578.453 | 102.886 | 0 |
| Null | 1 | 7330.114 | 7330.145 | 0 | 0.654 | -3664.057 | 246.987 | 0 |

***P. hermannsburgensis* microsatellite model selection**

| **Surface** | **k** | **AIC** | **AICc** | **R^2^m** | **R^2^c** | **LL** | **ΔAICc** | **AICc weight** |
| --- | --- | --- | --- | --- | --- | --- | --- | --- |
| Clay | 4 | 4698.537 | 4698.857 | 0.025 | 0.407 | -2345.268 | 0 | 0.798 |
| Clay * SOMO31 | 7 | 4702.521 | 4703.439 | 0.011 | 0.399 | -2344.261 | 4.582 | 0.081 |
| Clay * VRM | 7 | 4703.389 | 4704.307 | 0.013 | 0.403 | -2344.695 | 5.45 | 0.052 |
| SOMO31 | 4 | 4704.894 | 4705.214 | 0.013 | 0.404 | -2348.447 | 6.357 | 0.033 |
| VRM | 4 | 4705.581 | 4705.901 | 0.012 | 0.41 | -2348.791 | 7.045 | 0.024 |
| IBD | 2 | 4708.374 | 4708.468 | 0.002 | 0.396 | -2352.187 | 9.611 | 0.007 |
| SOMO31 * VRM | 7 | 4708.65 | 4709.568 | 0.015 | 0.409 | -2347.325 | 10.712 | 0.004 |
| Clay * SOMO31 * VRM | 10 | 4709.859 | 4711.708 | 0.014 | 0.408 | -2344.929 | 12.851 | 0.001 |
| Null | 1 | 4726.12 | 4726.151 | 0 | 0.395 | -2362.06 | 27.294 | 0 |

**Appendix S6. *ResistanceGA* MLPE model summaries.**

MLPE model summaries for the best-performing model across marker types and species.

| ***N. timealeyi:* Microsatellites** | **response** | | |
| --- | --- | --- | --- |
| *Predictors* | *Estimates* | *CI* | *p* |
| (Intercept) | 6.36 | 6.28 – 6.44 | **<0.001** |
| CF | 0.14 | 0.12 – 0.16 | **<0.001** |
| **Random Effects** | | | |
| σ^2^ | 0.12 | | |
| τ_00_ _pop1_ | 0.05 | | |
| ICC | 0.32 | | |
| N _pop1_ | 128 | | |
| Observations | 8128 | | |
| Marginal R^2^ / Conditional R^2^ | 0.108 / 0.391 | | |
| AICc | 6050.224 | | |
| ***N. timealeyi:* SNPs** | **response** | | |
| *Predictors* | *Estimates* | *CI* | *p* |
| (Intercept) | 48.95 | 48.51 – 49.40 | **<0.001** |
| FOR*SOMO29*VRM | 1.86 | 1.82 – 1.89 | **<0.001** |
| **Random Effects** | | | |
| σ^2^ | 0.36 | | |
| τ_00_ _pop1_ | 1.63 | | |
| ICC | 0.82 | | |
| N _pop1_ | 128 | | |
| Observations | 8128 | | |
| Marginal R^2^ / Conditional R^2^ | 0.634 / 0.933 | | |
| AICc | 15629.156 | | |

| ***P. chapmani:* Microsatellites** | **response** | | |
| --- | --- | --- | --- |
| *Predictors* | *Estimates* | *CI* | *p* |
| (Intercept) | 7.33 | 7.18 – 7.47 | **<0.001** |
| CF | 0.09 | 0.05 – 0.14 | **<0.001** |
| **Random Effects** | | | |
| σ^2^ | 0.08 | | |
| τ_00_ _pop1_ | 0.07 | | |
| ICC | 0.46 | | |
| N _pop1_ | 51 | | |
| Observations | 1275 | | |
| Marginal R^2^ / Conditional R^2^ | 0.057 / 0.495 | | |
| AICc | 612.075 | | |
| ***P. chapmani:* SNPs** | **response** | | |
| *Predictors* | *Estimates* | *CI* | *p* |
| (Intercept) | 55.71 | 55.48 – 55.94 | **<0.001** |
| SOMO29*WII | 0.79 | 0.69 – 0.89 | **<0.001** |
| **Random Effects** | | | |
| σ^2^ | 0.22 | | |
| τ_00_ _pop1_ | 0.18 | | |
| ICC | 0.45 | | |
| N _pop1_ | 51 | | |
| Observations | 1275 | | |
| Marginal R^2^ / Conditional R^2^ | 0.612 / 0.787 | | |
| AICc | 1871.562 | | |

| ***P. hermannsburgensis:* Microsatellites** | **response** | | |
| --- | --- | --- | --- |
| *Predictors* | *Estimates* | *CI* | *p* |
| (Intercept) | 7.12 | 7.04 – 7.21 | **<0.001** |
| Clay | 0.06 | 0.04 – 0.08 | **<0.001** |
| **Random Effects** | | | |
| σ^2^ | 0.10 | | |
| τ_00_ _pop1_ | 0.06 | | |
| ICC | 0.39 | | |
| N _pop1_ | 130 | | |
| Observations | 8385 | | |
| Marginal R^2^ / Conditional R^2^ | 0.025 / 0.407 | | |
| AICc | 4710.167 | | |
| ***P. hermannsburgensis:* SNPs** | **response** | | |
| *Predictors* | *Estimates* | *CI* | *p* |
| (Intercept) | 39.34 | 39.16 – 39.53 | **<0.001** |
| VRM | 0.33 | 0.29 – 0.37 | **<0.001** |
| **Random Effects** | | | |
| σ^2^ | 0.12 | | |
| τ_00_ _pop1_ | 0.29 | | |
| ICC | 0.70 | | |
| N _pop1_ | 130 | | |
| Observations | 8385 | | |
| Marginal R^2^ / Conditional R^2^ | 0.209 / 0.764 | | |
| AICc | 7091.639 | | |

**Appendix S7. MLPE model diagnostic plots.**

Diagnostic plots for the top-ranked linear mixed effects models, fit with the optimised single/composite surface as the predictor variable and the Euclidean genetic distance as the response variable (with a population level random effect to account for pairwise comparisons). We tested model performance by visualising Quantile-Quantile plots of model residuals (plot inset a) and scaled residuals based on a simulation approach using the ‘DHARMa’ package (plot inset b) (Hartig, 2022). We also visualised scatter plots of landscape distance against genetic distance (plot inset c), scatter plots of model residuals compared to landscape resistance (plot inset d), and histograms of model residuals (plot inset e).

The goodness-of-fit tests performed by ‘DHARMa’ were significant for all species and marker types (plot inset b). However, the p-value alone is not a good indicator of the extent to which residuals deviate from assumptions, since this is also influenced by the number of data points (which in our case is large due to the pairwise nature of the data). Given our dispersion parameters were less than 1.5 for models based on microsatellites, and less than 1.8 for models based on SNPs, we did not think it was necessary to account for overdispersion. Note that the slight skew in residuals for some models likely represents the fact that there are many more pairwise estimates for individuals that are geographically close in proximity, than those at the maximum geographical distance in our study region (also highlighted by the clustering of data points in inset plots c-d).

Where diagnostic plots show potential outlier residuals, we compared models with an alternative where outliers were removed (standardized residuals greater than 2.5 standard deviations) (Tremblay & Ransijn, 2020). In these cases, only a small proportion of datapoints were removed (less than 1-2%) and model estimates, significance and confidence intervals remained consistent (see Figure S7g for details). We therefore chose to proceed with the original models, to keep the datasets consistent with those used during the resistance surface optimisation procedure.

Plots are presented for each species and marker type below.


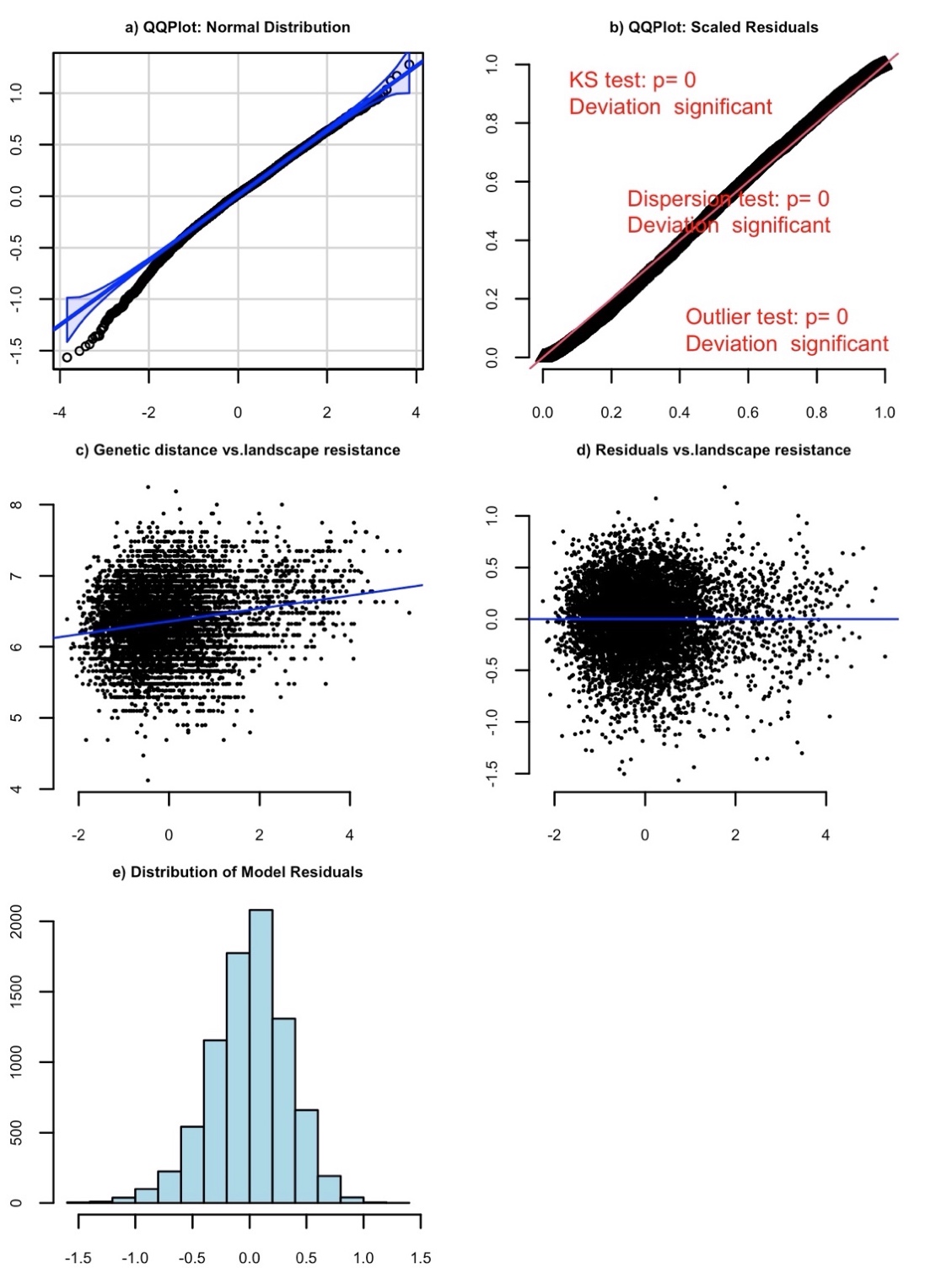


**Figure S7a.** Diagnostic plots for the *N. timealeyi* top-ranked microsatellite model. The model residuals have a slight left skew (a, e), although this skew appears minimal, and is not as prevalent in the simulated residuals (b), suggesting the model is a reasonable fit to the data. Furthermore, resistance distance increases linearly with increasing genetic distance (c) and there is no obvious systematic bias in the distribution of residuals (d).

| **Outliers Included** | **Outliers Removed** |
| --- | --- |
| 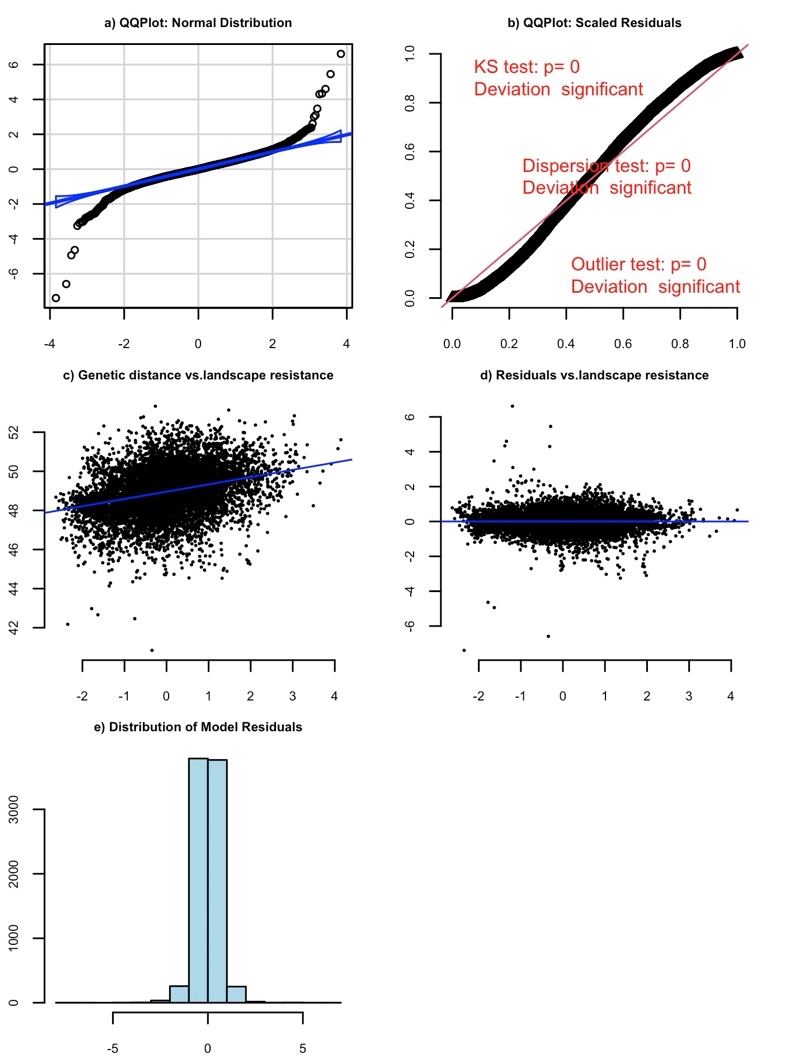 | 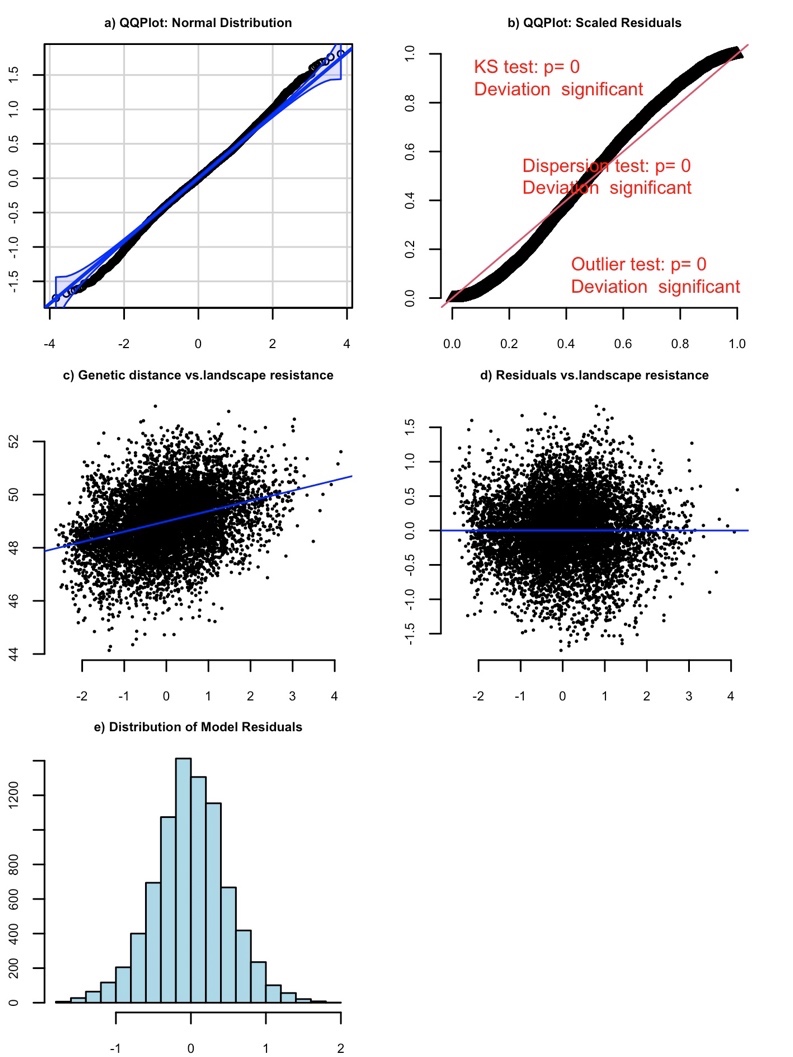 |

**Figure S7b.** Diagnostic plots for the *N. timealeyi* top-ranked SNP model, with and without outliers. After outliers were removed, the distribution of model residuals appears normal (a-b, e). Furthermore, resistance distance increases linearly with increasing genetic distance (c) and there is no obvious systematic bias in the distribution of residuals (d). See Figure S7g to see the difference between model estimates for these models.

| **Outliers Included** | **Outliers Removed** |
| --- | --- |
| 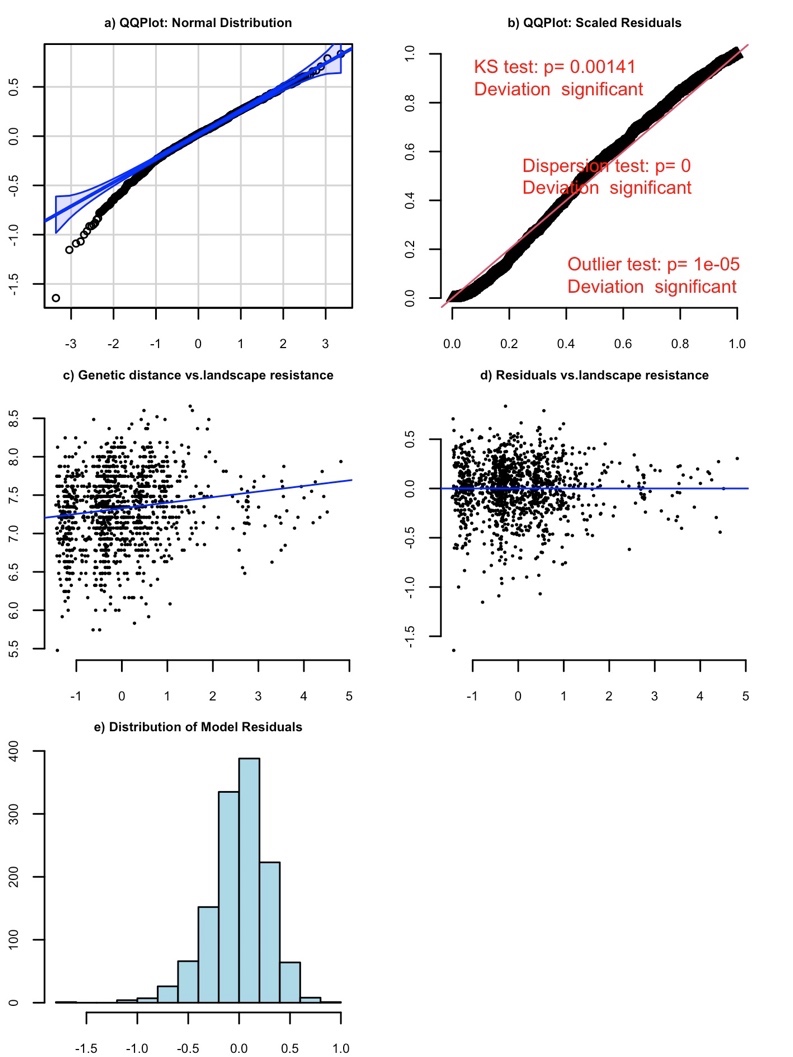 | 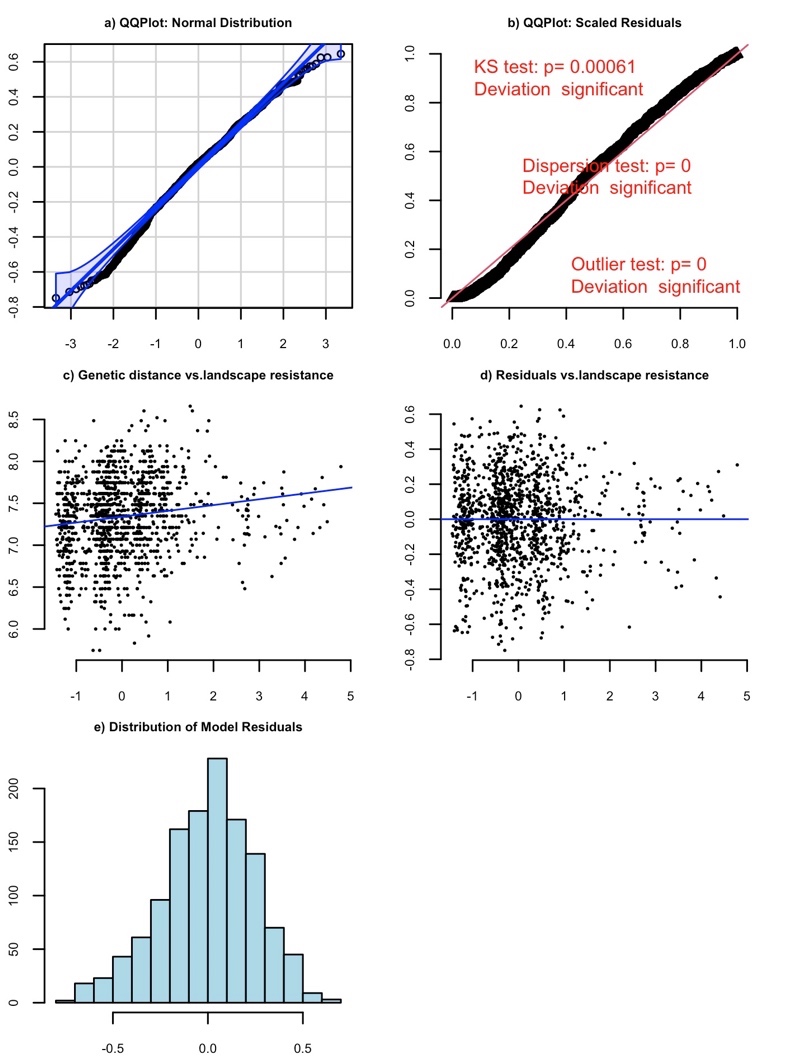 |

**Figure S7c.** Diagnostic plots for the *P. chapmani* top-ranked microsatellite model, with and without outliers. After outliers were removed, the distribution of model residuals appears normal (a-b, e). Furthermore, resistance distance increases linearly with increasing genetic distance (c) and there is no obvious systematic bias in the distribution of residuals (d). See Figure S7g to see the difference between model estimates for these models.

| **Outliers Included** | **Outliers Removed** |
| --- | --- |
| 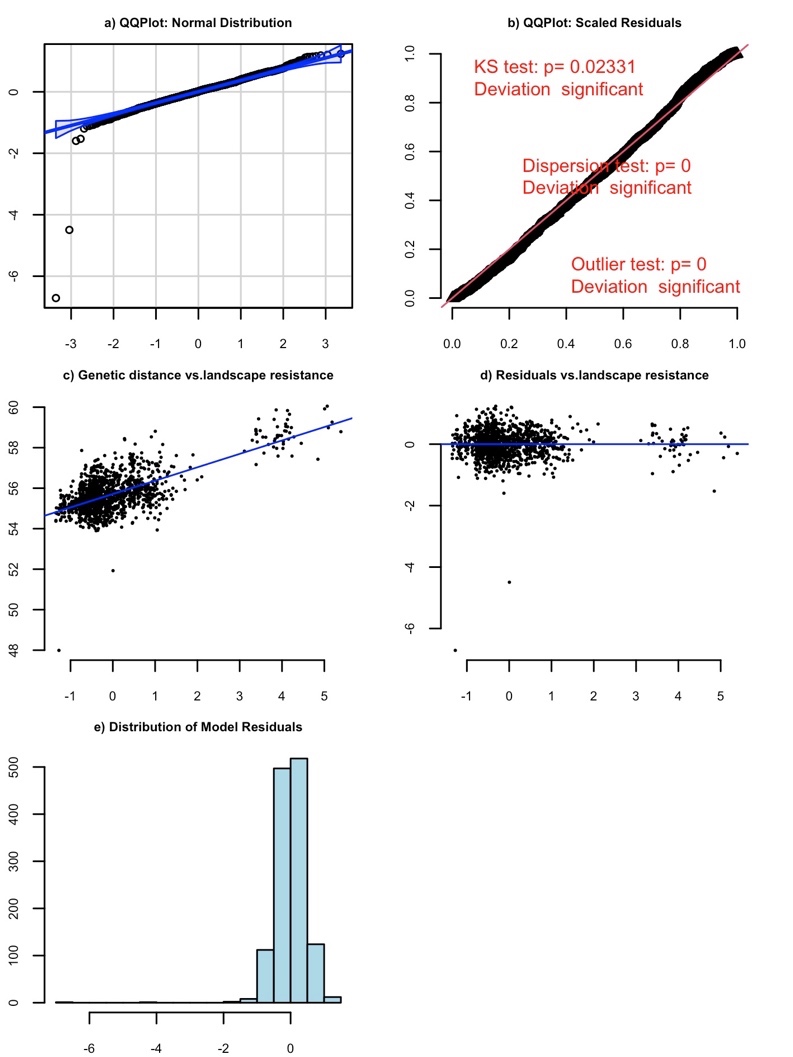 | 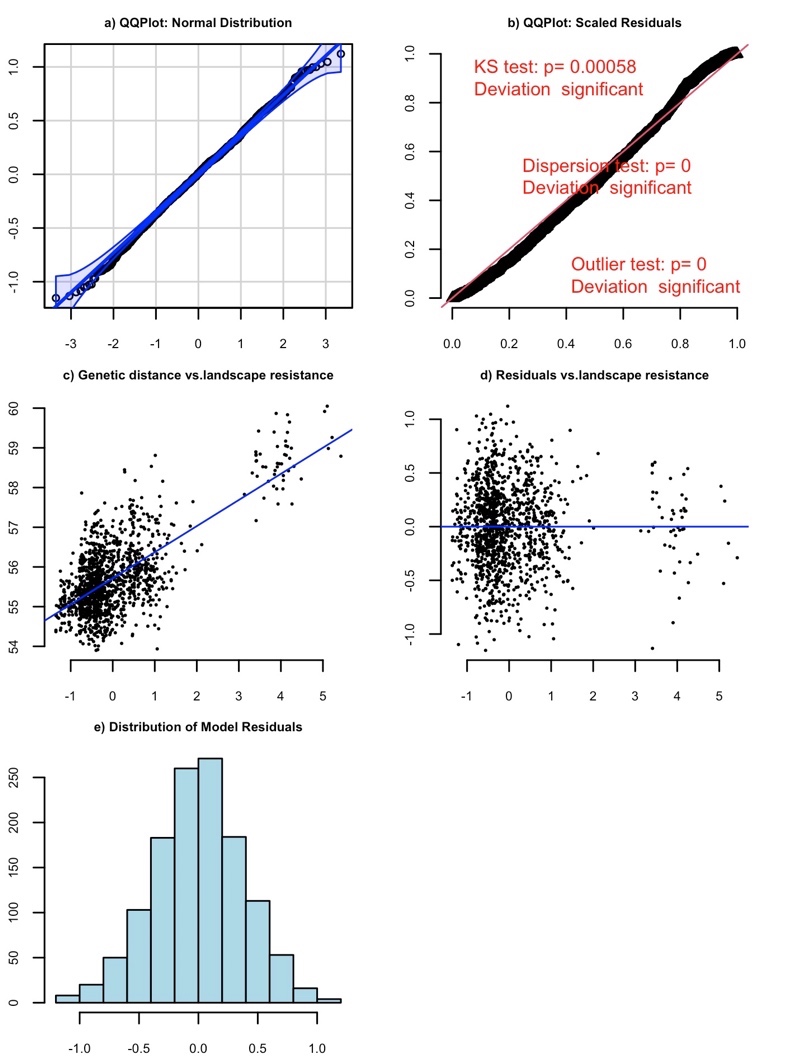 |

**Figure S7d.** Diagnostic plots for the *P. chapmani* top-ranked SNP model, with and without outliers. After outliers were removed, the distribution of model residuals appears normal (a-b, e). Furthermore, resistance distance increases linearly with increasing genetic distance (c) and there is no obvious systematic bias in the distribution of residuals (d). See Figure S7g to see the difference between model estimates for these models.

**
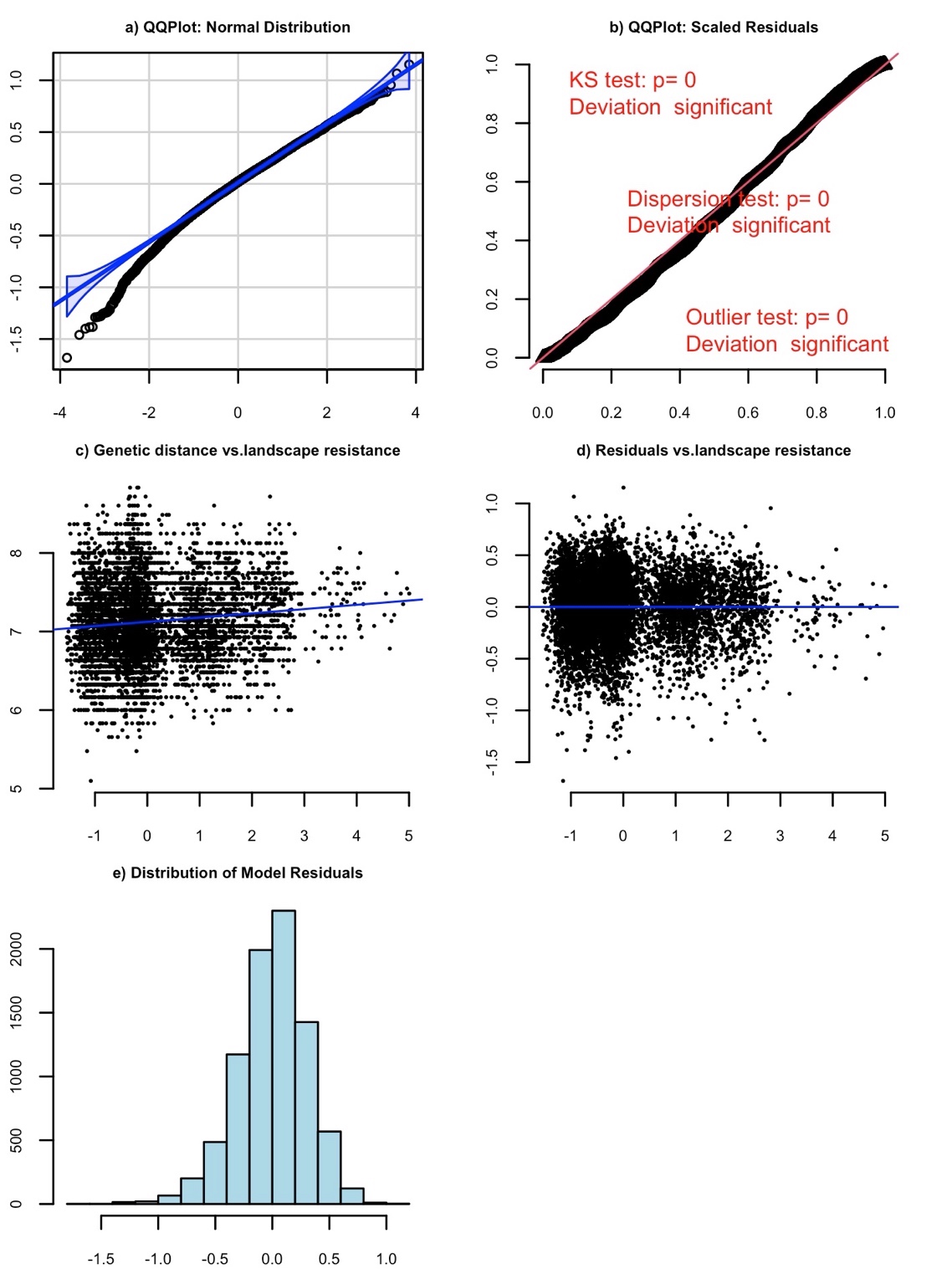
**

**Figure S7e.** Diagnostic plots for the *P. hermannsburgensis* top-ranked microsatellite model. The model residuals have a slight left skew (a, e), although this skew appears minimal, and is not as prevalent in the simulated residuals (b), suggesting the model is a reasonable fit to the data. Furthermore, resistance distance increases linearly with increasing genetic distance (although this trend is weak; c) and there is no obvious systematic bias in the distribution of residuals (d).

**
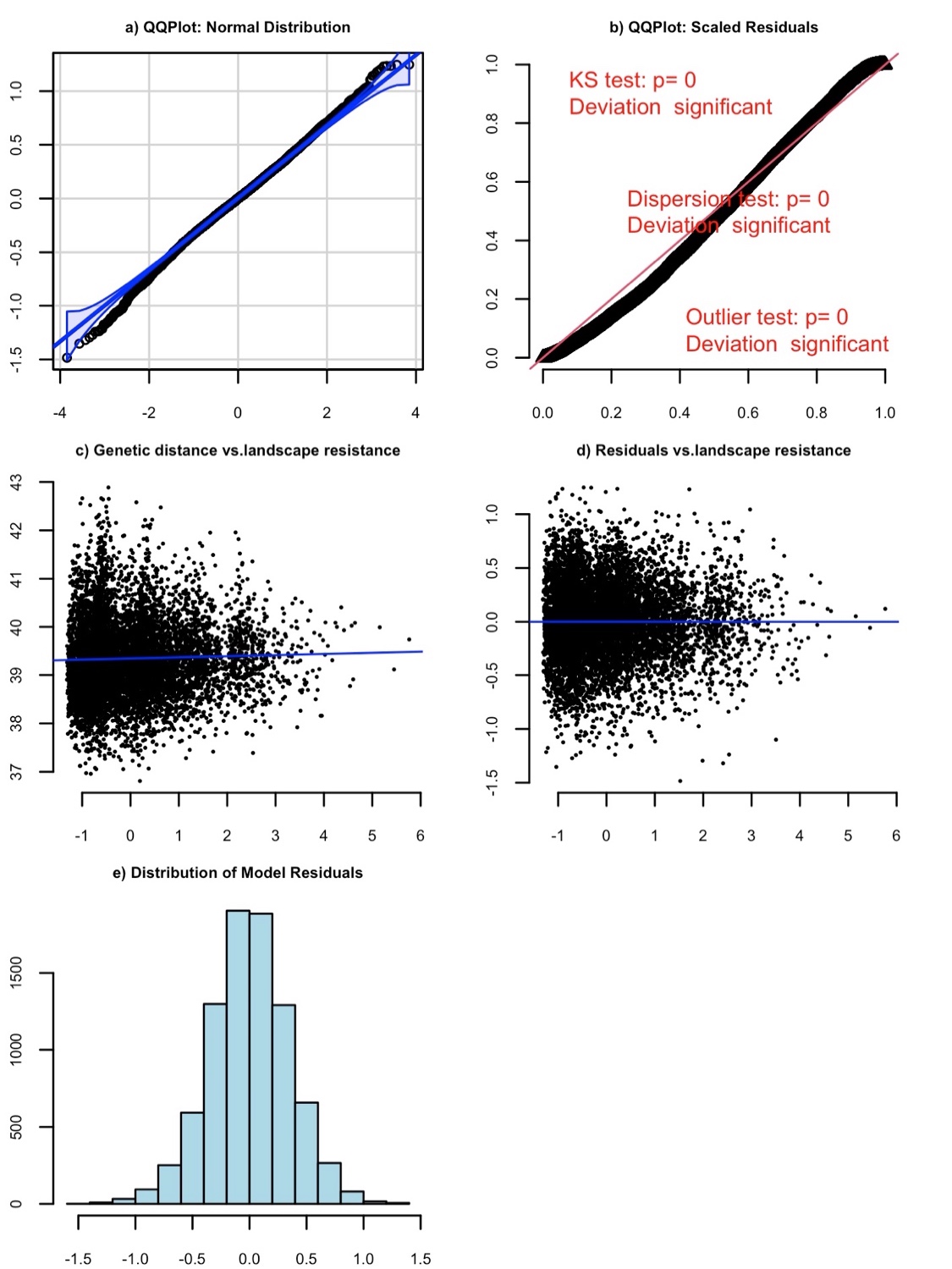
**

**Figure S7f.** Diagnostic plots for the *P. hermannsburgensis* top-ranked SNP model. The distribution of model residuals appears normal (a-b, e), suggesting the model is a reasonable fit to the data. Resistance distance shows a weak positive relationship with genetic distance (weak; c) and there is no obvious systematic bias in the distribution of residuals (d).


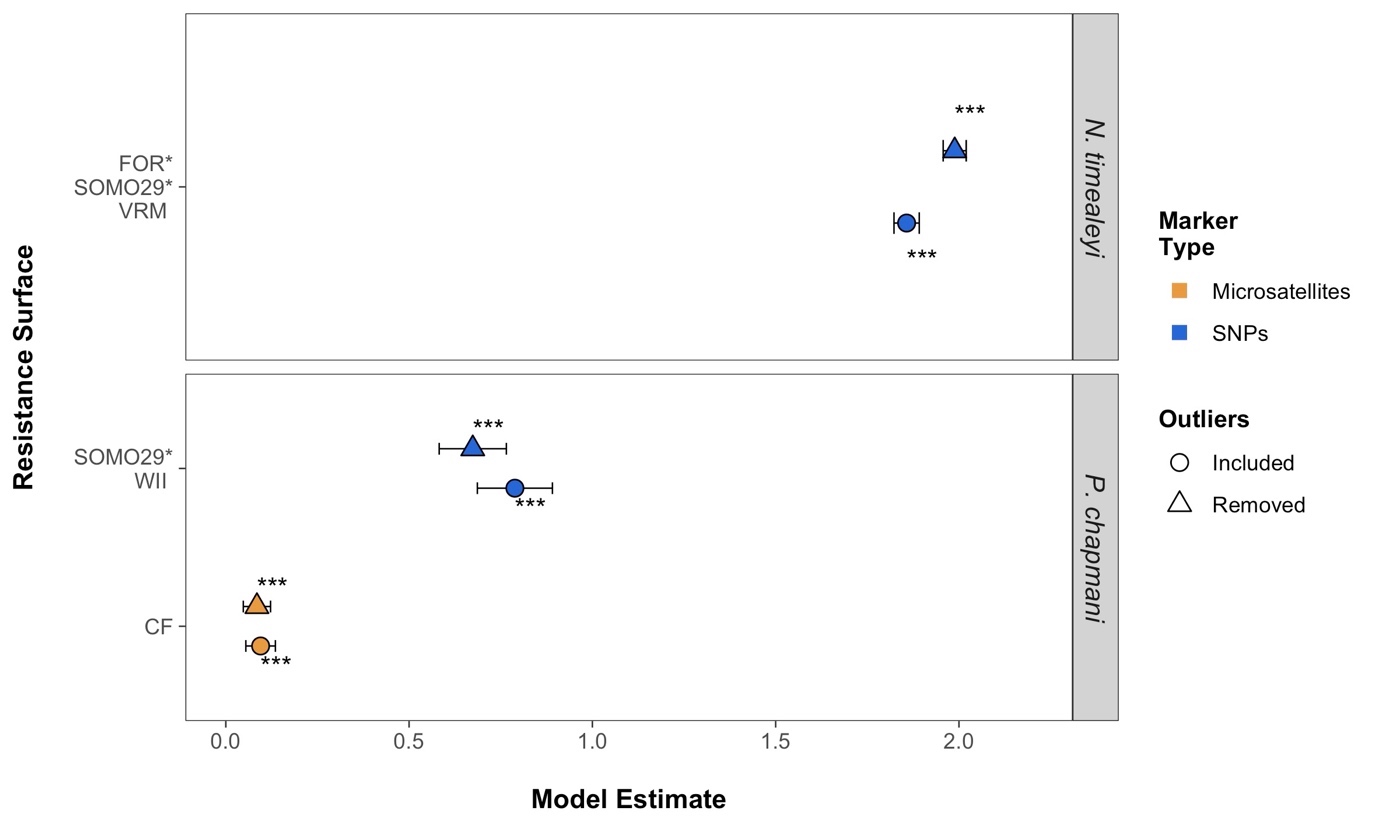


**Figure S7g.** Comparison of estimates (with confidence intervals and significance) between the top-ranked model and the alternative model where outliers were removed. Confidence intervals overlap for the original model and its alternative, for *P. chapmani* across the microsatellite and SNP datasets, with estimates and significance remaining consistent. While confidence intervals do not overlap for the alternative *N. timealeyi* models, the size of the confidence interval, standard error, and magnitude of estimate remained consistent (in relationship to the other species/marker types) and the difference was minor (1.86 with outliers versus 1.99 without outliers, with the original model giving a slightly more conservative estimate).

**Appendix S8. *ResistanceGA* bootstrap results.**

Full bootstrap results for single- and multi-surface optimised layers, for both marker types and all species, where Avg.: average over 1000 bootstrap iterations, AIC: Akaike Information Criterion, AICc: Akaike Information Criterion corrected for small sample size, weight: Akaike weight indicating the relative likelihood of each model, rank: model ranking over bootstrap iterations, R^2^m: marginal R^2^, LL: log-likelihood, RMSE: Root Mean Squared Error, n = number of bootstrap iterations where model ranked best, Top model (%): percentage of 1000 bootstrap iterations where top model, k: number of parameters.

***N. timealeyi SNP bootstrap results***

| **Surface** | **Avg. AIC** | **Avg. AICc** | **Avg. weight** | **Avg. rank** | **Avg. R^2^m** | **Avg. LL** | **Avg. RMSE** | **n** | **Top model (%)** | **k** |
| --- | --- | --- | --- | --- | --- | --- | --- | --- | --- | --- |
| SOMO29 * FOR * VRM | 8875.408 | 8877.996 | 0.452 | 2.796 | 0.633 | -4427.704 | 0.593 | 454 | 45.4 | 10 |
| SOMO29 * SPIN * VRM | 8876.217 | 8878.805 | 0.358 | 2.796 | 0.632 | -4428.108 | 0.594 | 355 | 35.5 | 10 |
| SOMO29 * WAT * FOR * VRM | 8915.227 | 8919.666 | 0.001 | 6.071 | 0.626 | -4444.613 | 0.596 | 1 | 0.1 | 13 |
| SOMO29 * WAT * SPIN * VRM | 8923.29 | 8927.729 | 0 | 7.255 | 0.633 | -4448.645 | 0.596 | 0 | 0 | 13 |
| SOMO29 * FOR * SPIN * VRM | 8947.915 | 8952.354 | 0 | 9.777 | 0.619 | -4460.957 | 0.599 | 0 | 0 | 13 |
| SOMO29 * VRM | 8981.353 | 8982.626 | 0 | 13.999 | 0.626 | -4483.677 | 0.601 | 0 | 0 | 7 |
| SOMO29 * DEM * VRM | 8982.228 | 8984.816 | 0 | 14.386 | 0.627 | -4481.114 | 0.601 | 0 | 0 | 10 |
| SOMO29 * Clay * FOR * VRM | 8983.266 | 8987.705 | 0 | 15.37 | 0.62 | -4478.633 | 0.602 | 0 | 0 | 13 |
| SOMO29 * DEM * FOR * VRM | 8987.696 | 8992.135 | 0 | 16.394 | 0.625 | -4480.848 | 0.601 | 0 | 0 | 13 |
| Clay * FOR * VRM | 8996.762 | 8999.35 | 0.048 | 17.202 | 0.634 | -4488.381 | 0.601 | 48 | 4.8 | 10 |
| SOMO29 * Clay * FOR | 9007.517 | 9010.105 | 0.098 | 17.681 | 0.624 | -4493.758 | 0.602 | 99 | 9.9 | 10 |
| SOMO29 * Clay * FOR * SPIN | 9017.842 | 9022.281 | 0 | 19.876 | 0.62 | -4495.921 | 0.603 | 0 | 0 | 13 |
| DEM * FOR * VRM | 9025.576 | 9028.164 | 0.003 | 21.445 | 0.635 | -4502.788 | 0.603 | 2 | 0.2 | 10 |
| SOMO29 * Clay * WAT * FOR | 9034.879 | 9039.318 | 0.001 | 22.397 | 0.6 | -4504.44 | 0.606 | 2 | 0.2 | 13 |
| DEM * FOR * SPIN * VRM | 9028.515 | 9032.955 | 0.002 | 23.016 | 0.636 | -4501.258 | 0.602 | 3 | 0.3 | 13 |
| SOMO29 * FOR | 9052.726 | 9053.998 | 0.036 | 24.264 | 0.612 | -4519.363 | 0.607 | 35 | 3.5 | 7 |
| Clay * VRM | 9039.825 | 9041.097 | 0 | 26.729 | 0.634 | -4512.912 | 0.604 | 0 | 0 | 7 |
| SOMO29 * DEM * FOR | 9062.592 | 9065.18 | 0 | 26.968 | 0.611 | -4521.296 | 0.607 | 0 | 0 | 10 |
| SOMO29 * FOR * SPIN | 9062.932 | 9065.52 | 0 | 27.653 | 0.611 | -4521.466 | 0.607 | 0 | 0 | 10 |
| SOMO29 * WAT * FOR | 9063.182 | 9065.77 | 0 | 27.831 | 0.611 | -4521.591 | 0.607 | 0 | 0 | 10 |
| DEM * VRM | 9046.177 | 9047.45 | 0 | 28.835 | 0.636 | -4516.088 | 0.604 | 0 | 0 | 7 |
| Clay * WAT * FOR * VRM | 9048.402 | 9052.841 | 0 | 29.514 | 0.633 | -4511.201 | 0.604 | 0 | 0 | 13 |
| FOR * VRM | 9050.367 | 9051.64 | 0 | 30.129 | 0.634 | -4518.183 | 0.605 | 0 | 0 | 7 |
| Clay * DEM * VRM | 9049.324 | 9051.912 | 0 | 30.349 | 0.636 | -4514.662 | 0.604 | 0 | 0 | 10 |
| SOMO29 * DEM * FOR * SPIN | 9071.728 | 9076.167 | 0 | 30.519 | 0.609 | -4522.864 | 0.608 | 0 | 0 | 13 |
| VRM | 9055.258 | 9055.697 | 0 | 31.222 | 0.631 | -4523.629 | 0.606 | 0 | 0 | 4 |
| SPIN * VRM | 9059.44 | 9060.713 | 0 | 31.886 | 0.625 | -4522.72 | 0.607 | 0 | 0 | 7 |
| SOMO29 * DEM * WAT * FOR | 9077.208 | 9081.647 | 0 | 32.176 | 0.61 | -4525.604 | 0.608 | 0 | 0 | 13 |
| Clay * SPIN * VRM | 9052.833 | 9055.421 | 0 | 32.74 | 0.634 | -4516.416 | 0.604 | 0 | 0 | 10 |
| WAT * FOR * VRM | 9054.151 | 9056.74 | 0 | 33.321 | 0.636 | -4517.076 | 0.604 | 0 | 0 | 10 |
| DEM * WAT * VRM | 9057.863 | 9060.451 | 0 | 33.706 | 0.633 | -4518.931 | 0.605 | 0 | 0 | 10 |
| SOMO29 * DEM * WAT * VRM | 9084.047 | 9088.486 | 0 | 33.983 | 0.592 | -4529.024 | 0.611 | 0 | 0 | 13 |
| Clay * FOR * SPIN * VRM | 9058.276 | 9062.715 | 0 | 34.002 | 0.631 | -4516.138 | 0.605 | 0 | 0 | 13 |
| Clay * WAT * VRM | 9063.012 | 9065.6 | 0 | 35.25 | 0.63 | -4521.506 | 0.606 | 0 | 0 | 10 |
| DEM * WAT * FOR * VRM | 9061.215 | 9065.654 | 0 | 35.903 | 0.634 | -4517.607 | 0.605 | 0 | 0 | 13 |
| WAT * SPIN * VRM | 9059.011 | 9061.599 | 0 | 36.087 | 0.635 | -4519.505 | 0.605 | 0 | 0 | 10 |
| SOMO29 * Clay | 9117.584 | 9118.856 | 0 | 36.941 | 0.59 | -4551.792 | 0.613 | 0 | 0 | 7 |
| WAT * VRM | 9071.465 | 9072.738 | 0 | 37.65 | 0.628 | -4528.732 | 0.607 | 0 | 0 | 7 |
| SOMO29 * Clay * WAT | 9121.191 | 9123.779 | 0 | 38.274 | 0.595 | -4550.595 | 0.613 | 0 | 0 | 10 |
| SOMO29 * Clay * VRM | 9103.242 | 9105.83 | 0 | 38.479 | 0.585 | -4541.621 | 0.613 | 0 | 0 | 10 |
| SOMO29 * Clay * SPIN | 9122.829 | 9125.417 | 0 | 39.392 | 0.597 | -4551.415 | 0.613 | 0 | 0 | 10 |
| SOMO29 * DEM * SPIN * VRM | 9109.84 | 9114.279 | 0 | 39.697 | 0.579 | -4541.92 | 0.614 | 0 | 0 | 13 |
| SOMO29 * Clay * DEM | 9123.485 | 9126.073 | 0 | 39.822 | 0.6 | -4551.742 | 0.612 | 0 | 0 | 10 |
| FOR * SPIN * VRM | 9068.366 | 9070.954 | 0 | 40.099 | 0.635 | -4524.183 | 0.605 | 0 | 0 | 10 |
| WAT * FOR * SPIN * VRM | 9074.462 | 9078.901 | 0 | 40.911 | 0.63 | -4524.231 | 0.606 | 0 | 0 | 13 |
| DEM * SPIN * VRM | 9077.315 | 9079.903 | 0 | 41.786 | 0.629 | -4528.657 | 0.607 | 0 | 0 | 10 |
| Clay * DEM * WAT * VRM | 9074.468 | 9078.907 | 0 | 41.92 | 0.631 | -4524.234 | 0.606 | 0 | 0 | 13 |
| SOMO29 * Clay * SPIN * VRM | 9105.217 | 9109.656 | 0 | 42.878 | 0.613 | -4539.609 | 0.61 | 0 | 0 | 13 |
| SOMO29 * Clay * WAT * VRM | 9138.404 | 9142.843 | 0 | 43.727 | 0.587 | -4556.202 | 0.614 | 0 | 0 | 13 |
| SOMO29 * Clay * DEM * FOR | 9151.353 | 9155.792 | 0.001 | 44.543 | 0.581 | -4562.676 | 0.615 | 1 | 0.1 | 13 |
| Clay * DEM * SPIN * VRM | 9106.312 | 9110.751 | 0 | 44.934 | 0.618 | -4540.156 | 0.61 | 0 | 0 | 13 |
| DEM * WAT * SPIN * VRM | 9106.709 | 9111.148 | 0 | 46.312 | 0.621 | -4540.354 | 0.609 | 0 | 0 | 13 |
| SOMO29 * Clay * DEM * SPIN | 9151.148 | 9155.587 | 0 | 47.19 | 0.588 | -4562.574 | 0.615 | 0 | 0 | 13 |
| SOMO29 | 9173.077 | 9173.517 | 0 | 48.874 | 0.547 | -4582.539 | 0.62 | 0 | 0 | 4 |
| SOMO29 * WAT * VRM | 9151.906 | 9154.495 | 0 | 48.888 | 0.569 | -4565.953 | 0.617 | 0 | 0 | 10 |
| SOMO29 * Clay * DEM * VRM | 9125.756 | 9130.195 | 0 | 49.712 | 0.617 | -4549.878 | 0.611 | 0 | 0 | 13 |
| SOMO29 * Clay * DEM * WAT | 9172.038 | 9176.477 | 0 | 50.222 | 0.583 | -4573.019 | 0.617 | 0 | 0 | 13 |
| SOMO29 * WAT | 9180.093 | 9181.366 | 0 | 51.292 | 0.547 | -4583.047 | 0.62 | 0 | 0 | 7 |
| SOMO29 * SPIN | 9180.099 | 9181.371 | 0 | 52.239 | 0.547 | -4583.049 | 0.62 | 0 | 0 | 7 |
| SOMO29 * DEM | 9180.102 | 9181.374 | 0 | 53.15 | 0.547 | -4583.051 | 0.62 | 0 | 0 | 7 |
| SOMO29 * WAT * SPIN | 9184.432 | 9187.02 | 0 | 54.243 | 0.569 | -4582.216 | 0.619 | 0 | 0 | 10 |
| SOMO29 * DEM * WAT | 9187.156 | 9189.744 | 0 | 55.727 | 0.546 | -4583.578 | 0.62 | 0 | 0 | 10 |
| SOMO29 * DEM * SPIN | 9187.174 | 9189.762 | 0 | 56.492 | 0.546 | -4583.587 | 0.62 | 0 | 0 | 10 |
| SOMO29 * DEM * WAT * SPIN | 9196.496 | 9200.935 | 0 | 58.192 | 0.57 | -4585.248 | 0.619 | 0 | 0 | 13 |
| Clay * DEM * FOR * SPIN | 9202.913 | 9207.352 | 0 | 58.609 | 0.648 | -4588.457 | 0.613 | 0 | 0 | 13 |
| Clay * WAT * SPIN * VRM | 9193.498 | 9197.937 | 0 | 58.767 | 0.598 | -4583.749 | 0.617 | 0 | 0 | 13 |
| SOMO29 * Clay * WAT * SPIN | 9226.203 | 9230.642 | 0 | 62.678 | 0.52 | -4600.101 | 0.624 | 0 | 0 | 13 |
| Clay * DEM * FOR | 9257.849 | 9260.437 | 0 | 63.641 | 0.635 | -4618.924 | 0.616 | 0 | 0 | 10 |
| Clay * FOR | 9284.367 | 9285.64 | 0 | 66.169 | 0.637 | -4635.184 | 0.618 | 0 | 0 | 7 |
| Clay * DEM * FOR * VRM | 9253.589 | 9258.028 | 0 | 66.174 | 0.587 | -4613.794 | 0.622 | 0 | 0 | 13 |
| SOMO29 * WAT * FOR * SPIN | 9256.256 | 9260.695 | 0 | 66.99 | 0.52 | -4615.128 | 0.626 | 0 | 0 | 13 |
| Clay * FOR * SPIN | 9290.062 | 9292.651 | 0 | 67.881 | 0.636 | -4635.031 | 0.618 | 0 | 0 | 10 |
| Clay * DEM * WAT * FOR | 9291.398 | 9295.837 | 0 | 68.142 | 0.626 | -4632.699 | 0.62 | 0 | 0 | 13 |
| Clay * WAT * FOR | 9315.263 | 9317.851 | 0 | 71.124 | 0.623 | -4647.631 | 0.621 | 0 | 0 | 10 |
| Clay * DEM * WAT * SPIN | 9330.437 | 9334.876 | 0 | 73.173 | 0.612 | -4652.219 | 0.625 | 0 | 0 | 13 |
| Clay * DEM * SPIN | 9336.208 | 9338.796 | 0 | 74.039 | 0.628 | -4658.104 | 0.624 | 0 | 0 | 10 |
| Clay * SPIN | 9376.635 | 9377.907 | 0 | 77.165 | 0.625 | -4681.317 | 0.627 | 0 | 0 | 7 |
| Clay * WAT * FOR * SPIN | 9367.952 | 9372.391 | 0 | 77.219 | 0.593 | -4670.976 | 0.628 | 0 | 0 | 13 |
| Clay | 9381.411 | 9381.85 | 0 | 78.797 | 0.611 | -4686.705 | 0.628 | 0 | 0 | 4 |
| Clay * WAT | 9385.558 | 9386.83 | 0 | 79.572 | 0.612 | -4685.779 | 0.628 | 0 | 0 | 7 |
| Clay * DEM | 9387.842 | 9389.115 | 0 | 80.755 | 0.613 | -4686.921 | 0.628 | 0 | 0 | 7 |
| DEM * FOR * SPIN | 9426.059 | 9428.647 | 0 | 81.415 | 0.627 | -4703.03 | 0.632 | 0 | 0 | 10 |
| Clay * WAT * SPIN | 9392.87 | 9395.458 | 0 | 81.426 | 0.613 | -4686.435 | 0.628 | 0 | 0 | 10 |
| Clay * DEM * WAT | 9391.556 | 9394.144 | 0 | 81.549 | 0.612 | -4685.778 | 0.628 | 0 | 0 | 10 |
| DEM * WAT * FOR * SPIN | 9439.823 | 9444.262 | 0 | 82.944 | 0.581 | -4706.911 | 0.637 | 0 | 0 | 13 |
| WAT * FOR * SPIN | 9438.714 | 9441.302 | 0 | 82.96 | 0.624 | -4709.357 | 0.633 | 0 | 0 | 10 |
| FOR * SPIN | 9449.553 | 9450.826 | 0 | 83.874 | 0.636 | -4717.777 | 0.632 | 0 | 0 | 7 |
| DEM * WAT * SPIN | 9530.615 | 9533.203 | 0 | 88.351 | 0.558 | -4755.307 | 0.645 | 0 | 0 | 10 |
| DEM * FOR | 9556.742 | 9558.015 | 0 | 88.849 | 0.618 | -4771.371 | 0.64 | 0 | 0 | 7 |
| WAT * SPIN | 9554.355 | 9555.628 | 0 | 90.035 | 0.583 | -4770.178 | 0.645 | 0 | 0 | 7 |
| DEM * WAT * FOR | 9566.766 | 9569.355 | 0 | 90.219 | 0.62 | -4773.383 | 0.639 | 0 | 0 | 10 |
| DEM * SPIN | 9599.923 | 9601.195 | 0 | 91.882 | 0.638 | -4792.961 | 0.641 | 0 | 0 | 7 |
| WAT * FOR | 9622.616 | 9623.889 | 0 | 93.173 | 0.599 | -4804.308 | 0.646 | 0 | 0 | 7 |
| FOR | 9626.861 | 9627.3 | 0 | 93.627 | 0.592 | -4809.43 | 0.647 | 0 | 0 | 4 |
| DEM * WAT | 9653.188 | 9654.46 | 0 | 94.329 | 0.489 | -4819.594 | 0.655 | 0 | 0 | 7 |
| SPIN | 9675.409 | 9675.848 | 0 | 95.224 | 0.426 | -4833.704 | 0.66 | 0 | 0 | 4 |
| DEM | 9694.616 | 9695.056 | 0 | 96.642 | 0.471 | -4843.308 | 0.66 | 0 | 0 | 4 |
| WAT | 9703.334 | 9703.774 | 0 | 97.132 | 0.432 | -4847.667 | 0.661 | 0 | 0 | 4 |
| IBD | 9717.829 | 9717.958 | 0 | 98.36 | 0.425 | -4856.915 | 0.663 | 0 | 0 | 2 |

***N. timealeyi microsatellite bootstrap results***

| **Surface** | **Avg. AIC** | **Avg. AICc** | **Avg. weight** | **Avg. rank** | **Avg. R^2^m** | **Avg. LL** | **Avg. RMSE** | **n** | **Top model (%)** | **k** |
| --- | --- | --- | --- | --- | --- | --- | --- | --- | --- | --- |
| CF | 3460.573 | 3461.012 | 0.904 | 1.038 | 0.105 | -1726.286 | 0.336 | 972 | 97.2 | 4 |
| SOMO29 * CF | 3466.593 | 3467.865 | 0.029 | 2.287 | 0.106 | -1726.296 | 0.336 | 0 | 0 | 7 |
| SOMO29 | 3472.663 | 3473.103 | 0.029 | 3.215 | 0.028 | -1732.332 | 0.336 | 10 | 1 | 4 |
| IBD | 3473.666 | 3473.795 | 0.037 | 3.46 | 0.024 | -1734.833 | 0.337 | 18 | 1.8 | 2 |

***P. chapmani SNP bootstrap results***

| **Surface** | **Avg. AIC** | **Avg. AICc** | **Avg. weight** | **Avg. rank** | **Avg. R^2^m** | **Avg. LL** | **Avg. RMSE** | **n** | **Top model (%)** | **k** |
| --- | --- | --- | --- | --- | --- | --- | --- | --- | --- | --- |
| SOMO29 * WII | 1050.393 | 1054.127 | 0.426 | 5.299 | 0.566 | -518.197 | 0.451 | 444 | 44.4 | 7 |
| SOMO29 * SPIN | 1057.224 | 1060.958 | 0.197 | 5.387 | 0.331 | -521.612 | 0.445 | 225 | 22.5 | 7 |
| SOMO29 * WAT * SPIN | 1063.363 | 1071.511 | 0.001 | 9.531 | 0.352 | -521.682 | 0.445 | 0 | 0 | 10 |
| SOMO29 * SPIN * WII | 1063.546 | 1071.694 | 0.001 | 10.278 | 0.351 | -521.773 | 0.445 | 0 | 0 | 10 |
| SOMO29 * SPIN * VRM | 1063.939 | 1072.087 | 0.001 | 11.045 | 0.37 | -521.97 | 0.445 | 0 | 0 | 10 |
| DEM * VRM * WII | 1062.756 | 1070.904 | 0.002 | 12.316 | 0.516 | -521.378 | 0.451 | 6 | 0.6 | 10 |
| VRM * WII | 1062.431 | 1066.164 | 0.155 | 13.522 | 0.516 | -524.215 | 0.454 | 163 | 16.3 | 7 |
| SOMO29 * VRM | 1069.676 | 1073.409 | 0.008 | 14.788 | 0.266 | -527.838 | 0.45 | 8 | 0.8 | 7 |
| DEM | 1071.537 | 1072.749 | 0.005 | 16.609 | 0.368 | -531.769 | 0.448 | 1 | 0.1 | 4 |
| WAT * WII | 1066.691 | 1070.424 | 0.037 | 17.322 | 0.419 | -526.345 | 0.455 | 26 | 2.6 | 7 |
| SOMO29 * WAT * SPIN * WII | 1071.036 | 1086.203 | 0 | 18.236 | 0.3 | -522.518 | 0.446 | 0 | 0 | 13 |
| SOMO29 * DEM | 1071.804 | 1075.537 | 0.003 | 18.352 | 0.367 | -528.902 | 0.447 | 2 | 0.2 | 7 |
| SOMO29 * DEM * SPIN * VRM | 1070.534 | 1085.7 | 0 | 18.778 | 0.365 | -522.267 | 0.445 | 0 | 0 | 13 |
| SOMO29 | 1070.953 | 1072.165 | 0.039 | 19.029 | 0.33 | -531.476 | 0.449 | 20 | 2 | 4 |
| SOMO29 * WAT * SPIN * VRM | 1071.198 | 1086.365 | 0 | 19.507 | 0.325 | -522.599 | 0.445 | 0 | 0 | 13 |
| VRM | 1072.885 | 1074.097 | 0.027 | 20.839 | 0.221 | -532.442 | 0.453 | 27 | 2.7 | 4 |
| SPIN * WII | 1069.279 | 1073.012 | 0.051 | 20.942 | 0.41 | -527.639 | 0.456 | 41 | 4.1 | 7 |
| WII | 1074.662 | 1075.874 | 0.003 | 24.476 | 0.222 | -533.331 | 0.456 | 1 | 0.1 | 4 |
| DEM * WAT | 1077.483 | 1081.216 | 0 | 25.516 | 0.366 | -531.741 | 0.448 | 0 | 0 | 7 |
| DEM * VRM | 1077.494 | 1081.227 | 0 | 25.953 | 0.366 | -531.747 | 0.448 | 0 | 0 | 7 |
| DEM * WII | 1077.495 | 1081.228 | 0 | 26.383 | 0.366 | -531.747 | 0.448 | 0 | 0 | 7 |
| DEM * SPIN | 1077.502 | 1081.235 | 0 | 26.82 | 0.366 | -531.751 | 0.448 | 0 | 0 | 7 |
| WAT * SPIN * WII | 1074.871 | 1083.02 | 0 | 27.134 | 0.422 | -527.436 | 0.457 | 0 | 0 | 10 |
| SOMO29 * WAT | 1076.44 | 1080.173 | 0 | 27.373 | 0.321 | -531.22 | 0.449 | 0 | 0 | 7 |
| SPIN * VRM * WII | 1076.354 | 1084.502 | 0 | 27.649 | 0.326 | -528.177 | 0.456 | 0 | 0 | 10 |
| SOMO29 * DEM * WAT | 1077.81 | 1085.958 | 0 | 28.098 | 0.295 | -528.905 | 0.449 | 0 | 0 | 10 |
| SPIN * VRM | 1077.91 | 1081.643 | 0.001 | 28.127 | 0.278 | -531.955 | 0.453 | 0 | 0 | 7 |
| SOMO29 * DEM * SPIN * WII | 1078.057 | 1093.223 | 0 | 28.41 | 0.195 | -526.028 | 0.448 | 0 | 0 | 13 |
| WAT | 1078.831 | 1080.044 | 0.001 | 28.931 | 0.19 | -535.416 | 0.452 | 0 | 0 | 4 |
| SOMO29 * DEM * WII | 1077.836 | 1085.985 | 0 | 29.157 | 0.366 | -528.918 | 0.447 | 0 | 0 | 10 |
| SOMO29 * DEM * VRM | 1077.864 | 1086.012 | 0 | 29.259 | 0.366 | -528.932 | 0.447 | 0 | 0 | 10 |
| WAT * VRM | 1078.907 | 1082.641 | 0 | 29.6 | 0.237 | -532.454 | 0.453 | 0 | 0 | 7 |
| SOMO29 * SPIN * VRM * WII | 1078.569 | 1093.736 | 0 | 29.849 | 0.187 | -526.284 | 0.449 | 0 | 0 | 13 |
| IBD | 1078.934 | 1079.277 | 0.035 | 29.886 | 0.07 | -537.467 | 0.456 | 30 | 3 | 2 |
| SPIN | 1080.624 | 1081.836 | 0.007 | 31.502 | 0.107 | -536.312 | 0.454 | 6 | 0.6 | 4 |
| SOMO29 * DEM * WAT * SPIN | 1079.182 | 1094.348 | 0 | 31.634 | 0.211 | -526.591 | 0.448 | 0 | 0 | 13 |
| SOMO29 * WAT * WII | 1078.676 | 1086.824 | 0 | 31.878 | 0.335 | -529.338 | 0.449 | 0 | 0 | 10 |
| SOMO29 * VRM * WII | 1079.39 | 1087.538 | 0 | 32.398 | 0.271 | -529.695 | 0.45 | 0 | 0 | 10 |
| WAT * SPIN | 1081.281 | 1085.015 | 0 | 32.859 | 0.198 | -533.641 | 0.452 | 0 | 0 | 7 |
| DEM * SPIN * WII | 1082.405 | 1090.554 | 0 | 35.598 | 0.348 | -531.203 | 0.448 | 0 | 0 | 10 |
| DEM * SPIN * VRM | 1082.933 | 1091.081 | 0 | 37.486 | 0.342 | -531.467 | 0.448 | 0 | 0 | 10 |
| SOMO29 * WAT * VRM | 1082.14 | 1090.288 | 0 | 37.715 | 0.324 | -531.07 | 0.449 | 0 | 0 | 10 |
| SOMO29 * DEM * SPIN | 1083.481 | 1091.629 | 0 | 38.46 | 0.365 | -531.74 | 0.448 | 0 | 0 | 10 |
| DEM * WAT * SPIN | 1083.505 | 1091.653 | 0 | 39.028 | 0.366 | -531.753 | 0.448 | 0 | 0 | 10 |
| DEM * WAT * WII | 1083.511 | 1091.659 | 0 | 39.466 | 0.366 | -531.755 | 0.448 | 0 | 0 | 10 |
| DEM * WAT * VRM | 1083.516 | 1091.664 | 0 | 39.882 | 0.366 | -531.758 | 0.448 | 0 | 0 | 10 |
| SOMO29 * DEM * WAT * VRM | 1083.438 | 1098.604 | 0 | 39.98 | 0.322 | -528.719 | 0.448 | 0 | 0 | 13 |
| SOMO29 * DEM * WAT * WII | 1083.98 | 1099.147 | 0 | 40.698 | 0.369 | -528.99 | 0.447 | 0 | 0 | 13 |
| SOMO29 * DEM * VRM * WII | 1084.414 | 1099.581 | 0 | 41.846 | 0.261 | -529.207 | 0.449 | 0 | 0 | 13 |
| WAT * SPIN * VRM | 1087.49 | 1095.638 | 0 | 41.974 | 0.177 | -533.745 | 0.452 | 0 | 0 | 10 |
| SOMO29 * WAT * VRM * WII | 1086.778 | 1101.945 | 0 | 44.777 | 0.293 | -530.389 | 0.45 | 0 | 0 | 13 |
| WAT * VRM * WII | 1089.676 | 1097.824 | 0 | 44.827 | 0.129 | -534.838 | 0.453 | 0 | 0 | 10 |
| WAT * SPIN * VRM * WII | 1094.924 | 1110.09 | 0 | 48.736 | 0.132 | -534.462 | 0.454 | 0 | 0 | 13 |
| DEM * WAT * SPIN * WII | 1089.19 | 1104.357 | 0 | 48.928 | 0.314 | -531.595 | 0.449 | 0 | 0 | 13 |
| DEM * SPIN * VRM * WII | 1089.516 | 1104.683 | 0 | 49.199 | 0.366 | -531.758 | 0.448 | 0 | 0 | 13 |
| DEM * WAT * VRM * WII | 1089.648 | 1104.815 | 0 | 49.833 | 0.37 | -531.824 | 0.448 | 0 | 0 | 13 |
| DEM * WAT * SPIN * VRM | 1089.761 | 1104.927 | 0 | 49.895 | 0.352 | -531.88 | 0.448 | 0 | 0 | 13 |

***P. chapmani microsatellite bootstrap results***

| **Surface** | **Avg. AIC** | **Avg. AICc** | **Avg. weight** | **Avg. rank** | **Avg. R^2^m** | **Avg. LL** | **Avg. RMSE** | **n** | **Top model (%)** | **k** |
| --- | --- | --- | --- | --- | --- | --- | --- | --- | --- | --- |
| CF | -22212.071 | -22210.859 | 1 | 1 | 1 | 11110.036 | 0 | 1000 | 100 | 4 |
| IBD | 6060.055 | 6060.398 | 0 | 2 | 0.323 | -3028.028 | 15.025 | 0 | 0 | 2 |

***P. hermannsburgensis SNP bootstrap results***

| **Surface** | **Avg. AIC** | **Avg. AICc** | **Avg. weight** | **Avg. rank** | **Avg. R^2^m** | **Avg. LL** | **Avg. RMSE** | **n** | **Top model (%)** | **k** |
| --- | --- | --- | --- | --- | --- | --- | --- | --- | --- | --- |
| VRM | 4042.475 | 4042.91 | 0.729 | 1.441 | 0.199 | -2017.238 | 0.349 | 926 | 92.6 | 4 |
| FF * VRM | 4048.386 | 4049.644 | 0.025 | 4.089 | 0.196 | -2017.193 | 0.349 | 0 | 0 | 7 |
| SOMO31 * VRM | 4048.396 | 4049.654 | 0.025 | 6.015 | 0.196 | -2017.198 | 0.349 | 0 | 0 | 7 |
| SPIN * VRM | 4048.393 | 4049.652 | 0.025 | 6.03 | 0.196 | -2017.197 | 0.349 | 0 | 0 | 7 |
| Silt * VRM | 4048.396 | 4049.654 | 0.025 | 6.508 | 0.196 | -2017.198 | 0.349 | 0 | 0 | 7 |
| VRM * WAT | 4048.398 | 4049.656 | 0.025 | 7.168 | 0.196 | -2017.199 | 0.349 | 0 | 0 | 7 |
| VRM * WII | 4048.399 | 4049.657 | 0.025 | 7.368 | 0.196 | -2017.199 | 0.349 | 0 | 0 | 7 |
| DEM * VRM | 4048.402 | 4049.661 | 0.025 | 7.567 | 0.196 | -2017.201 | 0.349 | 0 | 0 | 7 |
| FF * VRM * WII | 4053.775 | 4056.333 | 0.001 | 16.564 | 0.179 | -2016.887 | 0.349 | 0 | 0 | 10 |
| DEM * FF * VRM | 4054.232 | 4056.79 | 0.001 | 18.397 | 0.188 | -2017.116 | 0.349 | 0 | 0 | 10 |
| FF * Silt * VRM | 4054.232 | 4056.791 | 0.001 | 18.604 | 0.188 | -2017.116 | 0.349 | 0 | 0 | 10 |
| FF * SOMO31 * VRM | 4054.228 | 4056.786 | 0.001 | 19.006 | 0.188 | -2017.114 | 0.349 | 0 | 0 | 10 |
| SOMO31 * SPIN * VRM | 4054.187 | 4056.745 | 0.001 | 20.528 | 0.184 | -2017.094 | 0.349 | 0 | 0 | 10 |
| VRM * WAT * WII | 4054.711 | 4057.269 | 0.001 | 21.589 | 0.156 | -2017.356 | 0.349 | 0 | 0 | 10 |
| DEM * SPIN * VRM | 4054.237 | 4056.795 | 0.001 | 21.748 | 0.188 | -2017.118 | 0.349 | 0 | 0 | 10 |
| FF * VRM * WAT | 4054.24 | 4056.798 | 0.001 | 22.123 | 0.188 | -2017.12 | 0.349 | 0 | 0 | 10 |
| Silt * SOMO31 * VRM | 4054.24 | 4056.798 | 0.001 | 22.263 | 0.188 | -2017.12 | 0.349 | 0 | 0 | 10 |
| SOMO31 * VRM * WAT | 4054.805 | 4057.363 | 0.001 | 22.274 | 0.154 | -2017.402 | 0.349 | 0 | 0 | 10 |
| DEM * VRM * WAT | 4054.239 | 4056.798 | 0.001 | 22.433 | 0.188 | -2017.12 | 0.349 | 0 | 0 | 10 |
| DEM * SOMO31 * VRM | 4054.24 | 4056.798 | 0.001 | 22.49 | 0.188 | -2017.12 | 0.349 | 0 | 0 | 10 |
| FF * SPIN * VRM | 4054.902 | 4057.46 | 0.001 | 22.972 | 0.156 | -2017.451 | 0.349 | 0 | 0 | 10 |
| SOMO31 * VRM * WII | 4054.242 | 4056.801 | 0.001 | 23.222 | 0.188 | -2017.121 | 0.349 | 0 | 0 | 10 |
| Silt * SPIN * VRM | 4054.241 | 4056.799 | 0.001 | 23.326 | 0.188 | -2017.12 | 0.349 | 0 | 0 | 10 |
| SPIN * VRM * WII | 4054.241 | 4056.799 | 0.001 | 23.537 | 0.188 | -2017.12 | 0.349 | 0 | 0 | 10 |
| DEM * Silt * VRM | 4054.471 | 4057.029 | 0.001 | 23.873 | 0.177 | -2017.235 | 0.349 | 0 | 0 | 10 |
| DEM * VRM * WII | 4054.243 | 4056.802 | 0.001 | 24.161 | 0.188 | -2017.122 | 0.349 | 0 | 0 | 10 |
| Silt * VRM * WII | 4054.242 | 4056.8 | 0.001 | 24.268 | 0.188 | -2017.121 | 0.349 | 0 | 0 | 10 |
| SPIN * VRM * WAT | 4054.247 | 4056.805 | 0.001 | 24.298 | 0.188 | -2017.123 | 0.349 | 0 | 0 | 10 |
| Silt * VRM * WAT | 4055.373 | 4057.931 | 0.001 | 25.062 | 0.153 | -2017.686 | 0.349 | 0 | 0 | 10 |
| DEM * Silt * VRM * WII | 4060.062 | 4064.447 | 0 | 43.825 | 0.164 | -2017.031 | 0.349 | 0 | 0 | 13 |
| FF * SPIN * VRM * WAT | 4060.184 | 4064.569 | 0 | 45.982 | 0.18 | -2017.092 | 0.349 | 0 | 0 | 13 |
| SOMO31 * SPIN * VRM * WII | 4060.4 | 4064.786 | 0 | 45.995 | 0.161 | -2017.2 | 0.349 | 0 | 0 | 13 |
| FF * Silt * VRM * WII | 4060.184 | 4064.57 | 0 | 46.051 | 0.18 | -2017.092 | 0.349 | 0 | 0 | 13 |
| FF * SOMO31 * VRM * WII | 4060.185 | 4064.57 | 0 | 46.053 | 0.18 | -2017.092 | 0.349 | 0 | 0 | 13 |
| FF * SOMO31 * SPIN * VRM | 4060.185 | 4064.571 | 0 | 46.155 | 0.18 | -2017.093 | 0.349 | 0 | 0 | 13 |
| DEM * FF * VRM * WII | 4060.314 | 4064.7 | 0 | 46.214 | 0.17 | -2017.157 | 0.349 | 0 | 0 | 13 |
| DEM * SOMO31 * VRM * WII | 4060.306 | 4064.692 | 0 | 46.42 | 0.167 | -2017.153 | 0.349 | 0 | 0 | 13 |
| FF * Silt * VRM * WAT | 4060.186 | 4064.572 | 0 | 46.462 | 0.18 | -2017.093 | 0.349 | 0 | 0 | 13 |
| SPIN * VRM * WAT * WII | 4060.423 | 4064.809 | 0 | 47.212 | 0.166 | -2017.212 | 0.349 | 0 | 0 | 13 |
| Silt * SOMO31 * VRM * WAT | 4060.196 | 4064.581 | 0 | 48.63 | 0.18 | -2017.098 | 0.349 | 0 | 0 | 13 |
| DEM * Silt * VRM * WAT | 4060.452 | 4064.837 | 0 | 48.798 | 0.169 | -2017.226 | 0.349 | 0 | 0 | 13 |
| Silt * SOMO31 * VRM * WII | 4060.193 | 4064.578 | 0 | 49.016 | 0.18 | -2017.096 | 0.349 | 0 | 0 | 13 |
| SOMO31 * VRM * WAT * WII | 4060.193 | 4064.579 | 0 | 49.142 | 0.18 | -2017.097 | 0.349 | 0 | 0 | 13 |
| DEM * SPIN * VRM * WII | 4060.288 | 4064.673 | 0 | 49.198 | 0.179 | -2017.144 | 0.349 | 0 | 0 | 13 |
| DEM * SOMO31 * VRM * WAT | 4060.467 | 4064.852 | 0 | 49.637 | 0.171 | -2017.233 | 0.349 | 0 | 0 | 13 |
| Silt * VRM * WAT * WII | 4060.431 | 4064.816 | 0 | 49.686 | 0.173 | -2017.215 | 0.349 | 0 | 0 | 13 |
| Silt * SPIN * VRM * WII | 4060.694 | 4065.079 | 0 | 50.094 | 0.162 | -2017.347 | 0.349 | 0 | 0 | 13 |
| Silt | 4070.826 | 4071.26 | 0.015 | 50.297 | 0.057 | -2031.413 | 0.351 | 8 | 0.8 | 4 |
| FF * SPIN * VRM * WII | 4061.624 | 4066.01 | 0 | 50.922 | 0.141 | -2017.812 | 0.349 | 0 | 0 | 13 |
| FF * Silt * SOMO31 * VRM | 4061.911 | 4066.296 | 0 | 51.03 | 0.136 | -2017.955 | 0.349 | 0 | 0 | 13 |
| DEM * SPIN * VRM * WAT | 4060.882 | 4065.267 | 0 | 51.453 | 0.16 | -2017.441 | 0.349 | 0 | 0 | 13 |
| DEM * FF * VRM * WAT | 4060.689 | 4065.074 | 0 | 52.505 | 0.169 | -2017.344 | 0.349 | 0 | 0 | 13 |
| DEM * FF * SPIN * VRM | 4062.442 | 4066.828 | 0 | 53.836 | 0.134 | -2018.221 | 0.35 | 0 | 0 | 13 |
| DEM * FF * SOMO31 * VRM | 4061.093 | 4065.478 | 0 | 54.059 | 0.161 | -2017.546 | 0.349 | 0 | 0 | 13 |
| DEM * Silt * SOMO31 * VRM | 4062.353 | 4066.739 | 0 | 54.502 | 0.136 | -2018.177 | 0.35 | 0 | 0 | 13 |
| WAT | 4074.169 | 4074.604 | 0.052 | 55.043 | 0.038 | -2033.084 | 0.351 | 66 | 6.6 | 4 |
| FF * SOMO31 * VRM * WAT | 4063.413 | 4067.798 | 0 | 57.894 | 0.128 | -2018.706 | 0.35 | 0 | 0 | 13 |

***P. hermannsburgensis microsatellite bootstrap results***

| **Surface** | **Avg. AIC** | **Avg. AICc** | **Avg. weight** | **Avg. rank** | **Avg. R^2^m** | **Avg. LL** | **Avg. RMSE** | **n** | **Top model (%)** | **k** |
| --- | --- | --- | --- | --- | --- | --- | --- | --- | --- | --- |
| Clay | 2680.962 | 2681.397 | 0.575 | 1.346 | 0.025 | -1336.481 | 0.306 | 793 | 79.3 | 4 |
| SOMO31 | 2684.867 | 2685.302 | 0.14 | 3.27 | 0.013 | -1338.434 | 0.306 | 118 | 11.8 | 4 |
| IBD | 2684.836 | 2684.964 | 0.145 | 3.34 | 0.002 | -1340.418 | 0.306 | 68 | 6.8 | 2 |
| VRM | 2685.835 | 2686.27 | 0.069 | 4.141 | 0.011 | -1338.918 | 0.306 | 14 | 1.4 | 4 |
| Clay * SOMO31 | 2685.914 | 2687.172 | 0.039 | 4.199 | 0.011 | -1335.957 | 0.306 | 7 | 0.7 | 7 |
| Clay * VRM | 2686.615 | 2687.873 | 0.024 | 4.918 | 0.013 | -1336.307 | 0.306 | 0 | 0 | 7 |
| SOMO31 * VRM | 2690.035 | 2691.293 | 0.007 | 6.851 | 0.014 | -1338.017 | 0.306 | 0 | 0 | 7 |
| Clay * SOMO31 * VRM | 2693.236 | 2695.795 | 0.001 | 7.935 | 0.014 | -1336.618 | 0.306 | 0 | 0 | 10 |

**Appendix S9. *ResistanceGA* transformations.**

Transformations for optimised single surfaces and for layers used to create final composite surfaces for the best performing model across marker types and species.

***1. N. timealeyi* SNPs: multi-surface optimisation transformations for top-ranked model**

| SOMO29 | FOR | VRM |
| --- | --- | --- |
| 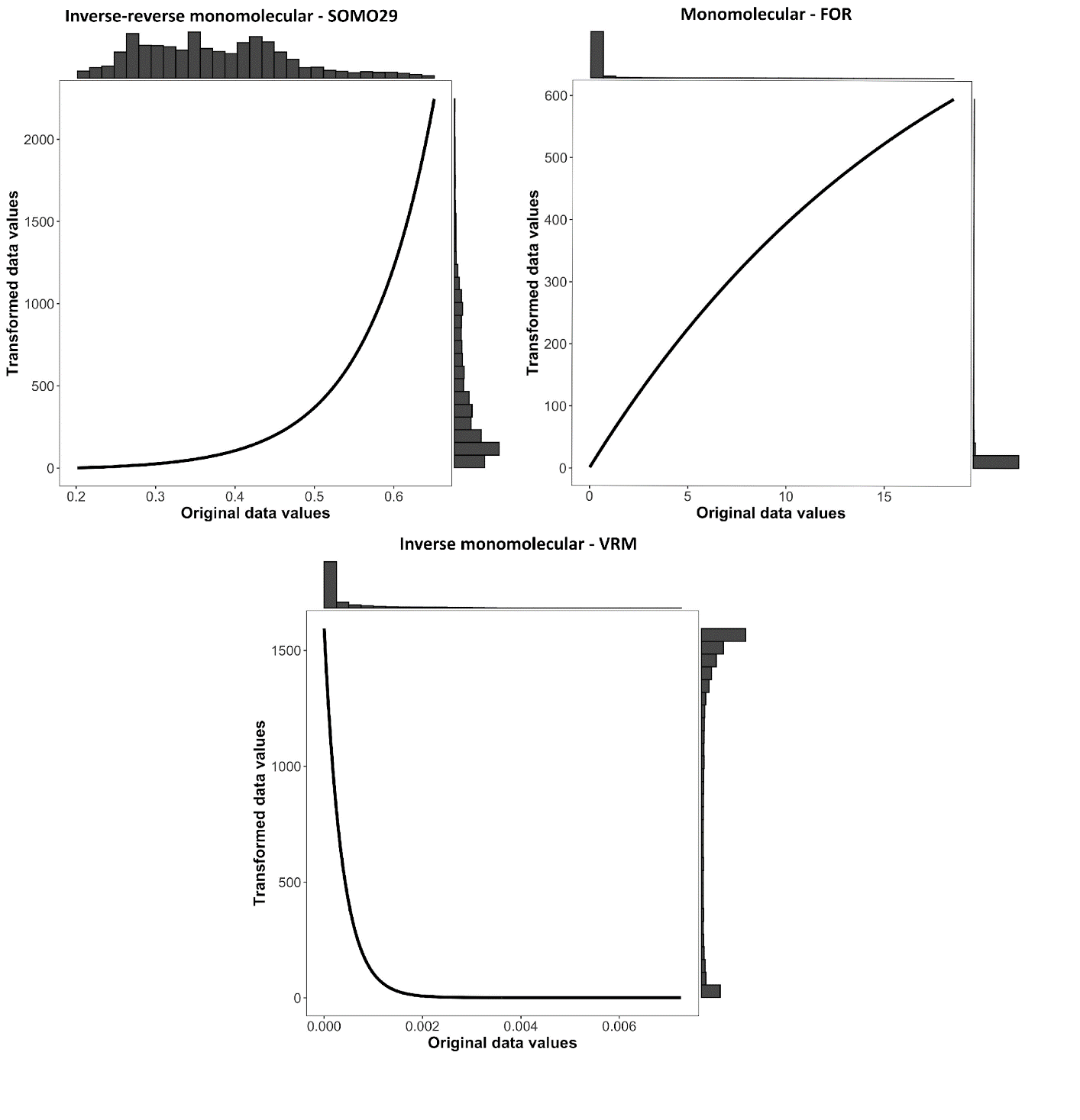 | 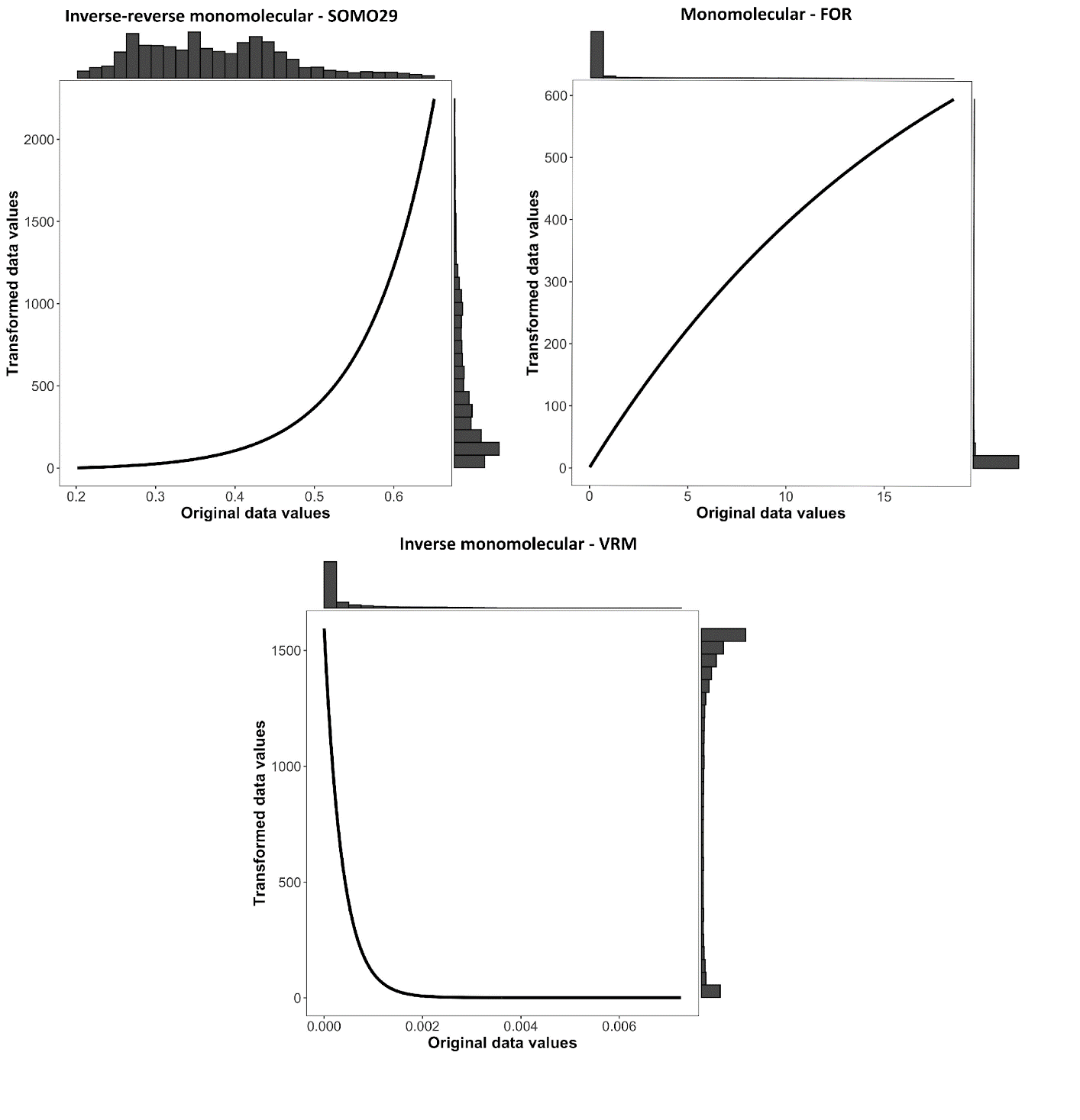 | 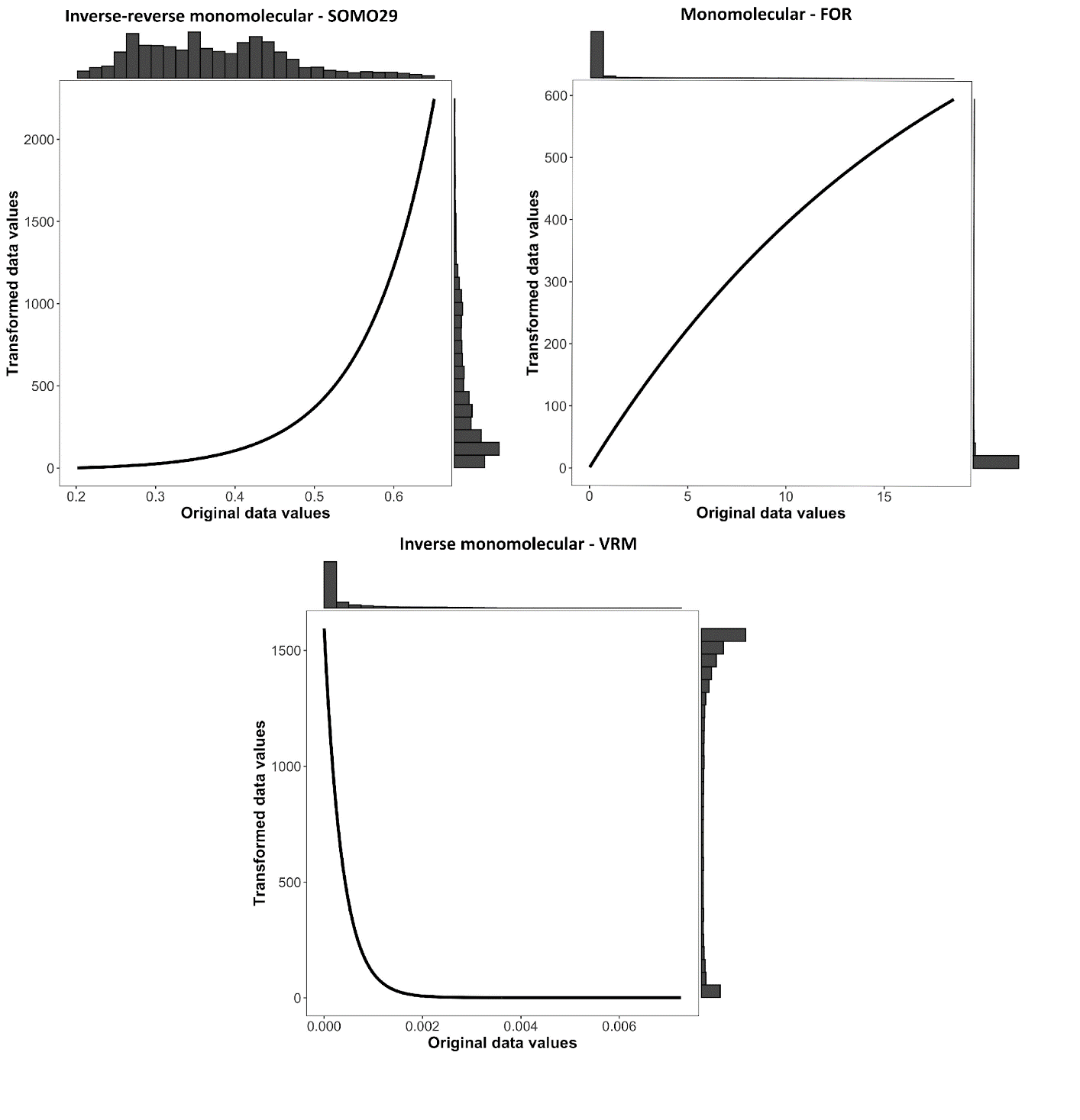 |

**2. *N. timealeyi* microsatellites: single-surface optimisation transformation for top-ranked model**

| CF |
| --- |
| 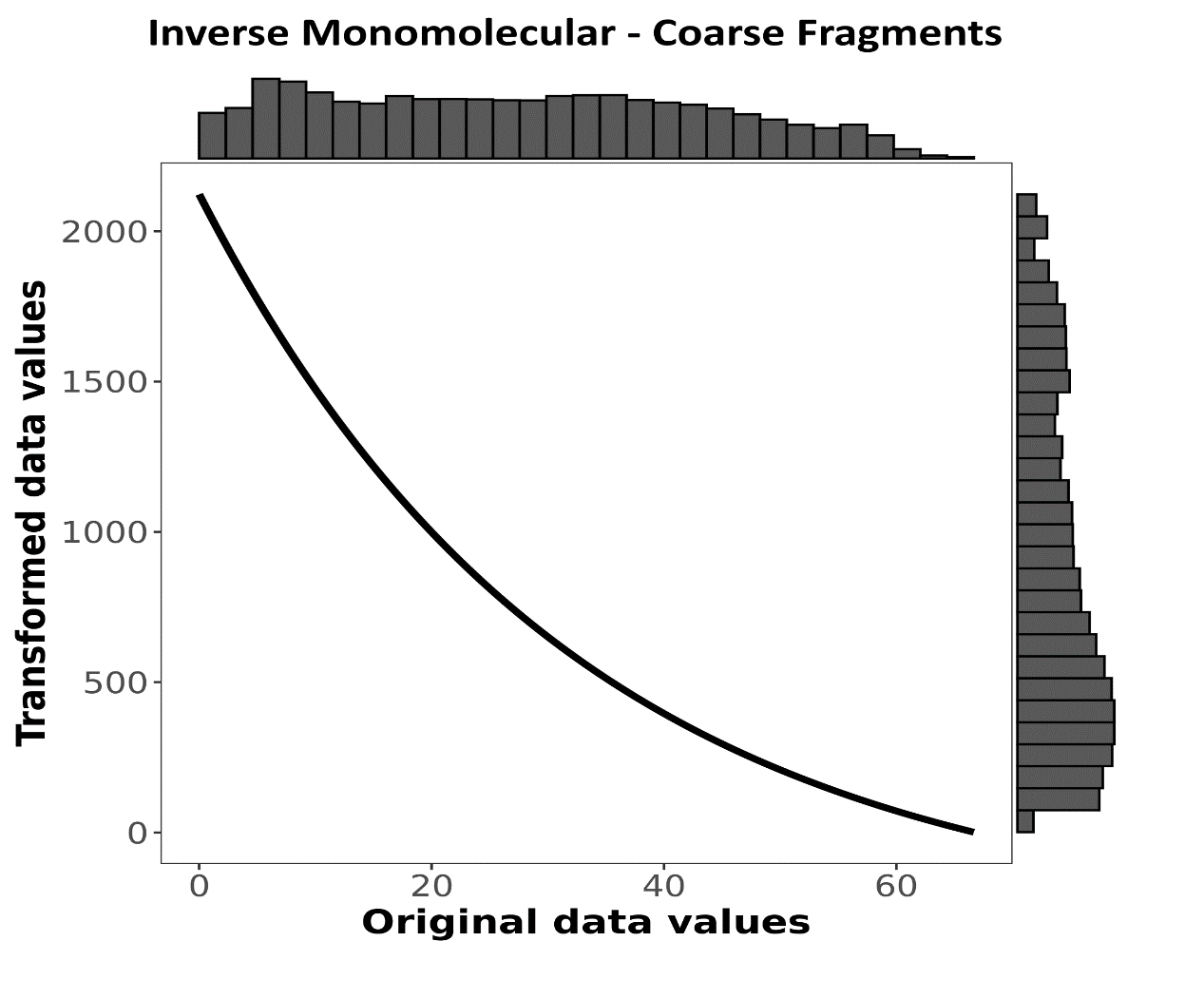 |

***3. P. chapmani* SNPs: multi-surface optimisation transformations for top-ranked model**

| SOMO29 | WII |
| --- | --- |
| 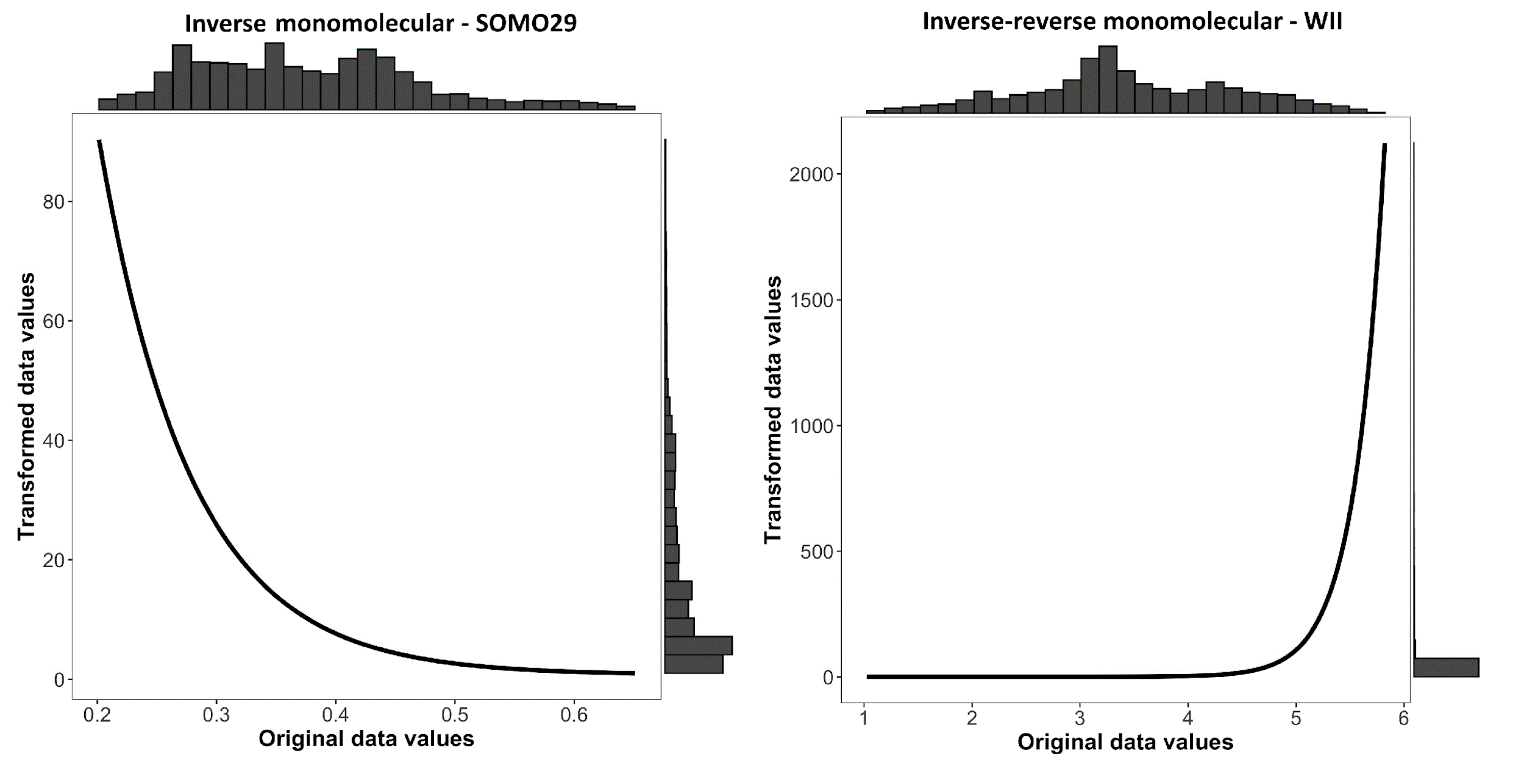 | 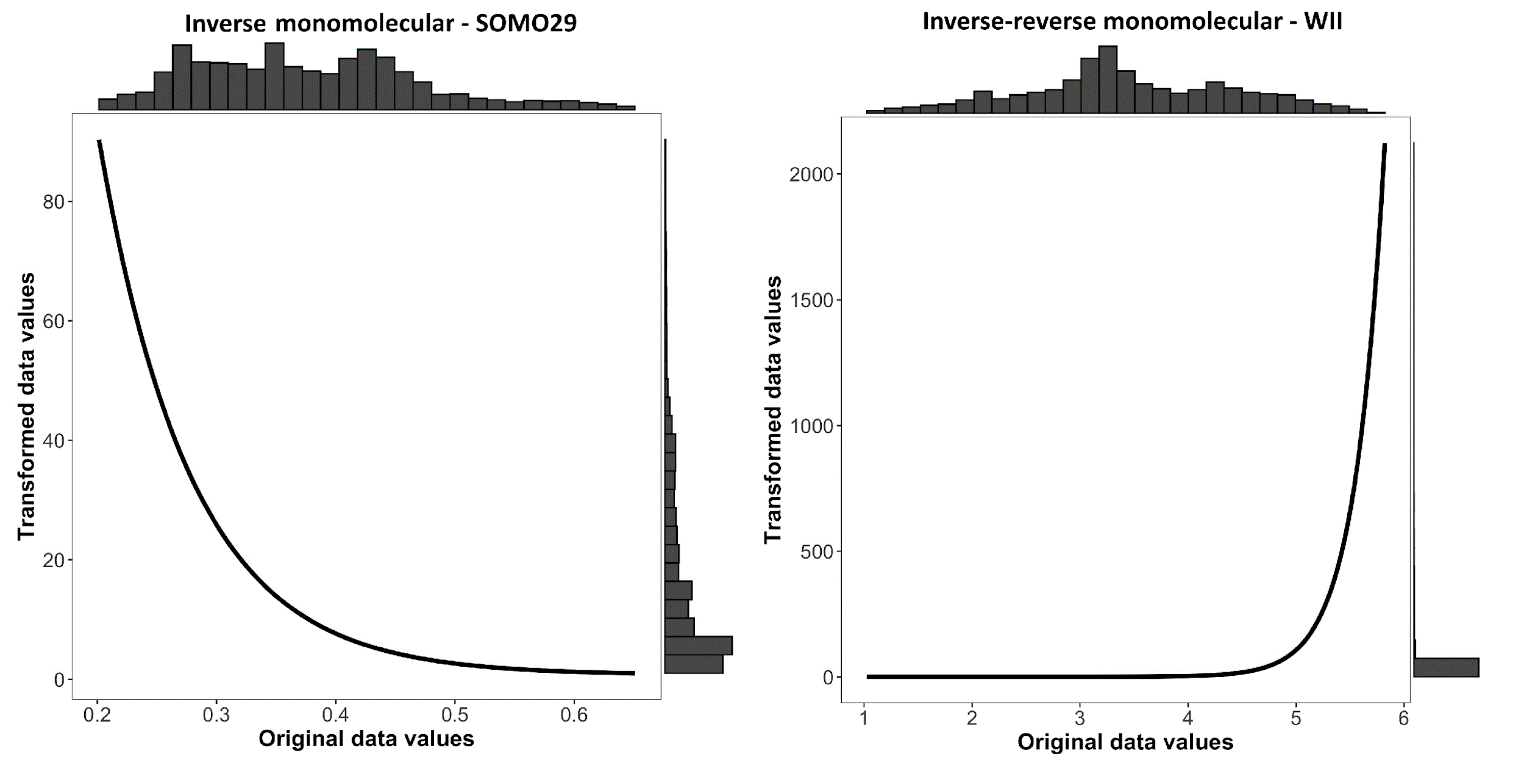 |

**4. *P. chapmani* microsatellites: single-surface optimisation transformation for top-ranked model**

| CF |
| --- |
| 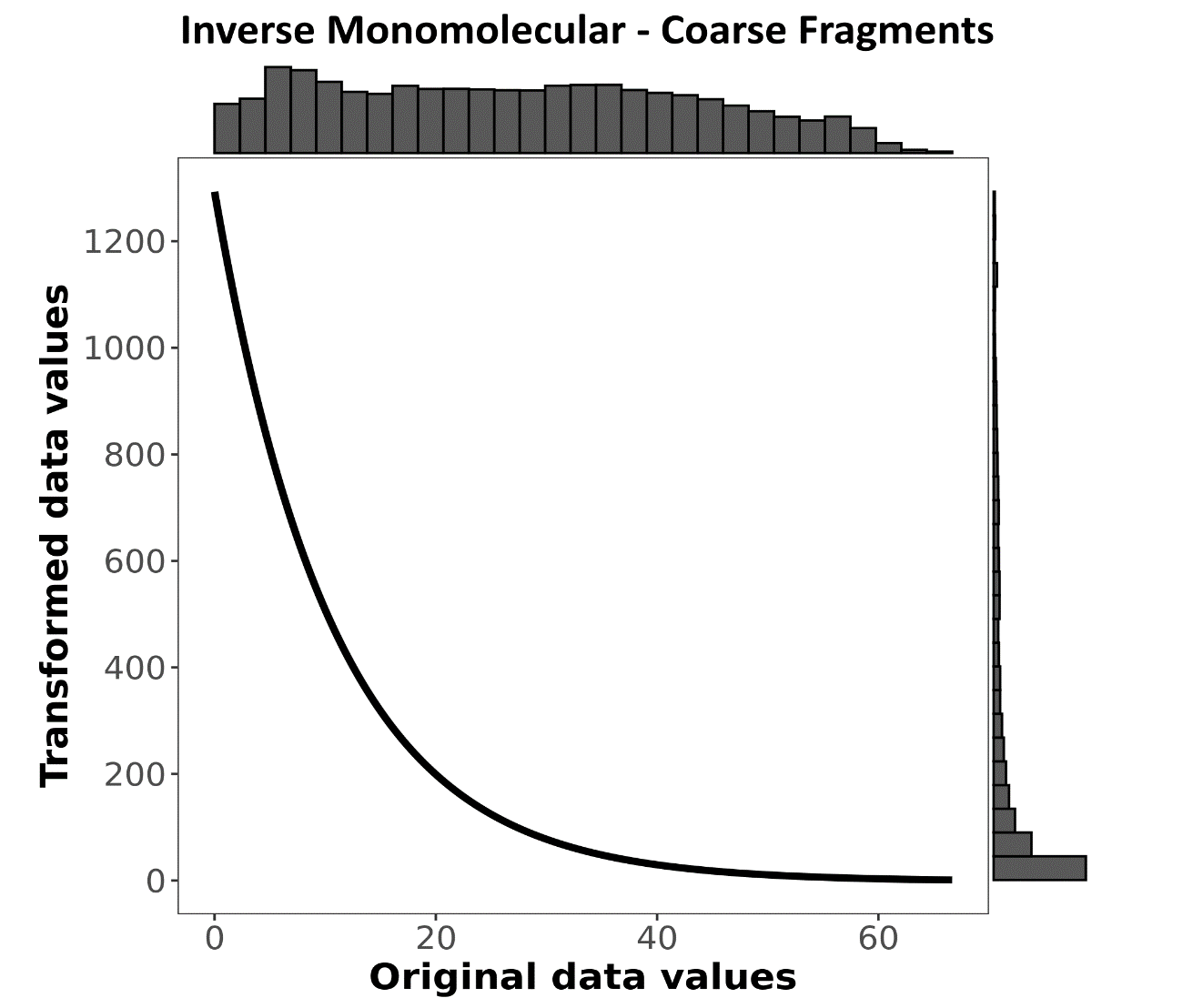 |

**5. *P. hermannsburgensis* SNPs: single-surface optimisation transformation for top-ranked model**

| VRM |
| --- |
| **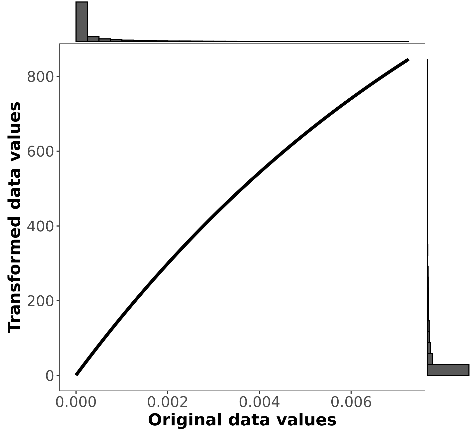** |

**6. *P. hermannsburgensis* microsatellites: single-surface optimisation transformation for top-ranked model**

| Clay |
| --- |
| *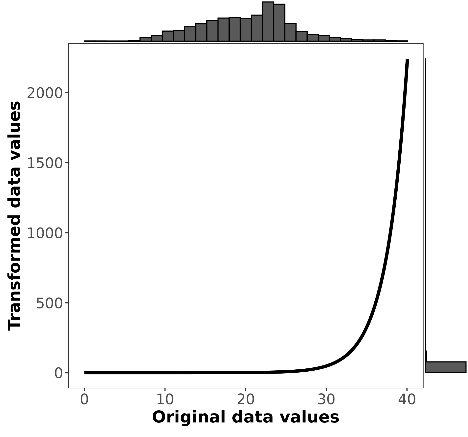* |

**References**

ESRI. (2018). *ArcGIS Desktop: Release 10.6.* Redlands, CA: Environmental Systems Research Institute.

Evans, J. (2020). *SpatialEco*. R package.

Furby, S. (2018). *Woody vegetation extent and change 1972-2018: Western Australia – whole state product 2018*. Western Australia: Update of the Land Monitor II project.

Furby, S., Wallace, J., & Caccetta, P. (2007). *Monitoring sparse perennial vegetation cover over Australia using sequences of Landsat imagery*. Presented at the International Conference on Environmental Informatics, Bangkok, Thailand. Bangkok, Thailand.

Gallant, J., Wilson, N., Dowling, T., Read, A., & Inskeep, C. (2011). *SRTM-derived 1 second Digital Elevation Models*. Canberra, Australia: Geoscience Australia.

Hartig, F. (2022). *DHARMa: Residual diagnostics for hierarchical (multi-level/mixed) regression models*. R package.

Harwood, T. D. (2019). *9s climatology for continental Australia 1976-2005: BIOCLIM variable suite*. Data Collection: CSIRO.

Harwood, T. D., Donohue, R., Harman, I., McVicar, T., Ota, N., Perry, J., & Williams, K. (2016). *9s climatology for continental Australia 1976-2005: Summary variables with elevation and radiative adjustment*. Data Collection: CSIRO.

Hijmans, R. J. (2020). *raster: Geographic data analysis and modeling*. R package.

Holmes, K., Griffen, T., & Odgers, N. (2014). *Soil and Landscape Grid Digital Soil Property Maps for Western Australia (3" resolution)*. Data Collection: CSIRO.

Landgate. (2012). *Medium-scale topographic database (GIS dataset—Inland flat and coastal flat polygon features)*. Western Australian Land Information Authority.

Landgate. (2017). *Medium-scale topographic database (GIS dataset—Linear and polygon features)*. Western Australian Land Information Authority.

Landgate. (2019). *Medium-scale topographic database (GIS dataset—Point features)*. Western Australian Land Information Authority.

Li, F., Jupp, D. L. B., Thankappan, M., Lymburner, L., Mueller, N., Lewis, A., & Held, A. (2012). A physics-based atmospheric and BRDF correction for Landsat data over mountainous terrain. *Remote Sensing of Environment*, *124*, 756–770. doi: 10.1016/j.rse.2012.06.018

North Australia and Rangelands Fire Information. (2019). *Modis annual fire scar images (2000—2018)*. Charles Darwin University, Darwin: Centre for Bush Fire Research.

O’Brien, L. (2020). *slga: Data access tools for the soil and landscape grid of Australia.* R package.

Rampant, P., Zdunic, K., & Burrows, N. (2019). UAS and Landsat imagery to determine fuel condition for fire behaviour prediction on spinifex hummock grasslands of arid Australia. *International Journal of Remote Sensing*, *40*(24), 9126–9139. doi: 10.1080/01431161.2019.1651950

Sappington, J. M., Longshore, K. M., & Thompson, D. B. (2007). Quantifying landscape ruggedness for animal habitat analysis: A case study using bighorn sheep in the Mojave Desert. *Journal of Wildlife Management*, *71*(5), 1419–1426. doi: 10.2193/2005-723

Shaw, R. E. (2022). *RobynSh/NorthernQuolls_LifeHistory2Landscape: R code for Paper*. Zenodo. doi: 10.5281/zenodo.7005247

Shaw, R. E., Spencer, P. B., Gibson, L. A., Dunlop, J. A., Kinloch, J. E., Mokany, K., … Ottewell, K. M. (2022). Linking life history to landscape for threatened species conservation in a multiuse region. *Conservation Biology*, *00*, 00–00. doi: 10.1111/cobi.13989

Tremblay, A., & Ransijn, J. (2020). *LMERConvenienceFunctions: Model selection and post-hoc analysis for (G)LMER models*. R package.

Wilford, J. (2012). A weathering intensity index for the Australian continent using airborne gamma-ray spectrometry and digital terrain analysis. *Geoderma*, *183–184*, 124–142. doi: 10.1016/j.geoderma.2010.12.022

Wilford, J., & Roberts, D. (2019). *Weathering Intensity Model of Australia*. Canberra, Australia: Geoscience Australia.

Xu, T., & Hutchinson. (2011). *ANUClim Version 6.1 User Guide*. Canberra: The Australian National University, Fenner School of Environment and Society.
